# Meet our editorial board members from Asia, South America, Oceania and Africa

**DOI:** 10.1111/iwj.70121

**Published:** 2024-11-18

**Authors:** Keith Harding, Douglas Queen

**Affiliations:** ^1^ International Wound Journal Oxford UK

In our recent editorials, we discussed the expansion of our Editorial Board^1^, ^2^, ^3^ and introduced you to some of our members. We promised a series of further editorials introducing our wider membership. This is the last in a series of three editorials providing insight into the group of outstanding and distinguished individuals that comprise our board. We have created the largest most internationally diverse board to greatly increase the capabilities and expertise of the journal.

## 1 ASIAN, SOUTH AMERICAN, OCEANIAN AND AFRICAN EDITORIAL BOARD MEMBERS

Please meet the Editorial Board Members for the *International Wound Journal* covering Asia, South America, Oceania and Africa.

**Figure:**
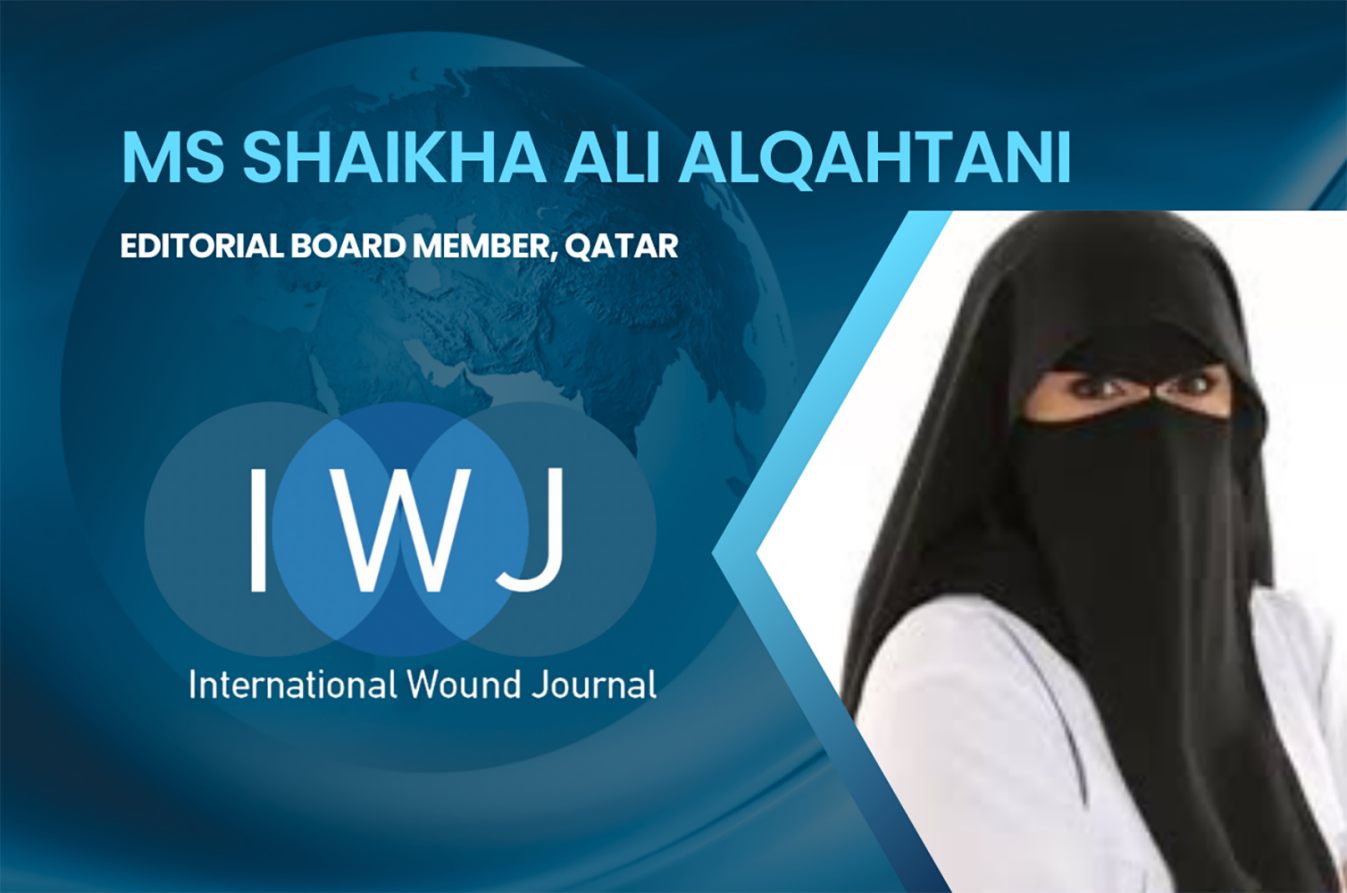
Ms. Shaikha Ali AlQahtani, Qatar

Ms. Shaikha Al Qahtani is the first Qatari Director of Nursing in Wound Care Management at Hamad Medical corporation (HMC). She oversees Nursing and Wound Care Management for ambulatory care services in the hospital's OPD and surgery unit, as well as Hamad Medical Corporation's (HMC) Bone and Joint Center and Fahad Bin Jassim Dialysis Center. She is a member of the American College of Wound Care and Tissue Repair (AWCT). Ms. Al Qahtani previously worked as a subspecialty wound care nurse in the Patient and Family Education Unit at HGH, also covering Women's Hospital.

She has conducted many wound care awareness programs for schools and held numerous wound care workshops for students and faculty at the University of Calgary (UCQ). Shaikha has also attended and participated in many international courses and conferences.

Ms. Al Qahtani completed her Diploma in Nursing in 2007 through the High Institute of Nursing—Doha, Qatar, and completed an International InterProfessional Wound Care course from the University of Toronto (IIWCC) in 2009. Ms. Al Qahtani received her BSN in 2013 from the University of Calgary—Qatar and followed that with a Masters in Science of Skin Integrity Skills and Treatment from the University of Hertfordshire (2014). She recently completed her Master of Nursing—Thesis at the University of Calgary—Qatar (2016).

She is the first UCQ student to complete the MN thesis program. Her research is already making an impact—the model of collaborative interprofessional practice in the outpatient wound clinic at HMC was rated very highly by her research respondents. Shaikha is disseminating her findings about the contributions of a well‐developed interprofessional team that supports holistic approaches to healing wounds. She has also published a number of articles and abstracts and has presented at national and international conferences.

**Figure:**
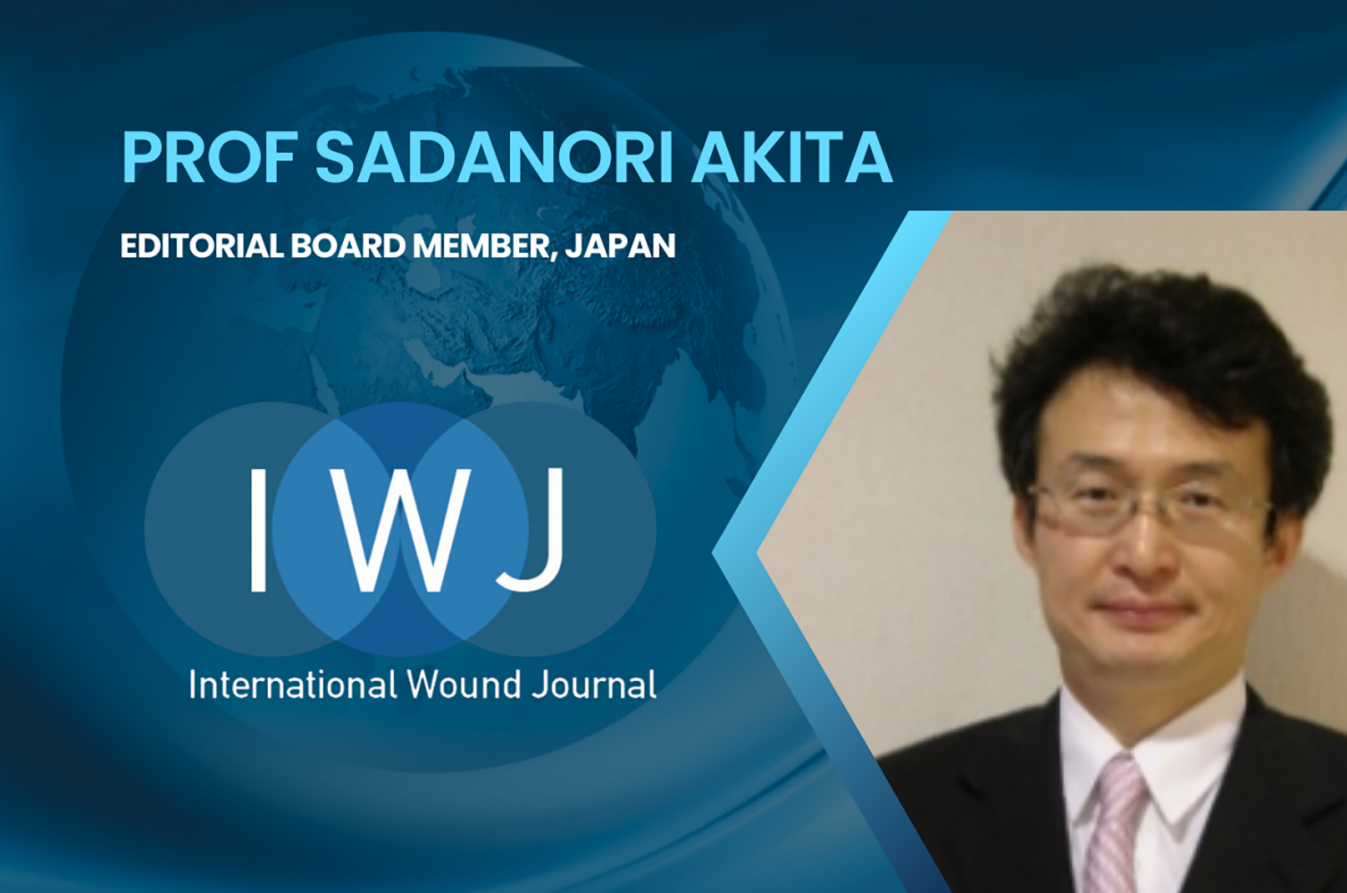
Professor Sadanori Akita, Japan

Dr. Akita served as a full professor and chief of the Department of Plastic Surgery, Wound Repair, and Regeneration at Fukuoka University School of Medicine from 2016 to 2021. He is currently a full professor at Fukushima Medical University. He completed his residency in plastic surgery at Nagasaki University Hospital and earned his PhD from the Graduate School of Nagasaki University, specializing in plastic and reconstructive surgery.

He is a past President of the World Union of Wound Healing Societies (2012–2016). Currently, he is the president of the Asian Wound Care Association (AWCA). His research interests include cytokines and stem cells in wound healing, the treatment of difficult wounds such as radiation injuries, regenerative tissue enhancement for HIV‐drug‐related wasting patients, reconstructive surgery, burns, craniofacial surgery, and the treatment of hemangiomas/vascular malformations. Dr. Akita is an active member of the editorial boards of over 8 renowned journals, including *Wound Repair and Regeneration* (where he also serves as a senior editor). He is a regular reviewer for journals such as *Plastic and Reconstructive Surgery*, *Lancet*, *Nature* and *Annals of Surgery*. His work aims to bridge basic molecular science with clinically oriented solutions to mechanisms and functional problems, incorporating aesthetic considerations through a translational approach. Dr. Akita actively engages with the international community through lectures and workshops.

**Figure:**
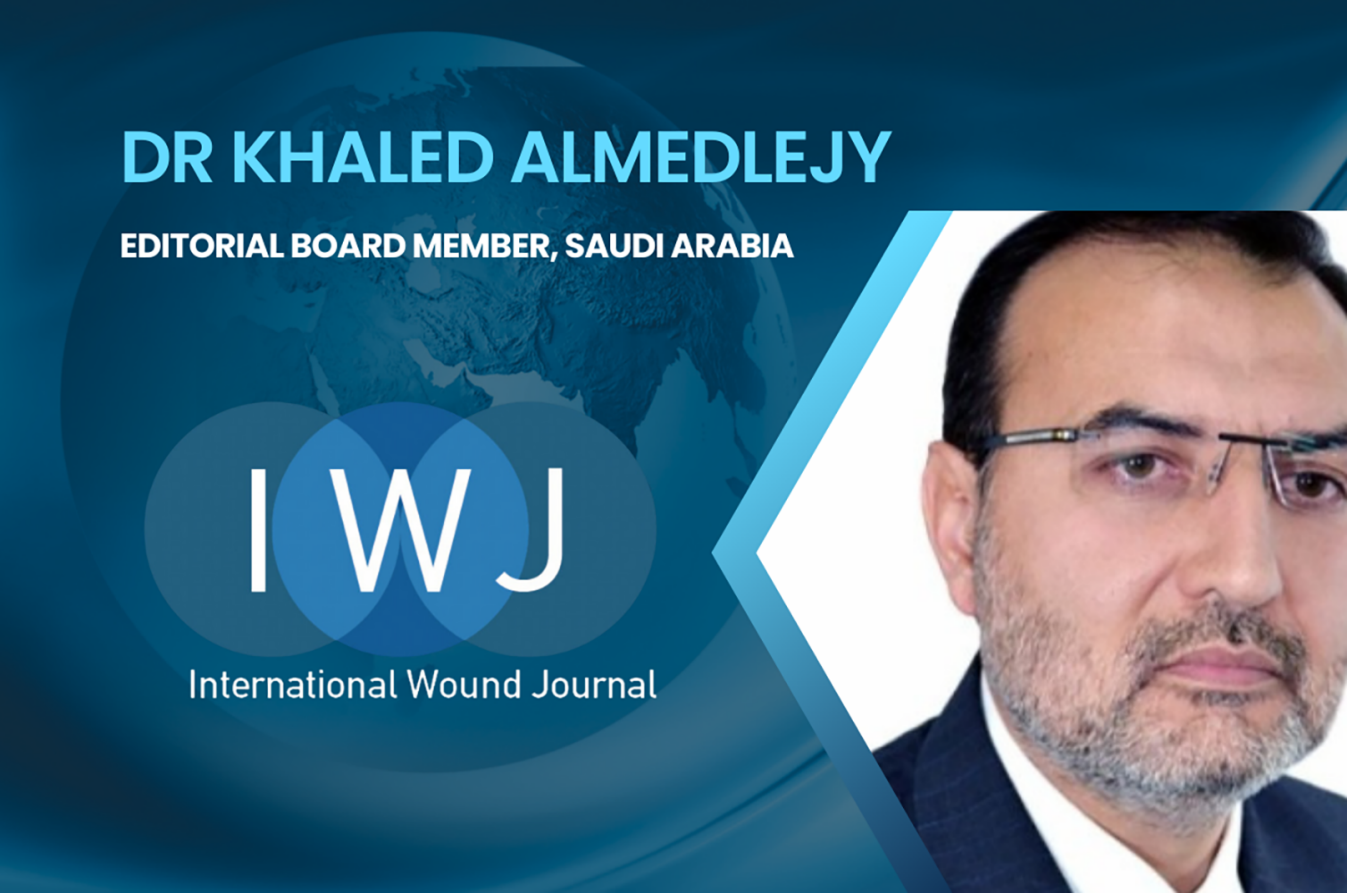
Dr. Khaled Almedlejy, Saudi Arabia

Khaled obtained his Doctor of Medicine from Damascus University, Syria (1992). He went on to obtain a Master's degree in general surgery, Syria (1998), and completed the International Inter‐Professional Wound Care Course (IIWCC), Toronto University, 2016, for 1 year.

He is currently the Head, The Diabetic Foot Unit, King Abdul Aziz Specialist Hospital (KAASH), Taif, KSA. He also practiced as a Specialist in General Surgery, KAASH, Taif, KSA (2008–2010), and a Specialist in General Surgery, Syria (1999–2008). Prof. Khaled has extensive training in diabetic foot obtained both regionally and internationally attending over 10 courses.

He is a frequent speaker at conferences around his region, providing insight and training on management of the diabetic foot. He supervises academics on their master's courses. He received the Al‐Basil Highest Award in Research in General Surgery for a research project on ‘In Roux‐En –Y Syndrome’.

**Figure:**
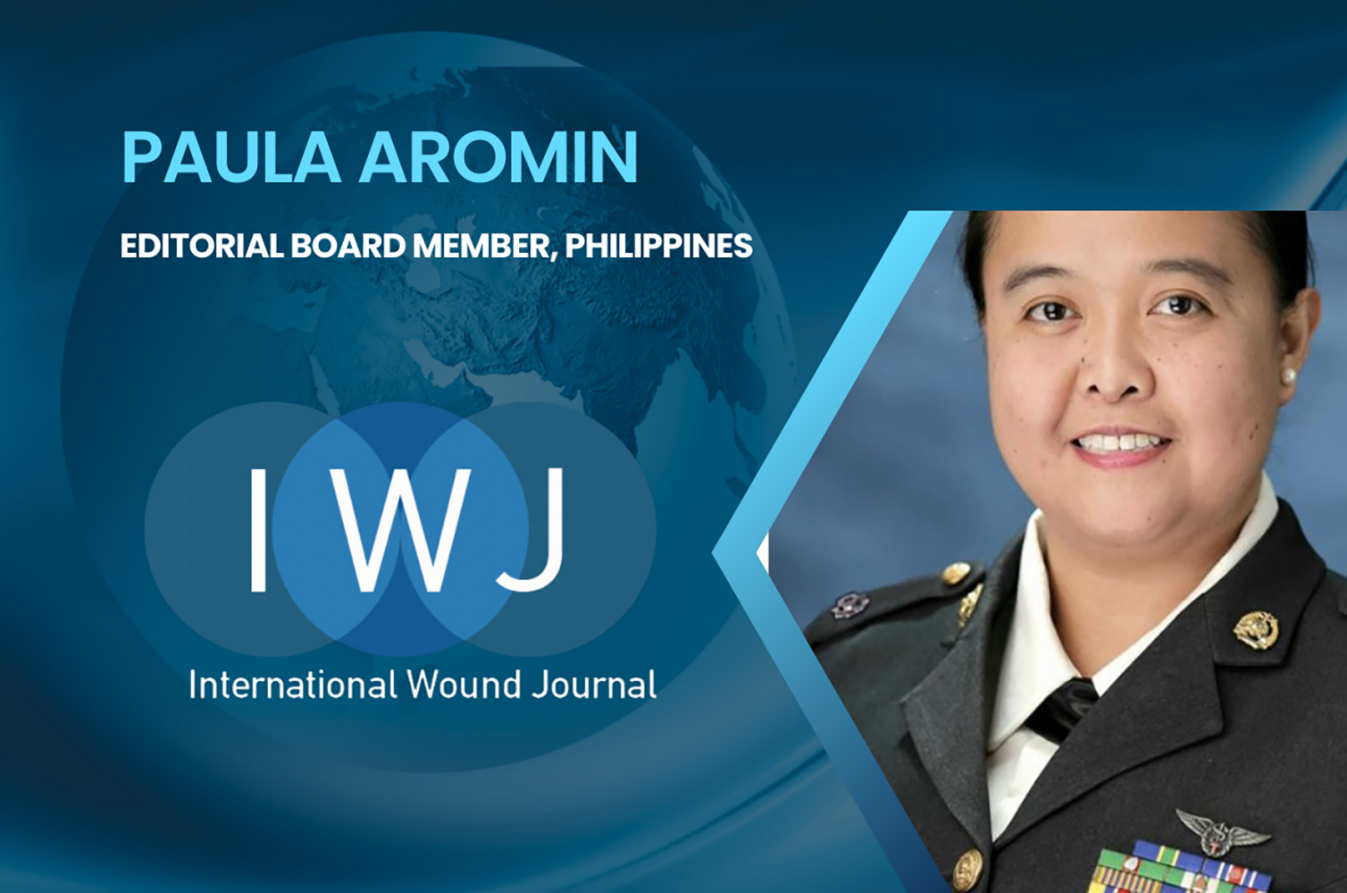
Paula Aromin, Philippines

Paula obtained a BS Nursing, University of the Philippines, Manila, and Master's in Public Management, Ateneo De Manila University.

International Interprofessional Wound Care Course—Canada (2019). Her career has mainly been in nursing within the Air Force in the Philippines, through a series of positions. Paula is currently a Military Flight Nurse/Head Nurse, OB‐Gyne Ward/Head Nurse, Pedia Ward at the Air Force General Hospital, Colonel Jesus Villamor Air Base, Pasay City.

Aside from the usual designations, she had also been given additional special duties to perform: As a flight nurse in the Philippine Air Force, she had successfully aeromedically evacuated numerous patients from all over the Philippines, flying over the dangerous mountains of the north to the hot areas of the south. She had also done multiple tours of duties at war areas such as Jolo, Sulu and Marawi.

She is the International Coordinator and Facilitator, Woundpedia Wound Care Basic and Intermediate Course Manila since 2016, where she facilitates and coordinates the arrival of international subject matter experts from the USA, Canada and Bahrain to provide free wound care courses to health care professionals all over the Philippines. There are over 500 graduates from this course in the Philippines. Future work involves seeing more international collaborations to support and develop local interprofessional subject matter experts for the sustainability of the project.

**Figure:**
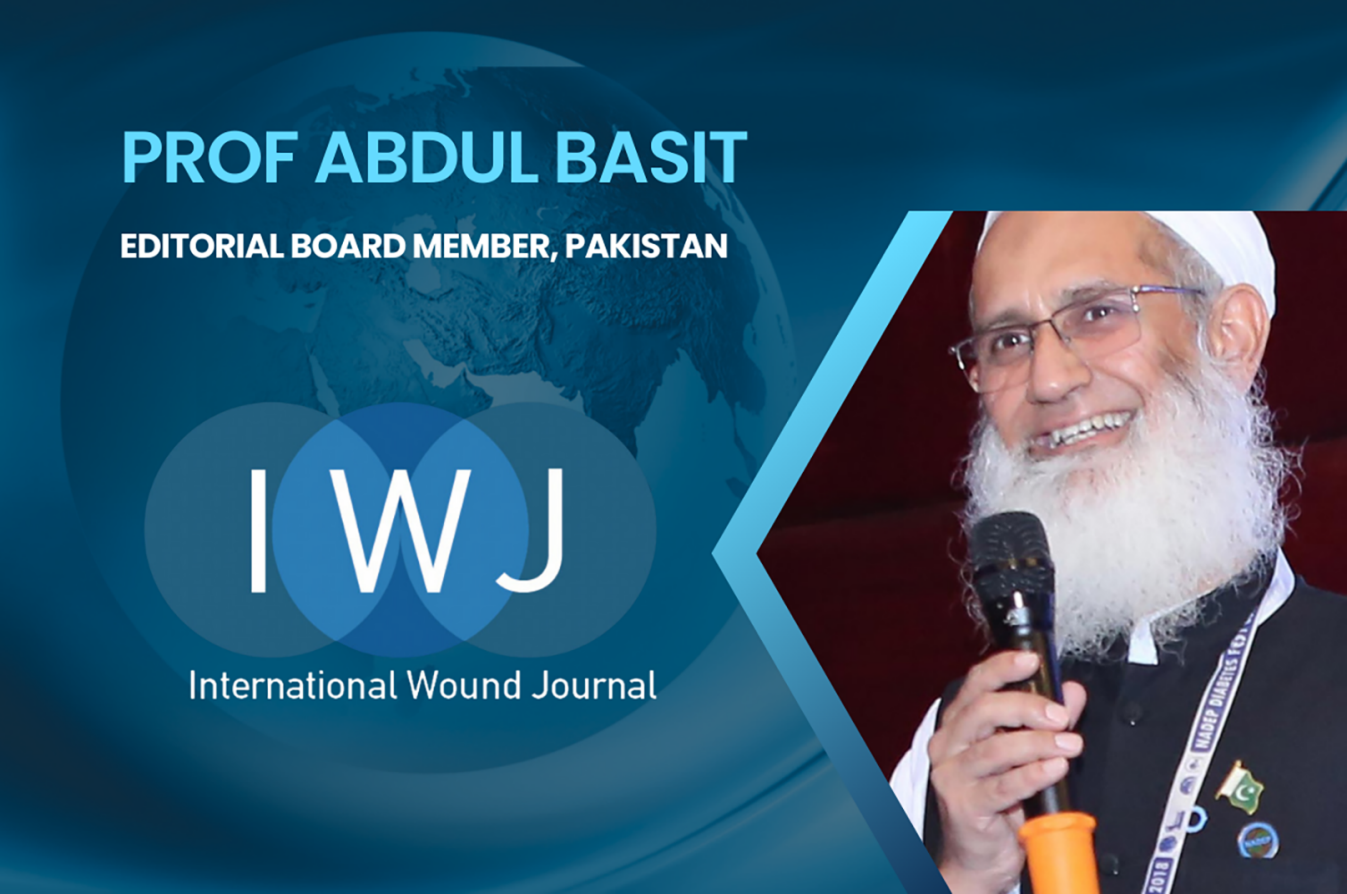
Professor Abdul Basit, Pakistan

Secretary General, Diabetic Association of Pakistan (DAP) and Head, WHO Collaborating Centre for ‘Treatment, Education & Research in Diabetic Pregnancies’.

- President, Diabetes in Asia Study Group (DASG).
- Founder Member, Pakistan Endocrine Society (PES).
- Vice Chairman, Health Research Advisory Board (HRAB).
- Chairman, Pakistan Working Group on the Diabetic Foot (PWGDF).
- Founder Member, National Association of Diabetes Educators (NADEP).
- Former‐chair, IDF MENA Region.
- Ex‐Editor, *Journal of Diabetology* (JOD), official journal of DASG.
- More than 240 research papers have been published.

**Figure:**
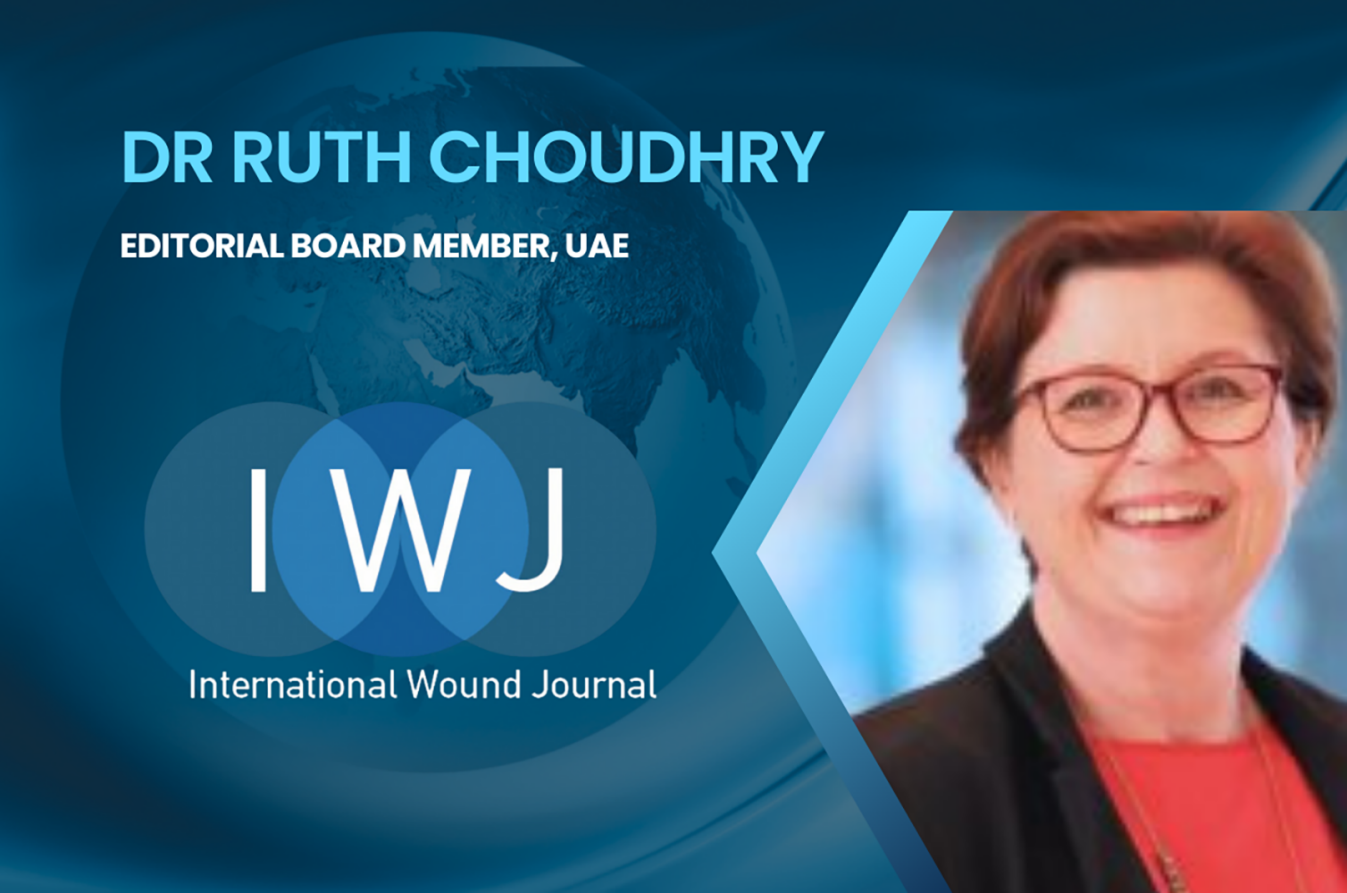
Dr. Ruth Choudhry, UAE

Ruth qualified as a podiatrist in 1992 in the UK and has since specialized in the assessment, treatment and management of the high‐risk foot. She worked for the UK National Health Service for over 20 years before moving to Abu Dhabi in 2014.

Ruth undertook a Master's degree in advanced practice to expand her skills in lower limb vascular assessment and surgical debridement. She has established a number of multi‐disciplinary diabetic foot teams which have resulted in the reduction of lower limb amputations and improved care of people with diabetic foot disease.

Ruth is currently the senior podiatrist at Sheikh Shakhbout Medical City in Abu Dhabi, working as a member of the vascular and limb salvage team. Here, she provides care for diabetic foot emergencies, pressure injuries and patients with peripheral vascular disease. She also established and runs a busy diabetic foot clinic, seeing patients with complex foot problems and trying to prevent lower limb amputation and help those recover from diabetic foot complications. Ruth is an international speaker on the assessment and care of the diabetic foot disease, and she is passionate about educating people about the diabetic foot, to reduce the risk of amputation and improve health outcomes, keeping people mobile and independent.

**Figure:**
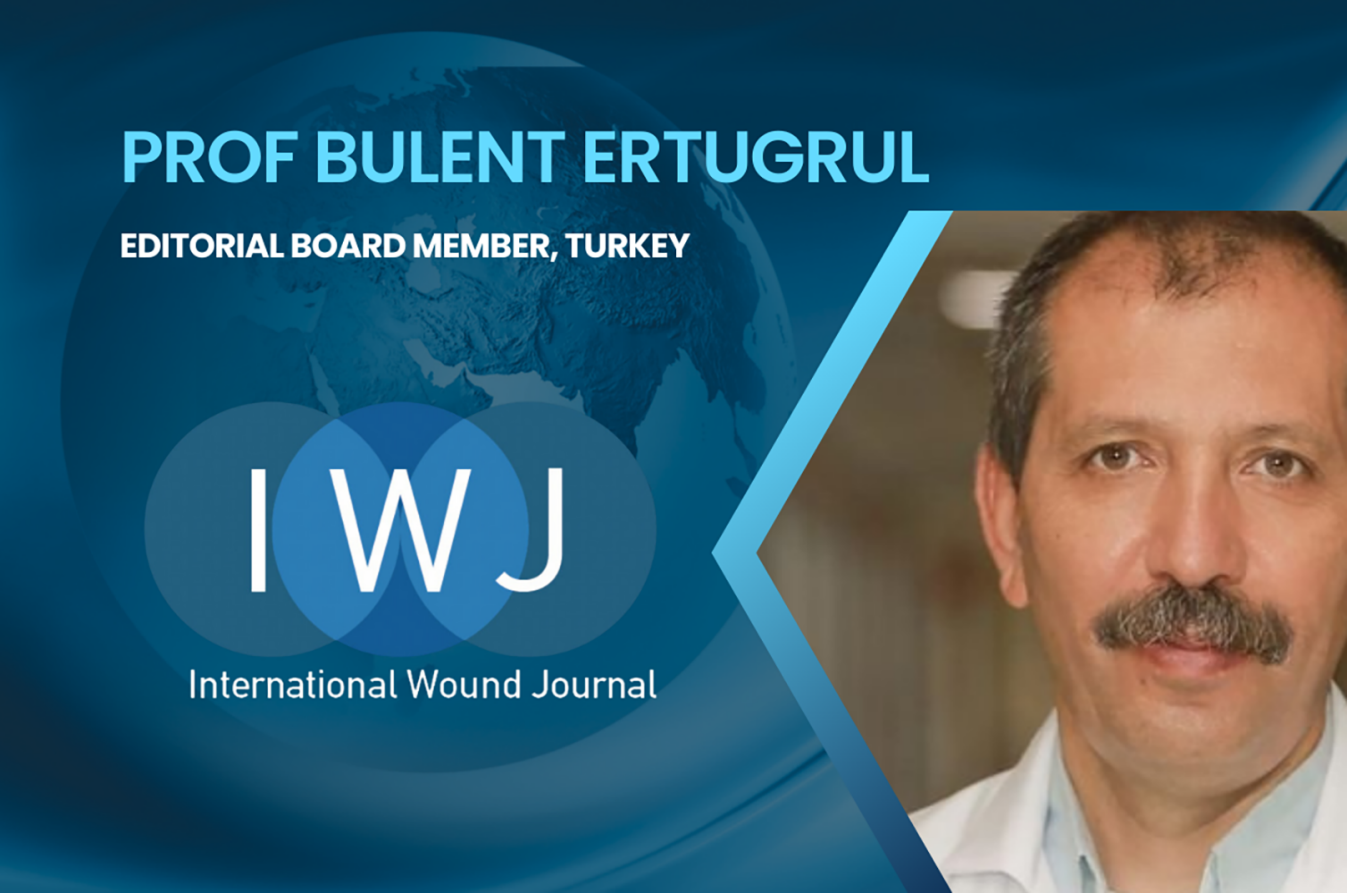
Professor Bulent Ertugrul, Turkey

Dr. Ertugrul graduated as a specialist in Infectious Diseases and Clinical Microbiology from the University of İstanbul School of Medicine. He started to work in Microbiology at Adnan Menderes University Faculty of Medicine, Department of Infectious Diseases and Clinical Microbiology in 2004. He received the title of Professor in the same department in 2017. During this working period, he established and was responsible for the Nazlı—Selim Eren Chronic Wound and Infections Care Unit at the University Hospital in 2016. He retired in 2020 and founded GEDA Wound Care Clinic in Izmir and still works there.

He has published more than 80 national and international research articles and book chapters and more than 100 congress and symposium papers. He was invited and worked as an observer faculty member at Oxford University Nuffield Orthopaedic Center, Bone Infections Department, in 2015 and at Zurich University Balgrist University Hospital for 1 month in 2019. His most important areas of interest are diabetic foot infections, orthopaedic infections, skin and soft tissue infections, rational antibiotic use and antibiotic resistance.

Turkish Medical Association (TTB), Turkish Clinical Microbiology and Infectious Diseases Association (KLİMİK), European Society for Clinical Microbiology and Infectious Diseases (ESCMID), Turkish Wound Care and Tissue Repair Association and International Diabetic Foot Working Group (IWGDF). He has been participating in the Diabetic Foot Infections Diagnosis and Treatment Guide studies prepared by the International Working Group of Diabetic Foot as an associate member since 2011.

**Figure:**
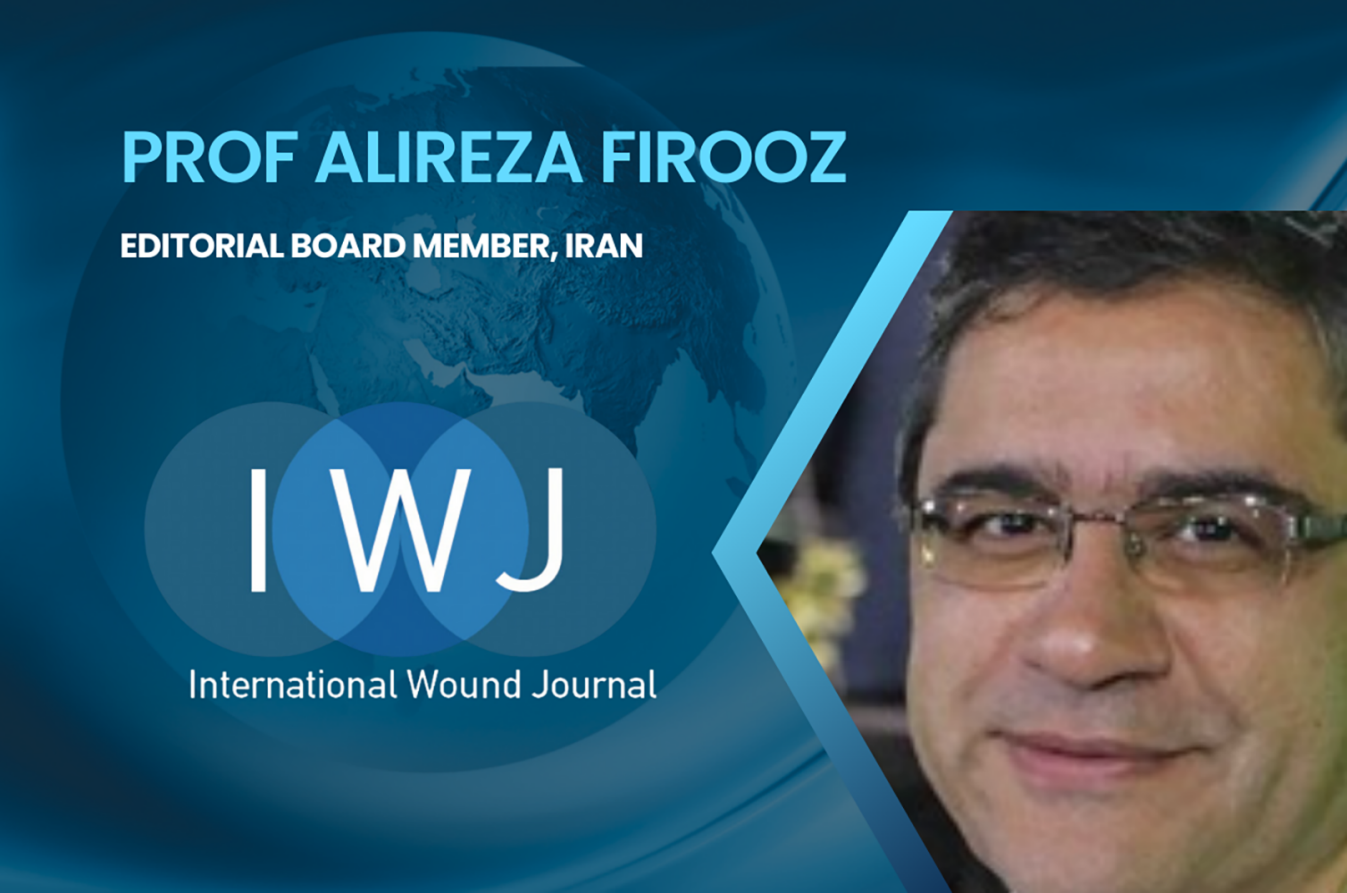
Professor Alireza Firooz, Iran

Professor Firooz is Professor of Dermatology, Center for Research & Training in Skin Diseases & Leprosy, Tehran University of Medical Sciences, since 2012 and is the Director, Center for Research and Training in Skin Diseases and Leprosy, Tehran University of Medical Sciences, Tehran, Iran, 2014 to Present.

His interests include evidence‐based dermatology, clinical trials, skin biometrology, cutaneous leishmaniasis, and dermatitis. With over 234 publications and gaining over 4085 citations, Prof. Firooz has an H‐index: 37.

**Figure:**
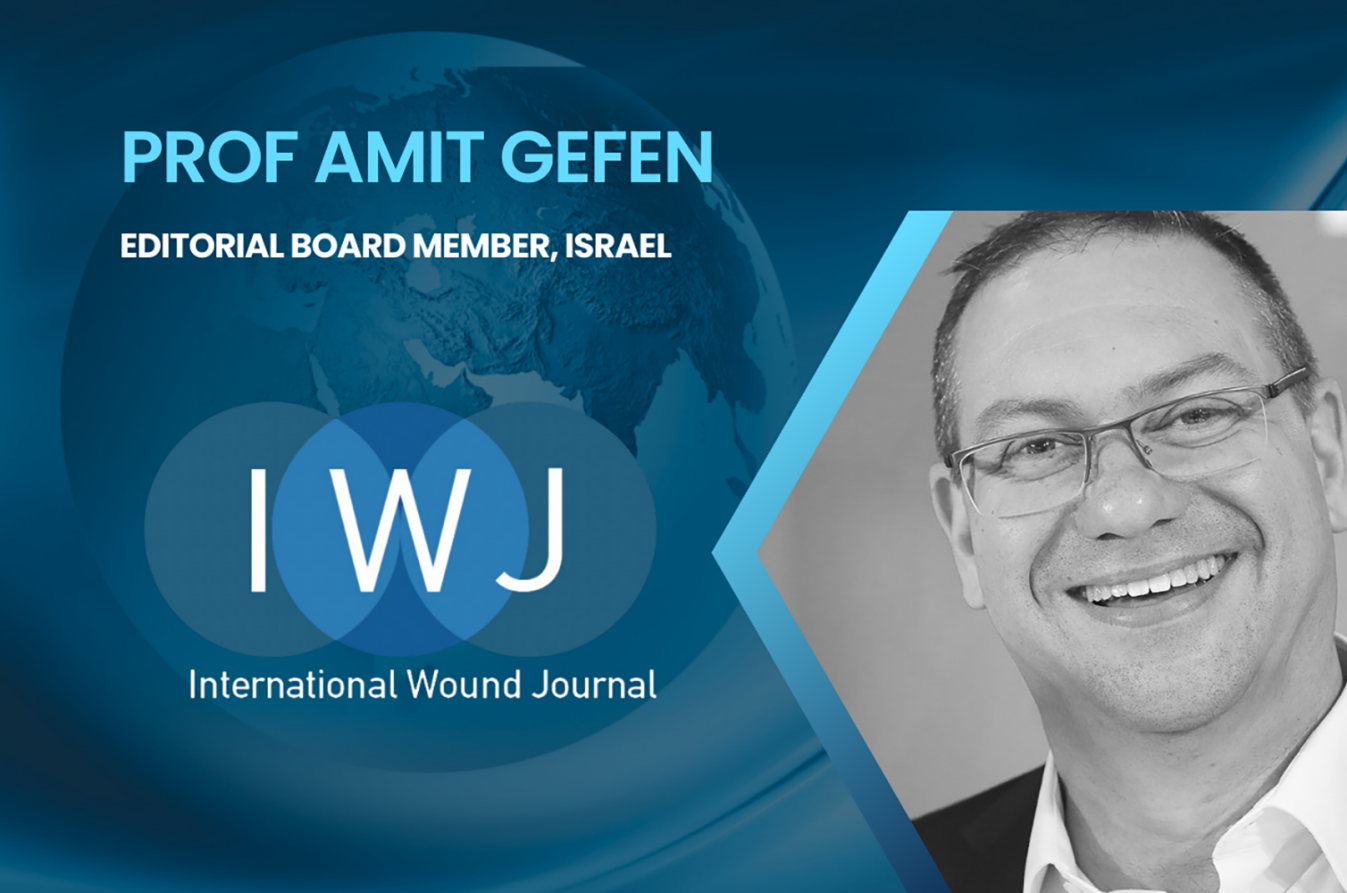
Professor Amit Gefen, Israel

Professor Amit Gefen received a BSc in Mechanical Engineering and MSc and PhD in Biomedical Engineering from Tel Aviv University in 1994, 1997 and 2001, respectively. During 2002–2003, he was a post‐doctoral fellow at the University of Pennsylvania, USA. He is currently a Full Professor with the Department of Biomedical Engineering at the Faculty of Engineering of Tel Aviv University and the Herbert J. Berman Chair in Vascular Bioengineering and also a Visiting Professor at the Faculty of Mechanical Engineering at the Technion—Israel Institute of Technology.

Professor Gefen is listed among the top‐ranked (i.e., most highly cited) 100 scientists in biomedical engineering worldwide at all times according to the recent global Stanford University & Elsevier ranking (2022). Prof. Gefen has also been the Head of the Ela Kodesz Institute for Medical Engineering & Physical Sciences at Tel Aviv University. The research interests of Prof. Gefen are in studying normal and pathological effects of biomechanical factors on the structure and function of cells, tissues and organs, with an emphasis on applications in wound research. In 2007–2008, he was a visiting scientist at Eindhoven University of Technology in the Netherlands where he developed tissue‐engineered model systems to study pressure ulcers. To date, Prof. Gefen published more than 320 articles in peer‐reviewed international journals, many of which were on mechanobiology, cell and tissue biomechanics, with applications that are mostly related to wounds and injuries. He is the Editor‐in‐Chief of Clinical Biomechanics (published by Elsevier) and has also edited several books (published by Springer, Elsevier and others) and several special issues in journals such as the *Annals of Biomedical Engineering, Journal of Biomechanics, Computer Methods in Biomechanics and Biomedical Engineering* and more. He is also editing a book series on *Mechanobiology, Tissue Engineering and Biomaterials* (published by Springer) and has served as an Associate Editor on the Editorial Boards of several international journals. Prof. Gefen was the President of the European Pressure Ulcer Society (2013–2015). He is the Vice‐Chair of the World Council of Biomechanics, a Fellow of the International Academy of Medical and Biological Engineering and the European Alliance for Medical and Biological Engineering and an Executive Board member of the International Society of Paediatric Wound Care.

**Figure:**
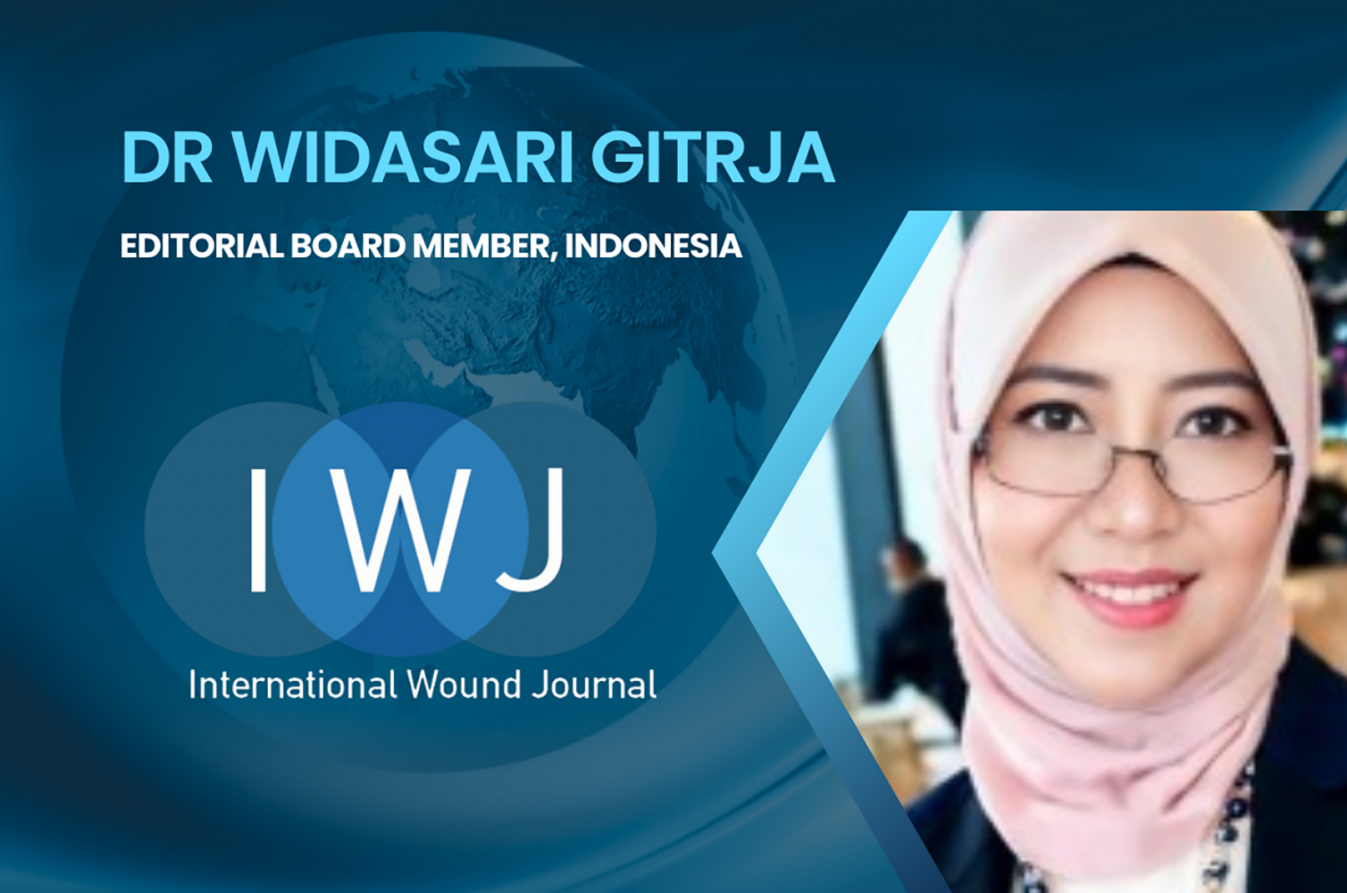
Dr. Widasari Gitrja, Indonesia

An experienced clinical nurse specialist with over 30 years of experience in wound, ostomy and continence care, Widasari is also a lecturer, researcher, author and entrepreneur. She is the founder and CEO of WOCARE Centre Indonesia, which pioneered Indonesia's wound care education and practice.

She also serves as the Program Director: Indonesian Enterostomal Therapy Nurse Education Program (INETNEP), recognized by WCET (2005 to present); a Clinical Nurse Specialist at three clinics: Wound Clinic, Dharmais Cancer Center Hospital (1993–2007); WOC Clinic, WOCARE Center (2007–Present).

**Figure:**
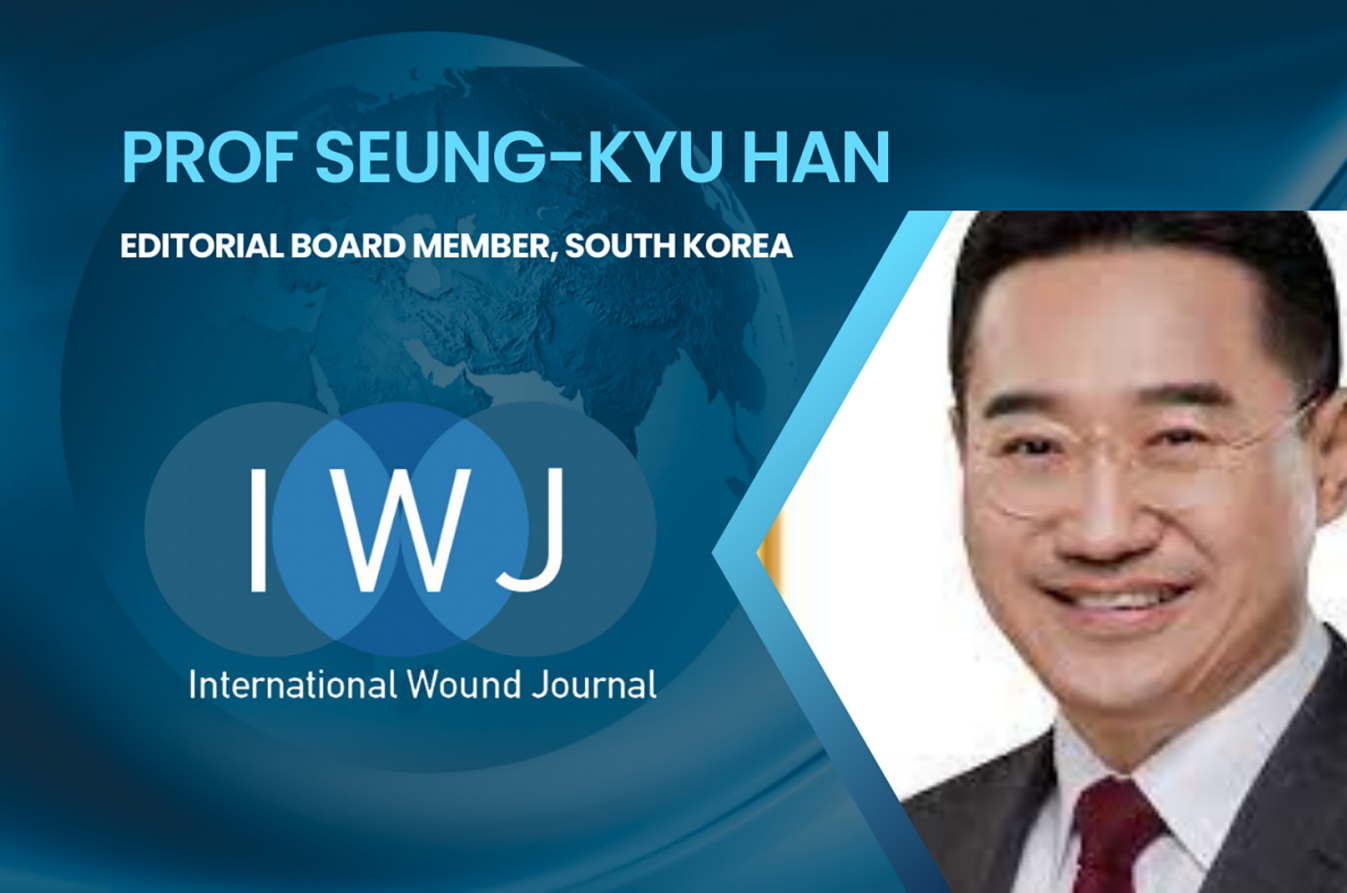
Professor Seung‐Kyu Han, South Korea

Dr. Seung‐Kyu Han, MD, PhD, is a tenured Professor of Plastic Surgery at Korea University College of Medicine. With a distinguished career spanning three decades at Korea University Plastic Surgery, Dr. Han has dedicated his efforts to pioneering innovative techniques and materials aimed at improving wound healing while minimizing invasiveness. Dr. Han's extensive contributions to the field include authoring five books, nine book chapters, and over 300 scientific articles, as well as editing six books. He also serves as a member of the editorial board of the *Aesthetic Surgery Journal*. He has previously held prestigious roles as President of the Korean Wound Management Society, the Korean Society for Diabetic Foot, the Korean Society for Aesthetic Plastic Surgery, and Korea University Guro Hospital.

**Figure:**
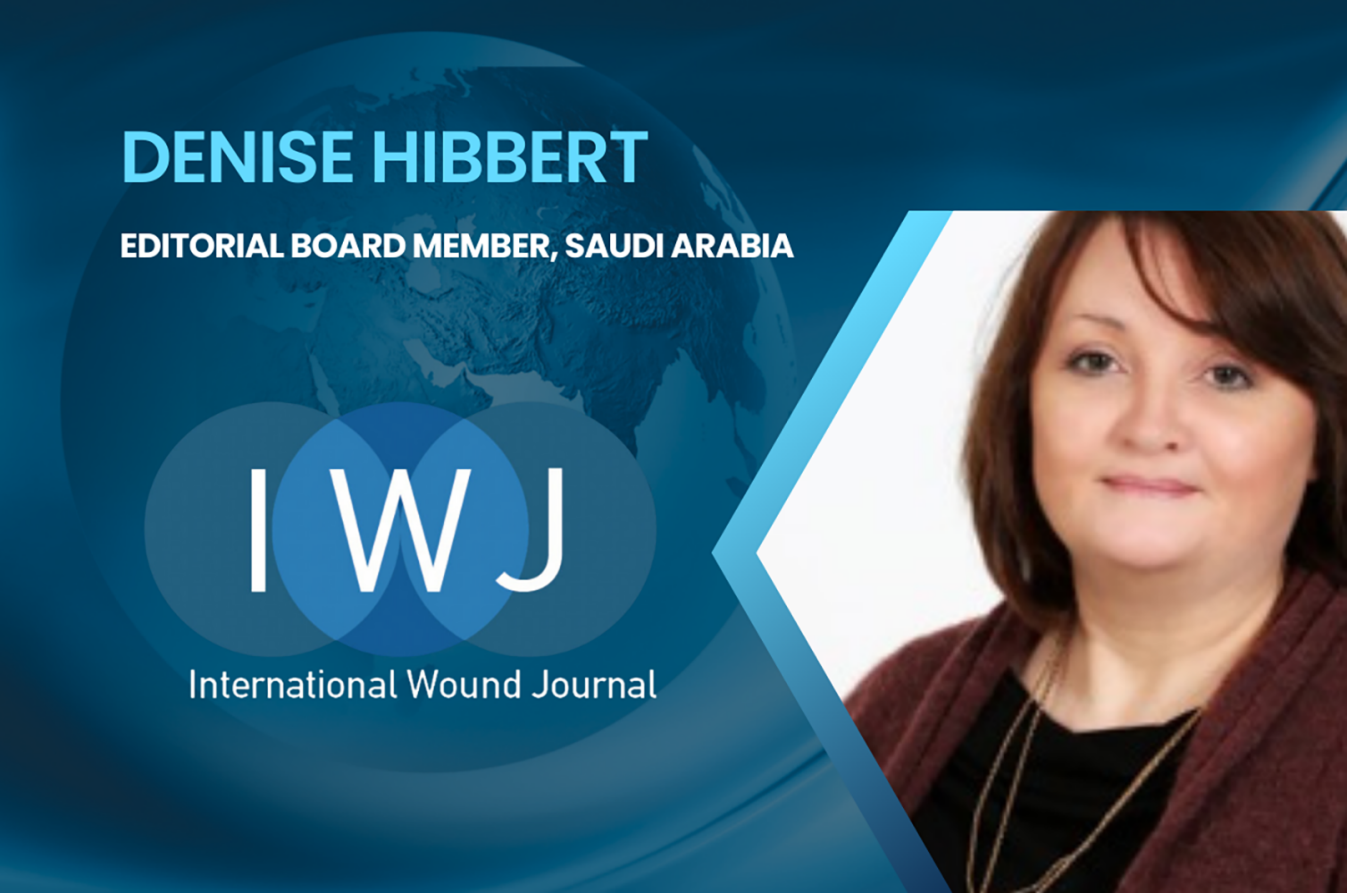
Denise Hibbert, Saudi Arabia

Senior Advisor to the Director General, Health Affairs of Princess Nourah bint Abdulrahmam University, Riyadh, Saudi Arabia. Registered Nurse and Senior Lecturer at Al Faisal University, Riyadh, Saudi Arabia.

President Elect of the World Council of Enterostomal Therapists® (WCET*) Executive Board Member of WCET*

Editorial Board member of *Annals of Saudi Medicine, World Journal of Colorectal Surgery, the World Council of Enterostomal Therapists Journal* and *Advances in Skin and Wound Care*.

Established Saudi Chapter of Enterostomal therapists in 2012.

Founder of a nationally and internationally recognized formal education program (diploma program) in enterostomal therapy (ET) in Saudi Arabia and initiated ET training into the curriculum of medical schools.

She has 35 years of experience in colorectal nursing with 25 years in stoma, wound, continence and anorectal physiology and widely regarded as bringing expertise and training in the care of stoma patients to Saudi Arabia and throughout the Middle East.

She introduced Anorectal Physiology testing and biofeedback therapy in Saudi Arabia, including training for both Colorectal Fellows and Enterostomal Therapists.

**Figure:**
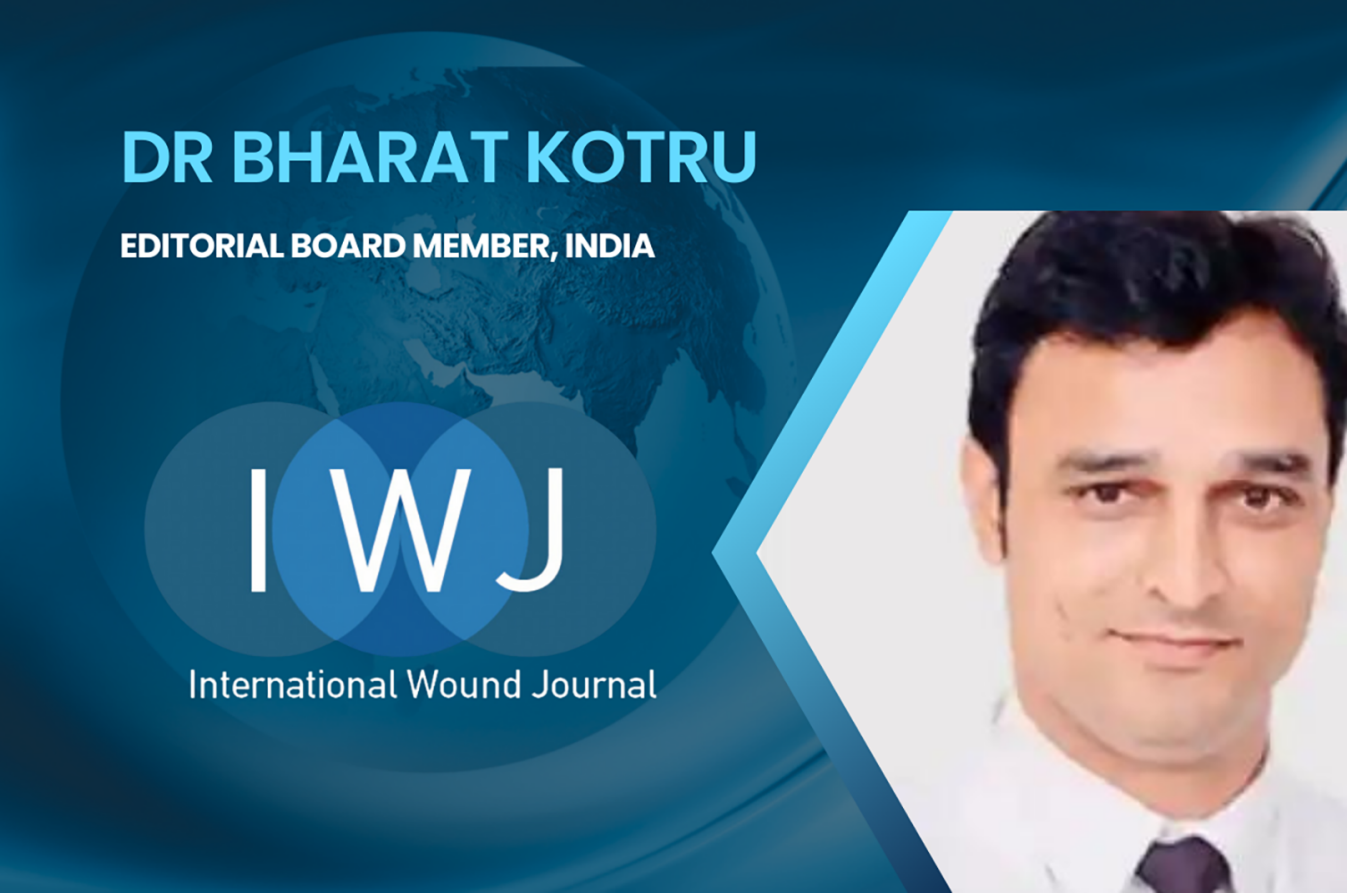
Dr. Bharat Kotru, India

Dr. Bharat Kotru is a distinguished podiatrist renowned for his expertise in foot and wound care. With a remarkable academic background, Dr. Kotru holds a PG in Advanced Clinical Podiatry from Spain, IIWCC certification from the University of Toronto, and an MSc in Clinical Skin Integrity and Wound Management from the University of Hertfordshire, UK. His specialized training also includes a credential from the Diabetic Foot Foundation at the Michener Institute.

Dr. Kotru's dedication to excellence is reflected in his commitment to ongoing education and professional development. He is a valued member of the Faculty of IIWCC at the University of Toronto, contributing to the advancement of podiatric care through research and education.

**Figure:**
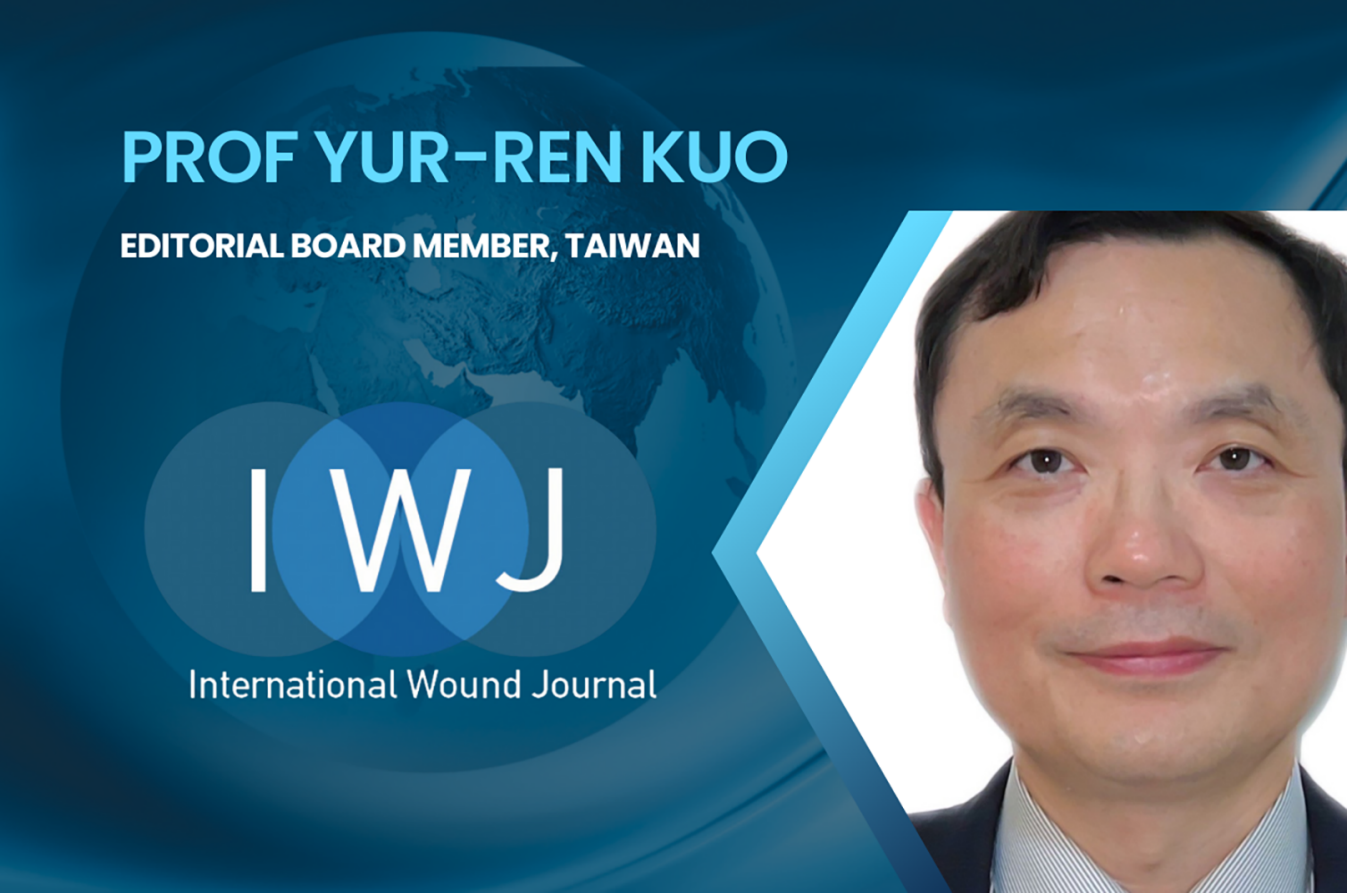
Professor Yur‐Ren Kuo, Taiwan

Prof. Kuo is currently the Professor and Chairman of the Department of Surgery, Director of Transplantation Center, Kaohsiung Medical University Hospital, Taiwan.

Dr. Kuo is an internationally renowned ‘Surgeon‐Scientist’, not only in clinical practice but also in basic research for more than 20 years. Dr. Kuo got a PhD degree from the Graduate Institute of Clinical Medicine, Chang Gung Medical College in 2003. Then, he served as the Post‐Doctor Research Associate position in the University of Pittsburgh Medical Center, PA, between 2004 and 2005. His research focused on the immunomodulation of composite tissue allotransplantation. He established the first heterotopic large animal (swine) limb transplant model (2005) in Taiwan and the first orthotopic large animal (Swine) hemi‐facial transplant in the world (2008) to study the mechanisms of tolerance induction and chimerism.

Around 10 years' preparation included animal studies and pre‐clinical cadaveric mock surgery; he got the Institutional Review Board (IRB) approval and started the hand allotransplant program. His team performed the first‐hand allotransplantation in Taiwan on Sep. 3, 2014. His achievement let Taiwan be listed as one of the famous institutes of composite tissue allotransplantation in the world.

Prof. Kuo has been honoured with numerous awards, orations, professorship and honorary appointments. He has been selected as ‘International Guest Scholar’ awarded by the American College of Surgeons (ACS) in 2003. He is the first surgeon trained in Taiwan to get this honour since past 40 years of funding the awards.

Prof. Kuo is an expert focused on microsurgical reconstruction for head neck and extremity defects. Prof. Kuo has mentored 40 clinical fellows over the past 20 years. He is also a true physician‐scientist devoted in transplant immunology, reconstructive microsurgery, wound healing, keloid scars and stem cell regenerative medicine. He has authored more than 200 leading peer‐reviewed innovative clinical and numerous basic research papers, six book chapters, and served as an Editorial Board committee member of five scientific journals and as a reviewer for more than 40 journals. He has been recognized as the World's Top 2% Scientists Annual Scientific Impact Rankings for 2020–2022 released by Stanford University.

**Figure:**
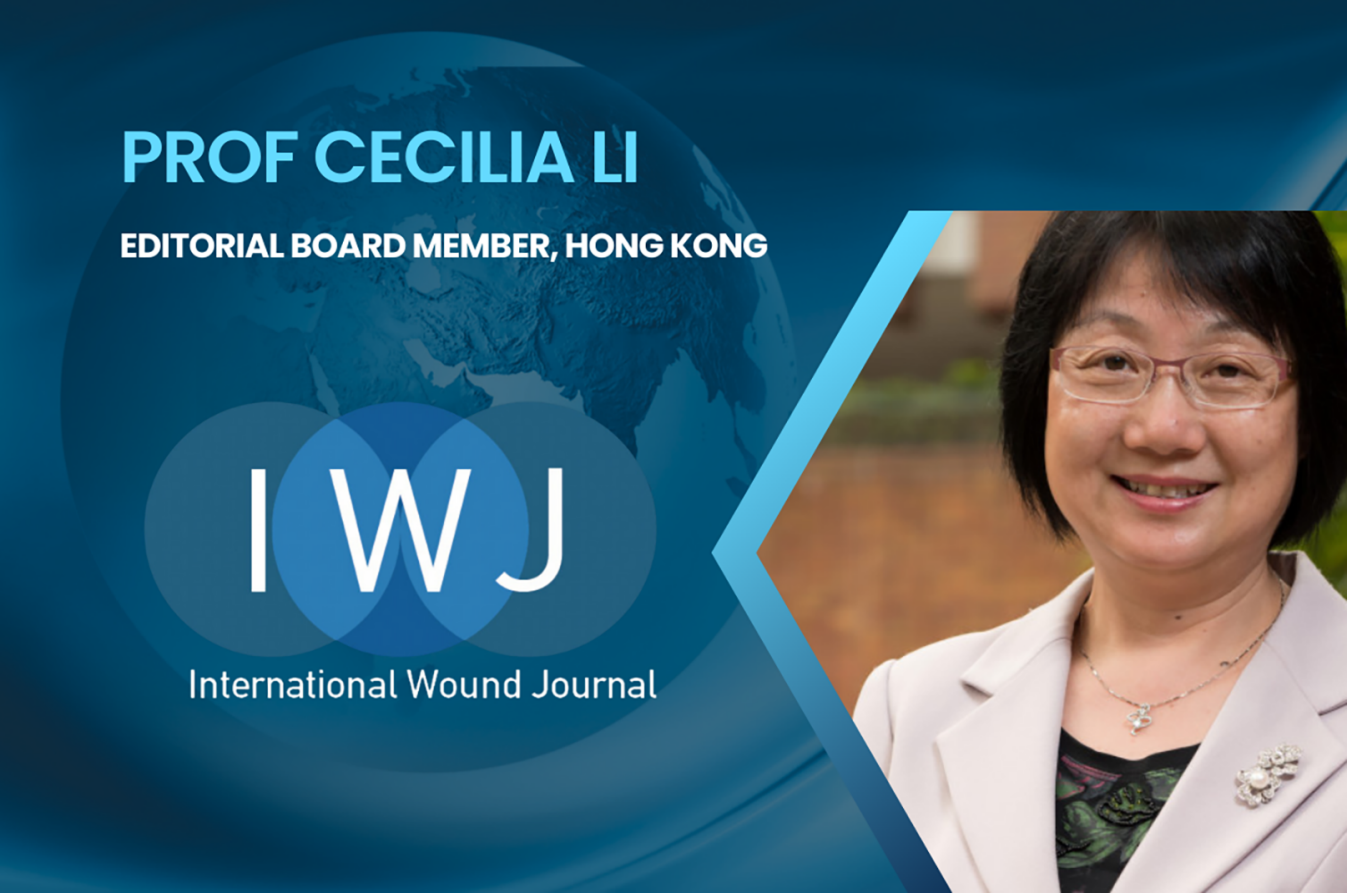
Professor Cecilia Li, Hong Kong

Prof. Cecilia Li‐Tsang was the Associate Head and Professor at the Department of Rehabilitation Sciences at the Hong Kong Polytechnic University ever since 1989. She is currently the visiting professor at the Faculty of Health Sciences at the university upon her retirement recently. She obtained her PhD at the Faculty of Medicine, The Chinese University of Hong Kong. She has more than 30 years of academic teaching and research experiences in the field of physical rehabilitation. She is specialized in the field of burns, wound and scar management, paediatrics and hand and work‐related injuries.

Her research work in burns and scar management was awarded the State Scientific and Technological Award, China (Second Class Award) in 2012. Her inventions, the Smart Pressure Monitored Suit (SPMS) and the Smart Scar Care Pad (SSCP) were both awarded Gold Medal with the Congratulation of Jury at the International Exhibition of Inventions of Geneva (2010 and 2017), and Innovation Award from the National Council of Rector from Romania in 2017.

She has published more than 100+ journal articles, seven books as authors/co‐authors, 20+ book chapters, 120+ conference papers and numerous news release to the media. She is the senior editor of the *Journal of Burn and Trauma*, associate editor of the *Hong Kong Journal of Occupational Therapy* and *WORK* journal. She is also a member of the editorial board for *Journal of Hand Therapy, British Journal of Occupational Therapy, Burn and Trauma*. She also serves as a reviewer for a number of internationally renowned journals, namely, *BURNS*, *Journal of Burn Care and Research*, *Journal of Physical Medicine and Rehabilitation*, *Scientific Reports*, *PLOS One*, *Research in Developmental Disabilities*, *Journal of Intellectual Disabilities*, and *Journal of Plastic and Reconstructive Surgery*. She also holds positions as the Chairman, Board of Directors, Wai Ji Christian Services; Executive Committee member, Hong Kong Rheumatology and Arthritis Foundation; Consultant, HK Christian services; Honorary Advisor, SAHK; and consultant, IDEAL (parents organization).

**Figure:**
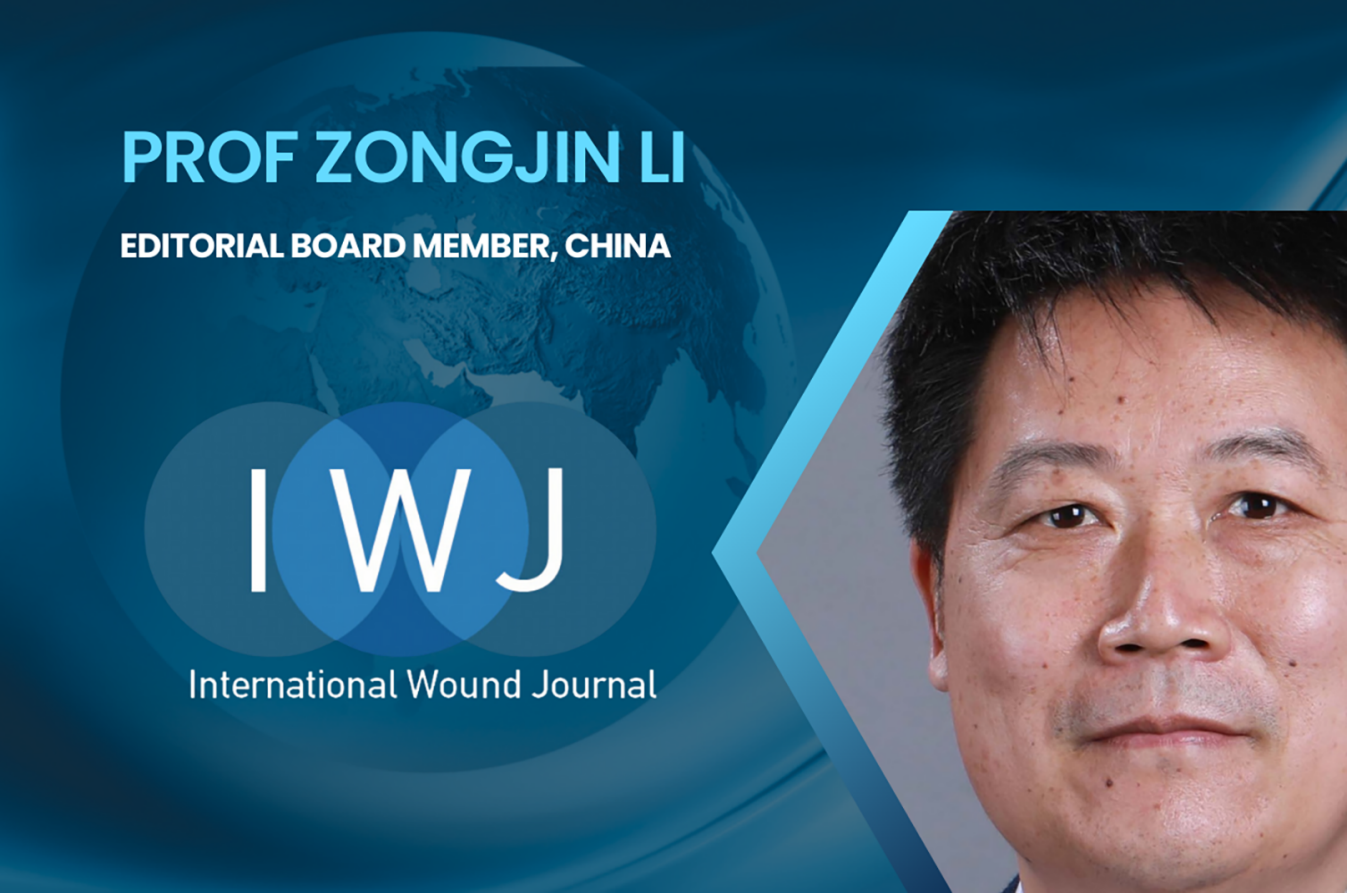
Professor Zongjin Li, China

Dr. Zongjin Li is a Professor at the Department of Pathophysiology and Director of the Laboratory of Molecular Imaging and Stem Cell Therapy at Nankai University School of Medicine, Tianjin, China. He received his PhD degree from Peking Union Medical College and completed his postdoctoral training at the Molecular Imaging Program at Stanford (MIPS) and the Department of Radiology at Stanford University (USA). Dr. Li has published more than 150 manuscripts with an H‐index of 49. Dr. Li conducts extensive research, focusing on stem cell therapy, extracellular vesicles (EVs), and molecular imaging. The goals of his research are to enhance our understanding of tissue regeneration and repair, accelerate the discovery of new therapeutics, and propose innovative strategies for sequential regulation and intervention in the treatment of tissue injuries, aging, and degenerative diseases. His work has appeared in peer‐reviewed journals, including *Science Advance, Advance Science, Circulation, Journal of the American College of Cardiology, Journal of the American Society of Nephrology, Nature Methods, Biomaterials*, and many more. Dr. Li is Vice Director of the Chinese Society of Biomedical Engineering, Council on Stem Cell Engineering, and International Fellow of the American Heart Association (FAHA).

**Figure:**
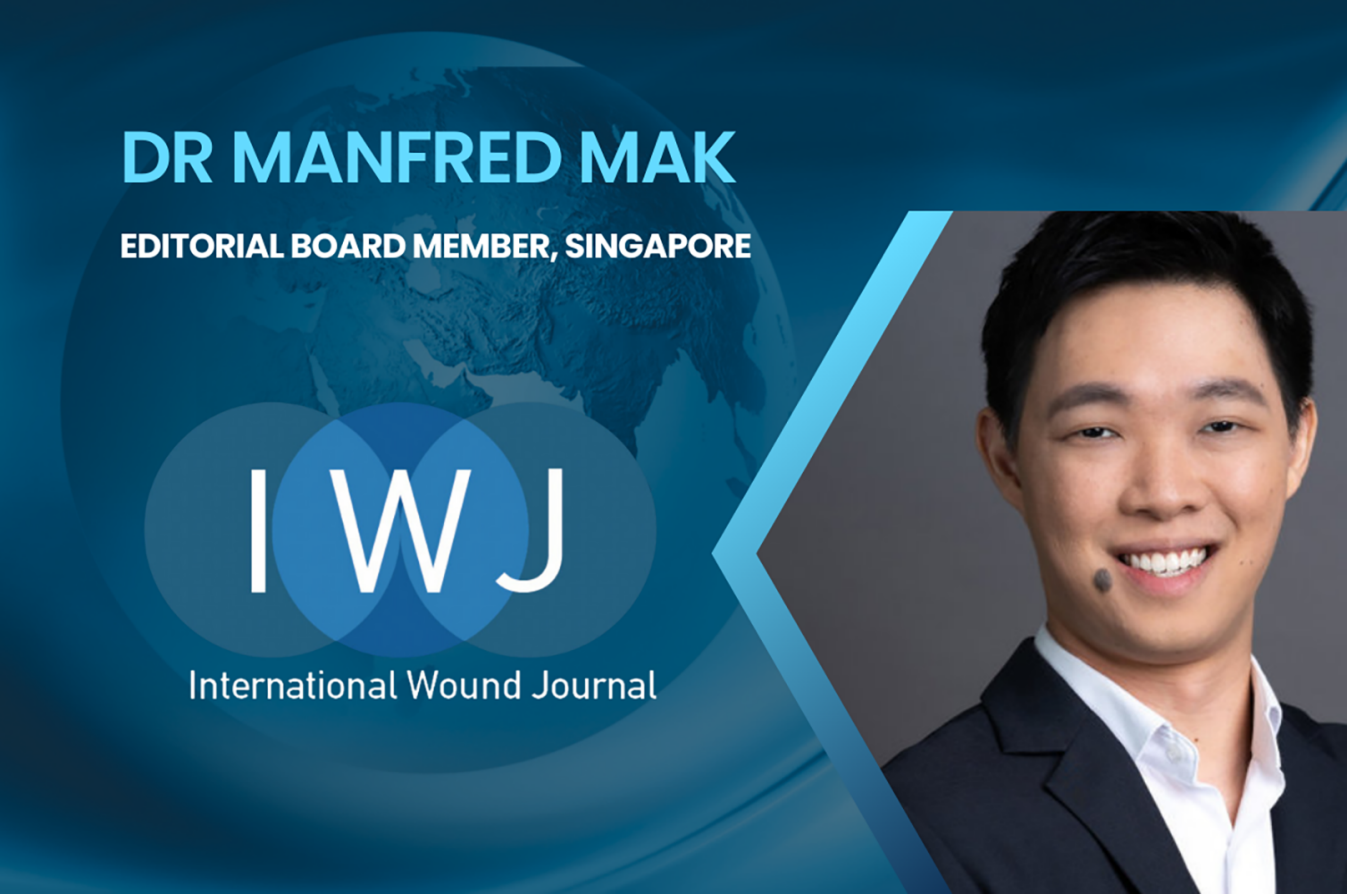
Dr. Manfred Mak, Singapore

Mr. Manfred Mak is a Senior Principal Podiatrist at Singapore General Hospital. He previously held the position of Head, Podiatry, at Singapore General Hospital for 5 years before stepping down. Manfred was awarded the Singapore General Hospital (SGH) Overseas Scholarship in 2006, graduated with First Class Honours in Podiatry from the University of Southampton and holds a Master's degree in Public Health from the National University of Singapore. He was also awarded a Fellow of Faculty of Podiatric Medicine from the Royal College of Surgeons and Physicians, Glasgow.

Manfred completed his Health Manpower Development Plan in 2013, with Professor David G. Armstrong, a renowned Podiatric Surgeon, at the Southern Arizona Limb Salvage Alliance, University of Arizona Medical Centre, USA. He is active in research and his area of interest is in preventing and managing diabetic foot complications and limb salvage.

**Figure:**
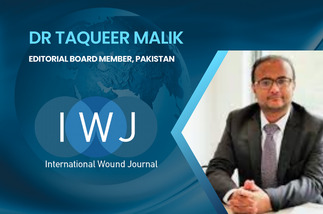
Dr. Taqueer Malik, Pakistan

Dr. Tauqeer Ahmed Malik, MBBS (Pakistan), RMP (Pakistan), BSc (Pakistan), MRCS (RCSEd‐UK), CCBST (UK‐Ireland), PgDip WH & TR (Cardiff University‐UK). He is working as a diabetic foot consultant surgeon and interventionist in Islamabad, Pakistan, and as an Associate Consultant diabetic foot and wound care surgeon at King Fahad Armed Forces Jeddah in Saudi Arabia. Taqueer is a visiting wound care surgeon for the royal family of KSA.

Professor Malik played a major role in the research on the role of human amniotic membrane in the management of acute and chronic wounds. This research was the first of its kind in Saudi Arabia and the Middle East. He has six scientific papers and publications published in various international journals.

He has conducted many wound care workshops, trainings and in‐service lectures on diabetic foot and wound care in various departments and is often an invited speaker at various local, regional and international diabetic foot and wound care conferences. His vision is to enhance diabetic foot and wound care in Pakistan: Thus, reducing amputation, pressure injuries and surgical site infection rates to a minimum.

He wishes to create a centre of excellence within Pakistan where it will host CME‐accredited diabetic foot and wound care workshops, seminars and international conferences at regular intervals. In collaboration with local and national, both private and governmental health authorities this will extend best‐practice diabetic foot and wound care services. These services will include pressure injury prevention programs, diabetic foot prevention programs, training wound care champions and hands‐on workshops on products and techniques.

The centre will be conducting new and innovative research with regards to diabetic foot and wound care that would include human amniotic membrane, platelet‐rich plasma and new dressing material.

**Figure:**
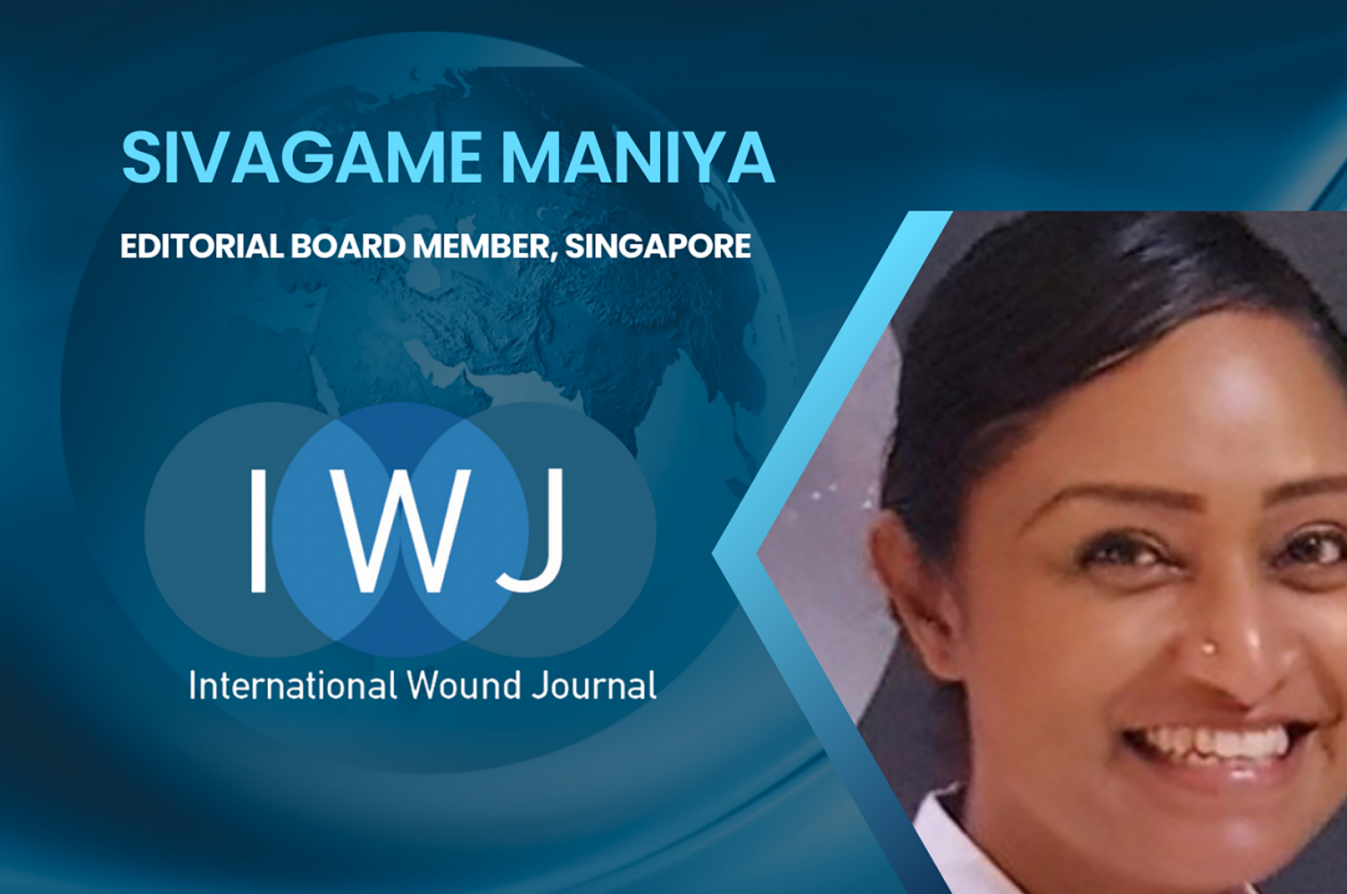
Sivagame Maniya, Singapore

Ms Sivagame Maniya is an Advanced Practice Nurse in a vascular specialty for 23 years and heads a nurse‐led leg ulcer clinic and a wound care team in a tertiary hospital. She received her Doctorate in Nursing from Duke School of Nursing, USA. She attained her International Interdisciplinary Wound Care Course Accreditation from the University of Toronto and Post‐Graduate certificate in Wound, Ostomy and Continence Nursing from Curtin University. She is the current Vice‐President of the Wound Healing Society (Singapore) and a member of the World Council of Enterostomal Therapists (WCET) and the European Wound Management Association. Ms. Siva has a keen interest in leg ulcers and maggot therapy. She is currently in the Guideline Development Group Venous Leg Ulcer Advisory Panel committee for the Australian and NZ Wound Management Association and was in Small Working Group member for International Pressure Injury Guideline (2019).

**Figure:**
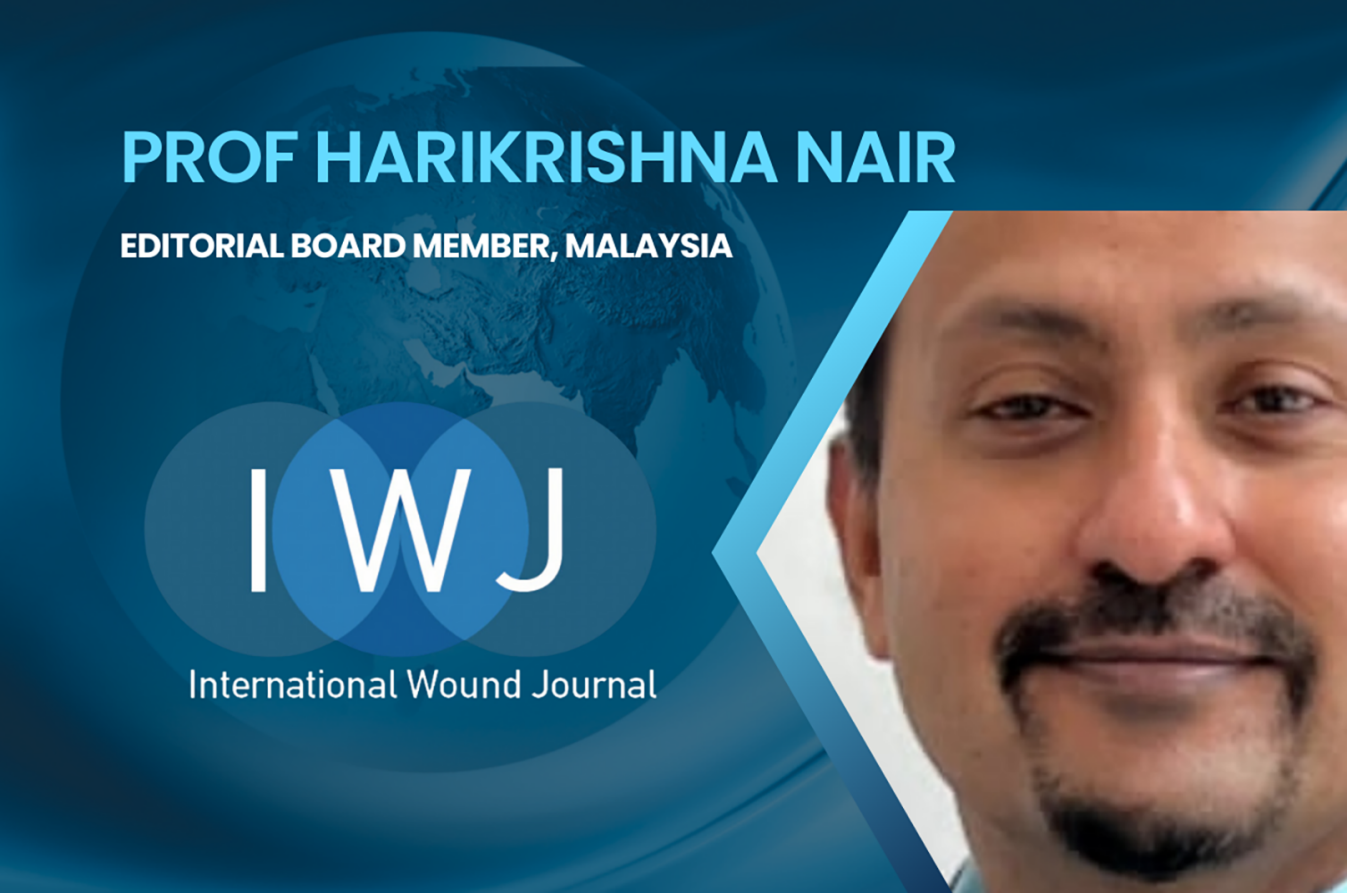
Professor Harikrishna Nair, Malaysia

Dr. Harikrishna Nair is the Chairman of the Association of Southeast Asian Nations (ASEAN) Wound Council and President of the Malaysian Society of Wound Care Professionals (MSWCP). He holds the position of Recorder and Executive Board Member at the World Union of Wound Healing Societies, and he is the Vice President of the Asian Wound Care Association. Currently, he leads the Wound Care Department at Kuala Lumpur General Hospital, Malaysia. He is the National Advisor for the Primary Health Ministry of Health Malaysia and a Professor of Wound Care at the Faculty of Medicine, Lincoln University.

Besides being a prolific speaker and trainer in both local and international arenas, he has also published many international wound care‐related studies and is the Editor in Chief of *Wounds Asia Journal* and the *Journal of Wound Care Silk Road Supplement*. He is a faculty member of NADI and Chairman DCOM; Professor of The Institute of Health Management, Austria; Chairman of Pressure Injury Prevention Committee; KLH and a member of the National Technical Committee on Wound Care MOH. He is also an Adjunct Professor, Department of Surgery, IMS, BHU, INDIA, and The Executive Director of The College of Wound Care Specialists since 2017. He is the Course Director of The Certificate in Clinical Wound Care (CCWC) since 2017, which is the first certification program started in Malaysia.

**Figure:**
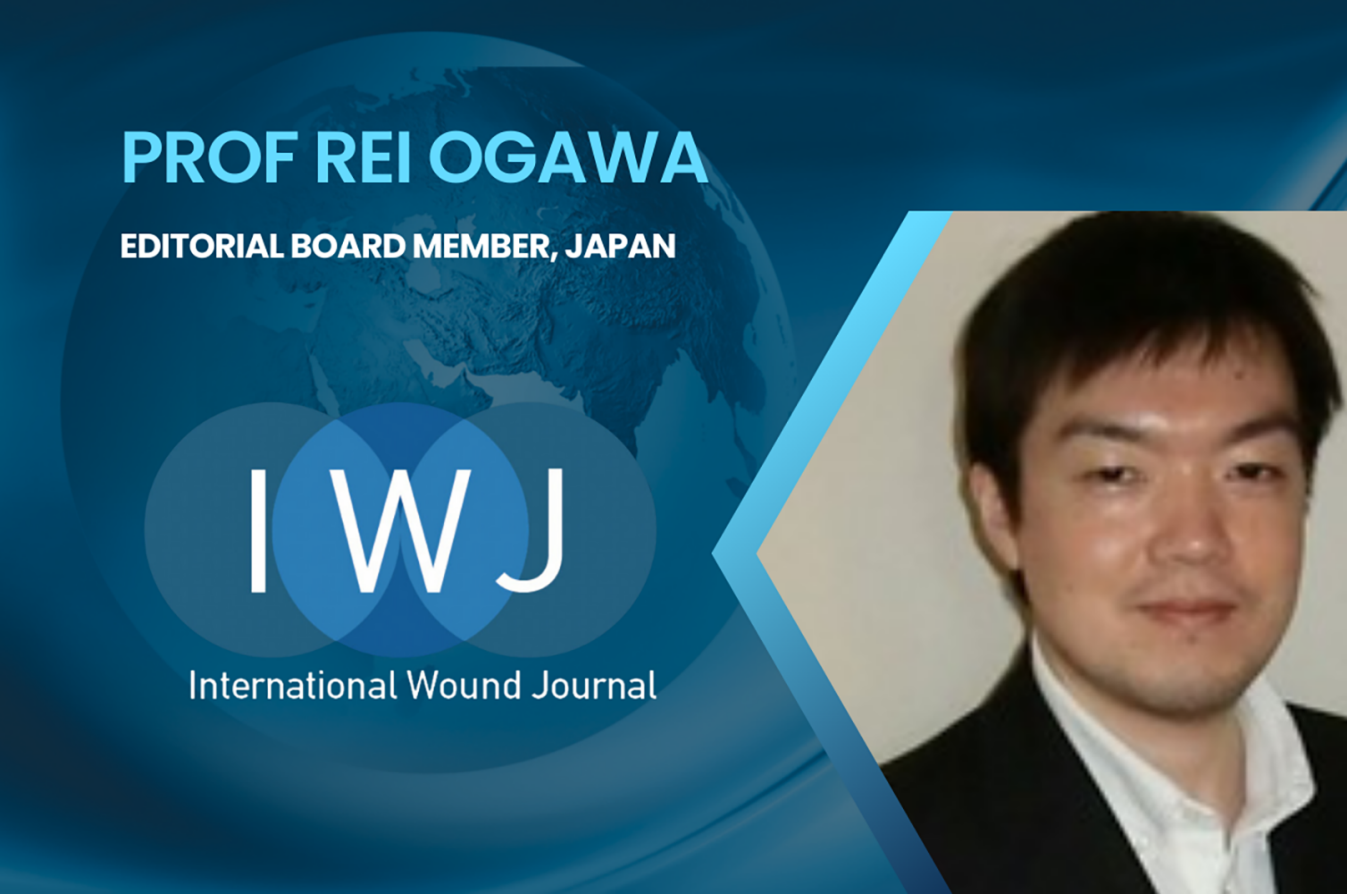
Professor Rei Ogawa, Japan

Rei Ogawa has the M.D. and PhD degrees. He is currently a faculty member at the Nippon Medical School in Tokyo, Japan, with a position of Professor and Chief at the Department of Plastic, Reconstructive, and Aesthetic Surgery. Also, he is a Vice Dean of the medical faculty of Nippon Medical School.

He is a fellow of the American College of Surgeons (ACS) and a member of the American Association of Plastic Surgeons (AAPS). In addition, he is now directing the Mechanobiology and Mechanotherapy Laboratory at his medical school. He joined the Tissue Engineering and Wound Healing Laboratory, Brigham, and Women's Hospital, Harvard Medical School, Boston, USA, where he worked between 2007 and 2009 as a Research Fellow. He has focused his recent studies on mechanobiology and its application to tissue engineering, wound healing and anti‐aging medicine. His clinical specialty is reconstructive surgery and scar management, for example, abnormal scar (keloid and hypertrophic scars) prevention and treatment. In relation to this, he studied mechanobiology of wound healing and scarring, and he is a world leader in this area. He is now the president of the Global Scar Society (G‐ScarS) and the Japan Scar Workshop (JSW).

He was the recipient of several awards, for example, the Award of Japanese Society of Plastic Surgery, and many research grants (e.g., grant‐in‐aid for scientific research in Japan). Moreover, he holds several national patents in the field of tissue engineering and mechanobiology. He is an Editorial Board Member of many international/local scientific journals (e.g., *Plastic and Reconstructive Surgery*) and is a Board Member of international/local medical societies (e.g., *Japanese Society of Plastic and Reconstructive Surgery* [JSPRS]).

He has coauthored over 50 chapters in international/national books, coauthored over 700 papers in international/national scientific journals, and has presented over 2500 coauthored papers at international/national conferences, including over 400 invited lectures.

**Figure:**
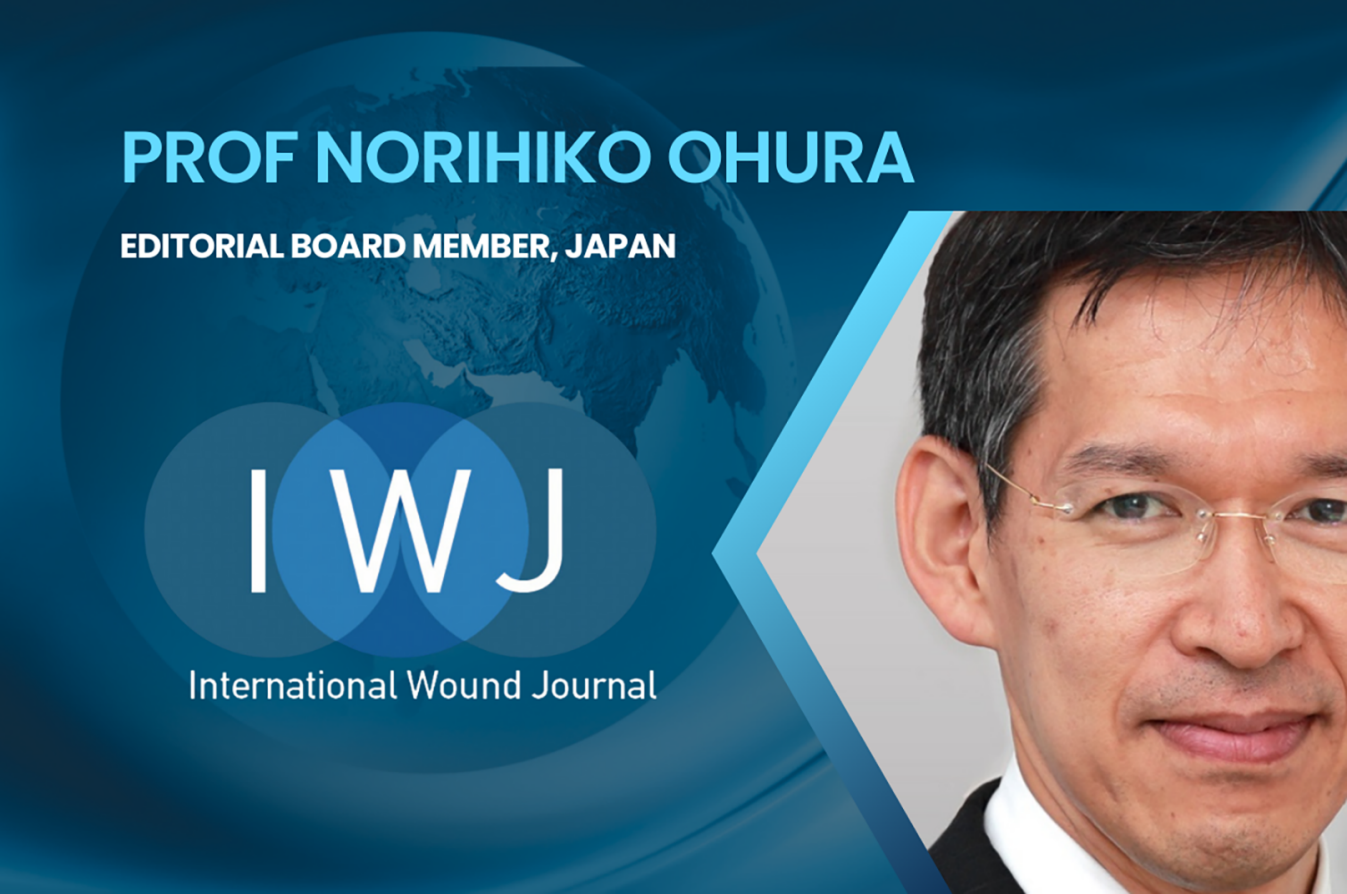
Professor Norihiko Ohura, Japan

Norihiko Ohura is a professor in the Department of Plastic and Reconstructive Surgery at Kyorin University School of Medicine in Tokyo. He also serves as a professor at the Graduate School of Information Science and Technology, Smart Contract Applied Lab in Osaka. He earned his PhD in 2001 from the Department of Biomedical Engineering at the University of Tokyo Graduate School of Medicine. In 2019, he was awarded the EPUAP2019 Best Poster Presentation Award and chaired the Next Generation Medical Devices/Regenerative Medicine Products Evaluation Indicator Project for hard‐to‐heal wound treatment devices. He is devoted to research in pressure ulcer prevention and wound assessment by using AI. In his clinical research, he has partnered with cardiologists to conduct numerous CLTI registry studies.

**Figure:**
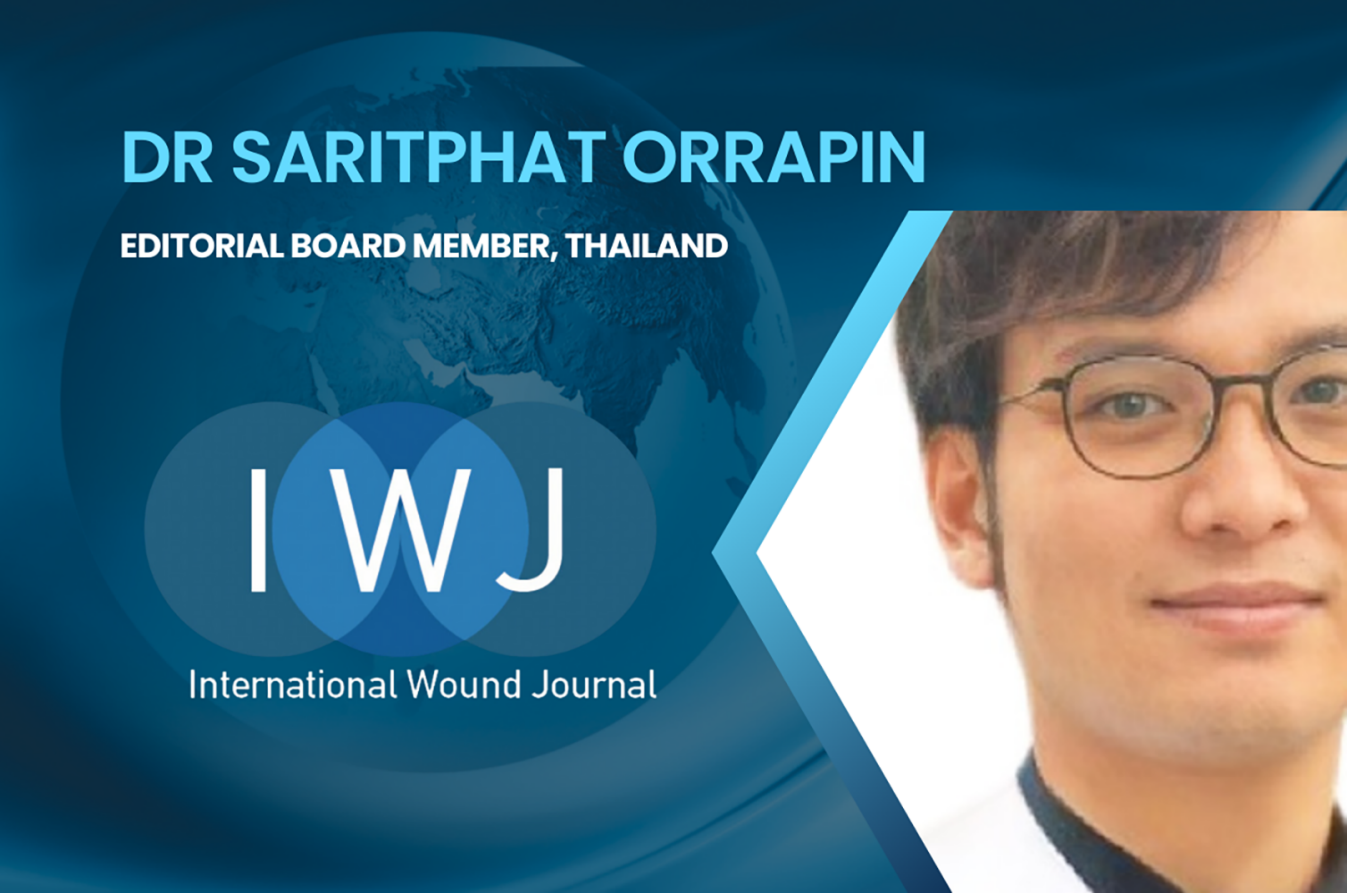
Dr. Saritphat Orrapin, Thailand

Dr. Saritphat Orrapin is a vascular surgeon with expertise in treating a broad range of conditions, including chronic limb‐threatening ischemia (CLTI), diabetic foot ulcer (DFU), peripheral vascular disease, chronic venous disorder, venous ulcer and other chronic leg ulcer (CLU). Orrapin's participation in vascular disease and chronic leg ulcer research over the past 10 years has included trials to evaluate the result of endovascular and surgical revascularization in DFU and CLTI, angiosome and woundsome concepts. He also participates in the biological therapy and advance wound care involving growth factors, oxygen therapy and other novel technology in DFU and CLU.

He has served as principal investigator for numerous international original articles and meta‐analysis in vascular disease. His experience also includes providing consultantation for limb revascularization procedures and DFU treatment in CLTI patients for surgeons and angiologists. Orrapin earned his medical degree and completed a residency in general surgery and a fellowship in vascular surgery at the Chiang Mai University (CMU) School of Medicine. He has been working as a Medical Instructor and Consultant in the Division of Vascular Surgery, Faculty of Medicine, Thammasat University, since 2017.

Orrapin has served as The Council and Board Members of Asia Pacific Association for Diabetic Limb Problems (APADLP) and Deputy Director of the Center of Excellence for Diabetic Foot Care at Thammasat University Hospital. He also served in the committees of Thai Vascular Association, Wound Healing Association (Thailand), Diabetic Foot Club (Thailand) and Royal College of Surgeons of Thailand (RCST). He is a fellowship of the international College of Surgeons (FICS), Thailand section.

He is in the Editorial Board of the *International Journal of Lower Extremity Wounds, SAGE journals, the Thai Journal of Burn and Wound Healing*, and *the Thai Journal of Surgery*. Orrapin has been a reviewer and an editor of several articles, including the *International Journal of Lower Extremity Wounds: Healing following revascularization*—unlocking skin potential (Issue March 2024), as well as several articles in peer‐reviewed journals. He finished a diploma and PhD in Clinical Epidemiology and Statistics in 2024.

**Figure:**
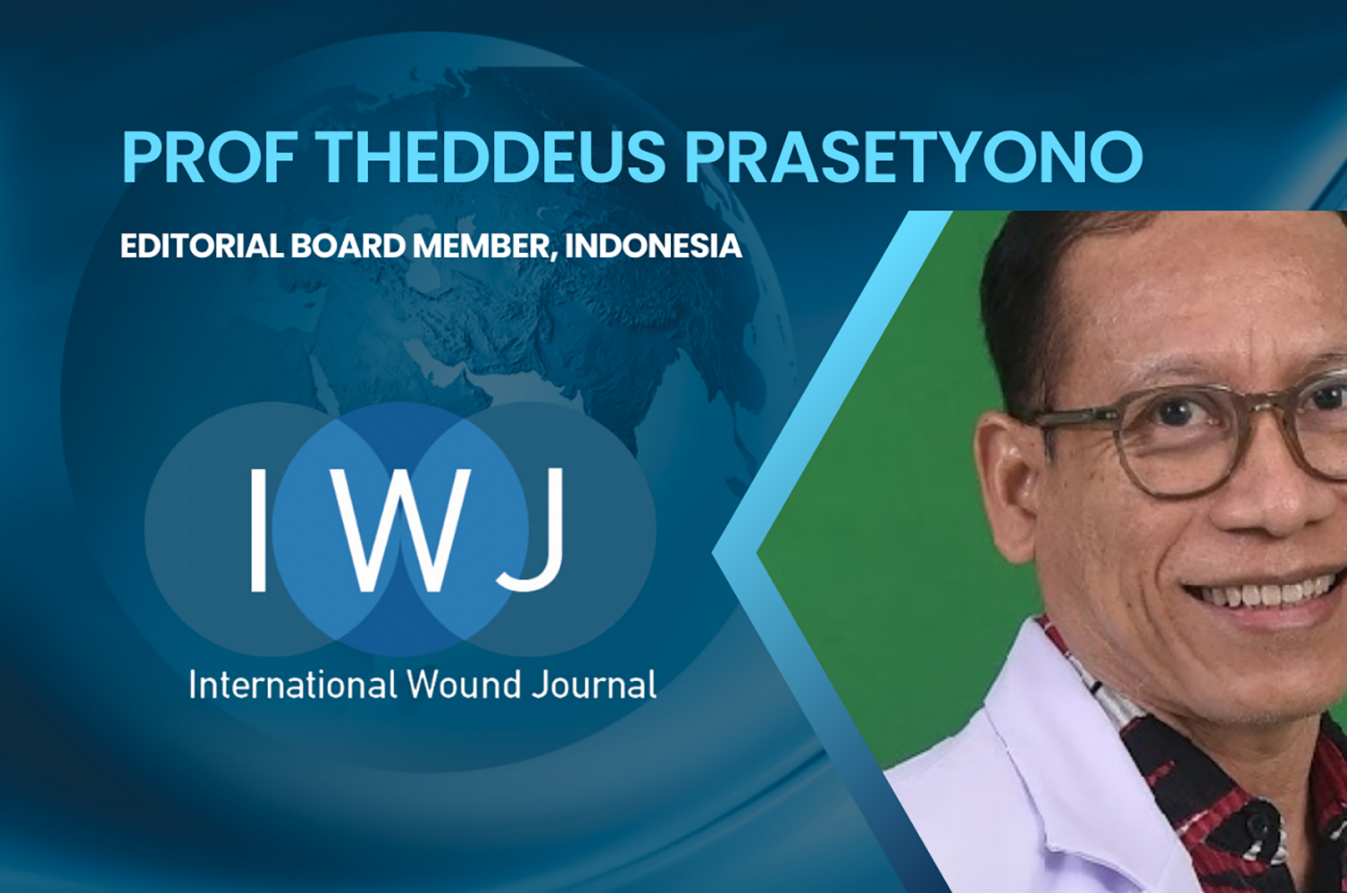
Professor Theddeus Prasetyono, Indonesia

Prof. Prasetyono is a hand and microsurgery consultant in plastic surgery, Cipto Mangunkusumo Hospital/Universitas Indonesia in Jakarta, Indonesia. He is also the Chairman of the Indonesian Clinical Training and Education Center (ICTEC) of the same hospital and university. He actively participates in various seminars, training and scientific meetings, as a speaker and participant. He has long years of experience in organizing international events as well. Besides publishing papers in journals and books, Dr. Prasetyono is also an editor and a reviewer of several international journals. He has visited some high‐profile centers as a visiting professor/surgeon in many countries, while also experienced as an external examiner for a plastic surgery program in Malaysia.

**Figure:**
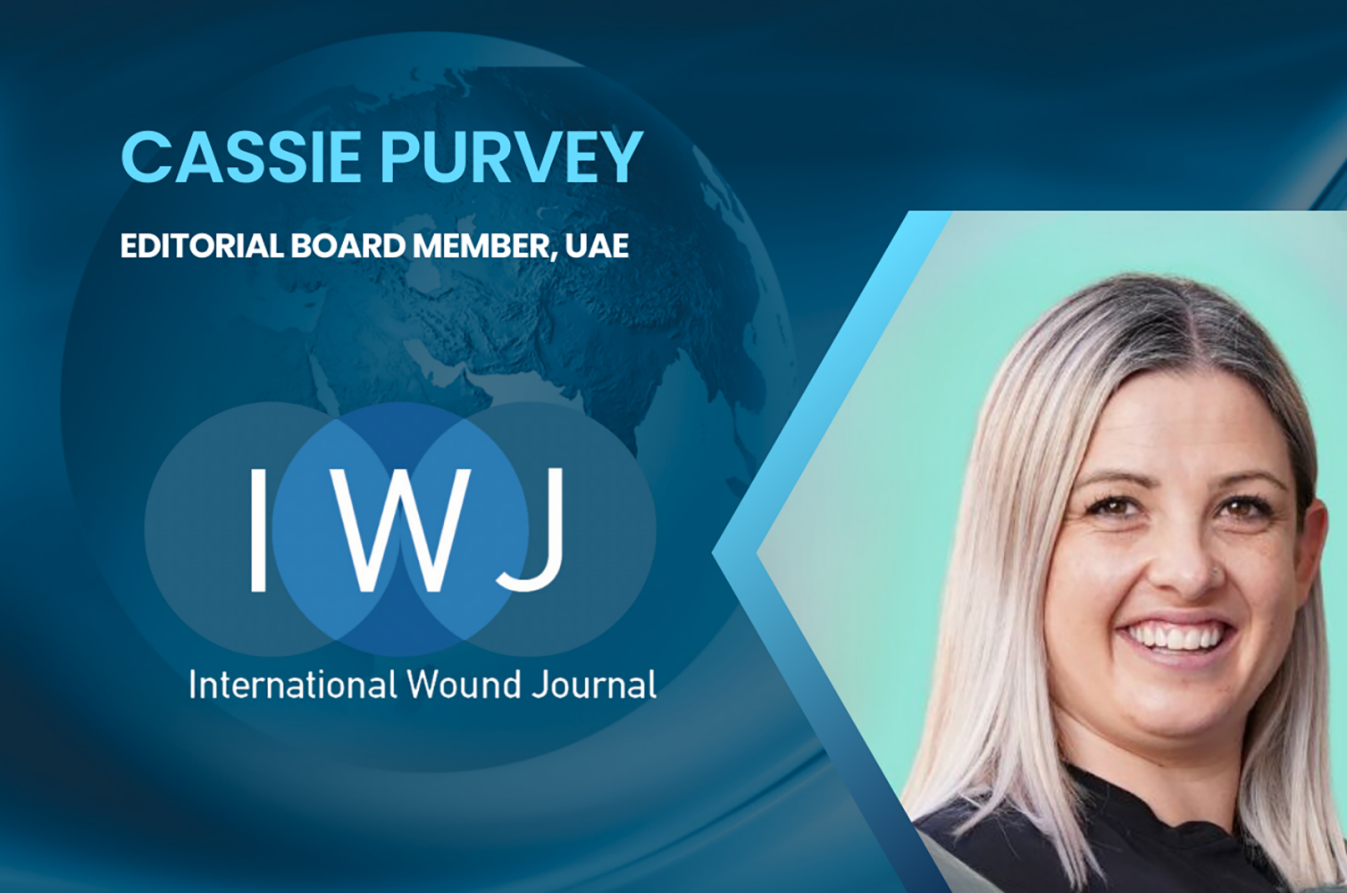
Cassie Purvey, UAE

Bringing over 22 years' experience as an Advanced Wound and Stoma Care specialist, with clinical experience across Australia, UK and the UAE, Cassie is a Senior Burns Specialist Nurse from Australia and previously a member of the Royal Adelaide Hospital Burns Assessment Team. In the UAE, she has successfully developed the region's largest nurse‐led wound and stoma care service which has received many accolades, including the World Union of Wound Healing UAE Local Wound Clinical Wound Centre of Excellence. Cassie has also achieved many awards during her career, including the *Journal of Wound Care* Bronze Award for Excellence in Wound Care from the UAE and Dubai ‘Champion Nurse Award’. Cassie is Chair of the Stoma Care Program for the Emirates Society of Colon and Rectal Surgery and a member of the EPUAP Pressure Injury Expert Panel Group.

QUALIFICATIONS:

Masters of Nursing Science (University of Adelaide). Thesis: Patient Experience of Chronic Wounds in a Tertiary Healthcare Setting.
Post Graduate Diploma Nursing Science—Burns Nursing (University of Adelaide) Bachelor of Nursing (University of South Australia).
Post Graduate Certification Advanced Stoma Care (University of East Anglia).

**Figure:**
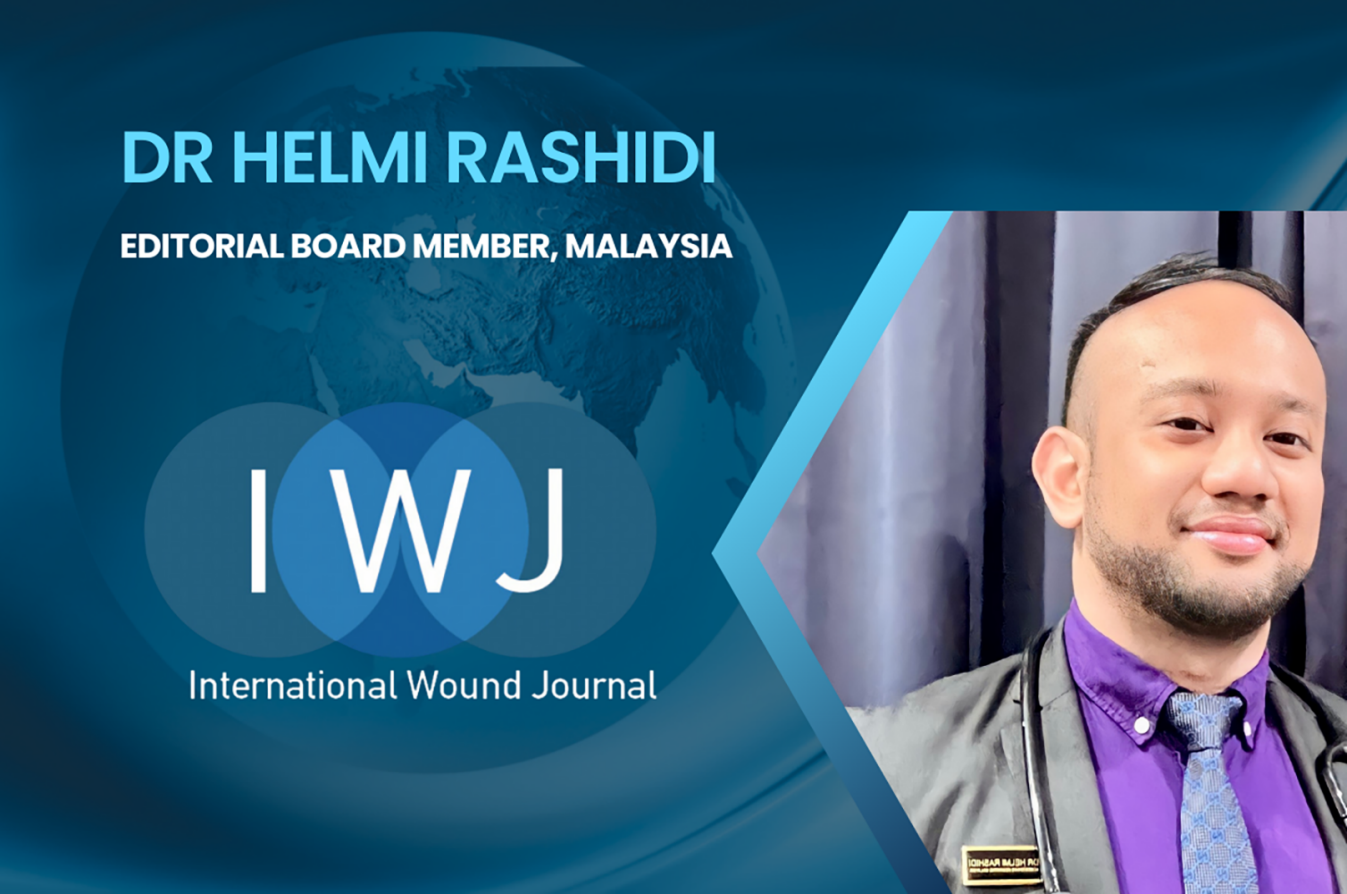
Dr. Helmi Rashidi, Malaysia

Dr. Mohamad Helmi Bin Mohd Rashidi is the Head of the Wound Care Unit at Hospital Ampang and an advanced wound care physician. He holds a Medical Degree from University Putra Malaysia, where he graduated in 2010. Furthering his expertise, he obtained a Certification of Clinical Wound Care and is now a Certified Wound Care Clinician. He also pursued a postgraduate diploma in wound management and is currently enrolling with the American Board of Wound Management to become a Certified Wound Specialist Physician.

Dr. Helmi specializes in chronic wound management and has made significant contributions to the field. He was nominated for an award in the Journal of Wound Care Awards 2024 and was elected as a clinical facilitator for the basic wound course program run by the Institute of Health Management, Austria, in Malaysia.

Additionally, he is a sought‐after speaker for both local and international wound care conferences.

Since 2017, Dr. Helmi has participated in numerous poster presentations on advanced wound care. His passion for wound care stems from a fascination with the healing process and the evolving field of wound care, an area still emerging in medical practice. His dedication and contributions are helping to raise awareness and advance the specialty of wound care.

**Figure:**
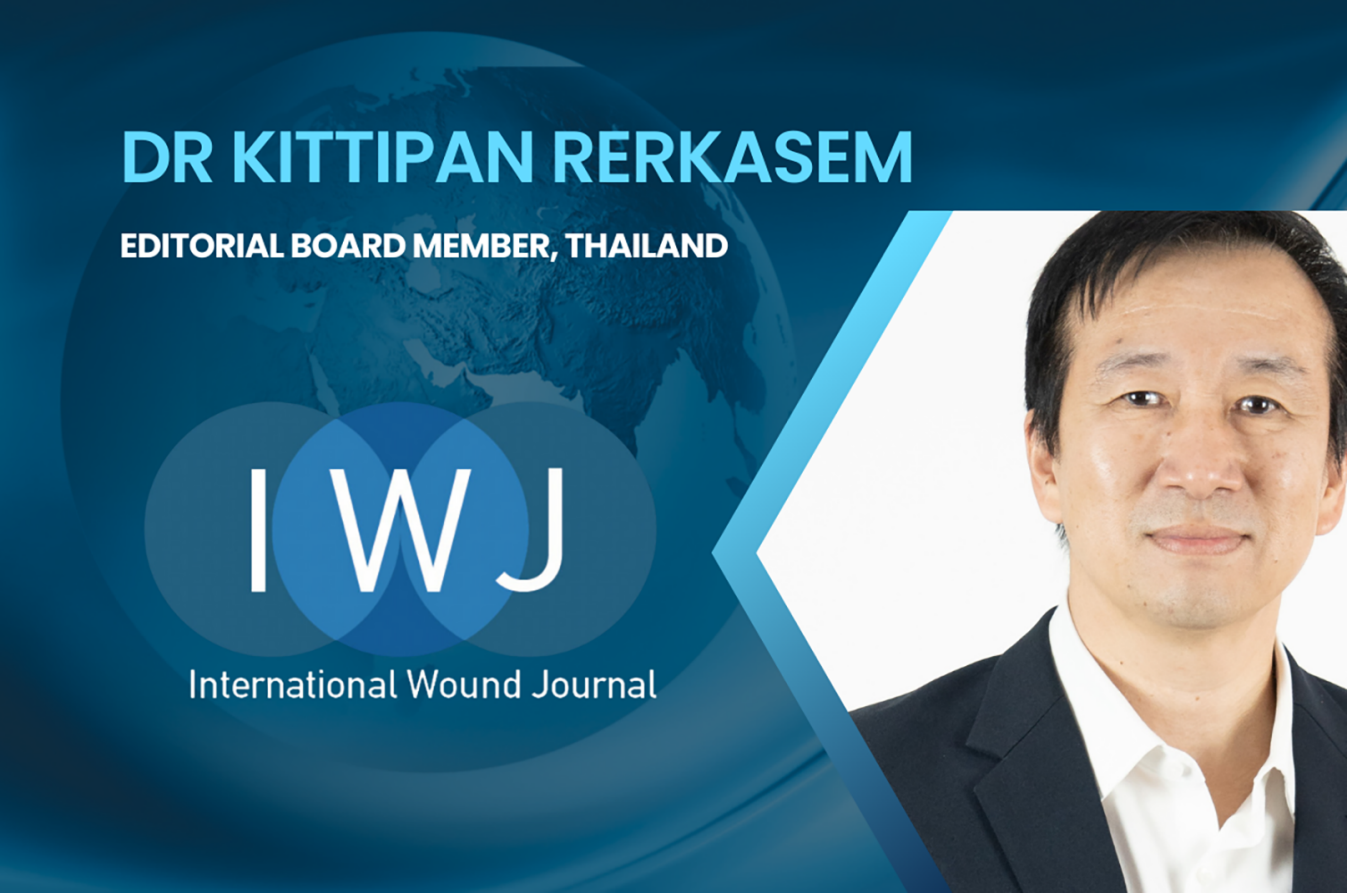
Dr. Kittipan Rerkasem, Thailand

Dr. Kittipan Rerkasem is a renowned vascular surgeon and a leading academic at Chiang Mai University, Thailand, where he holds the position of Deputy Director at the Research Institute for Health Sciences. With a medical degree from Chiang Mai University and further specialization in vascular disease from Southampton General Hospital and the University of Oxford, Dr. Rerkasem brings over two decades of experience in medical research and patient care.

His career is distinguished by his focus on non‐communicable diseases, particularly diabetes management and the prevention of major amputations and cardiovascular events. Dr. Rerkasem has been instrumental in developing innovative protocols for diabetic foot care, significantly reducing amputation rates at Chiang Mai University Hospital. His research extends into the study of cardiovascular risks in HIV patients and the epidemiology of vascular diseases within the Thai population.

Dr. Rerkasem has contributed to more than 100 peer‐reviewed publications and has been recognized with several national and international awards, including the Outstanding Researcher Award from the National Research Council of Thailand and inclusion in the World's Top 2% Scientists list by Stanford University.

An active member of various professional associations and currently an Associate Editor for the *International Journal of Lower Extremity Wounds*, Dr. Rerkasem continues to influence vascular surgery practices and research on a global scale.

**Figure:**
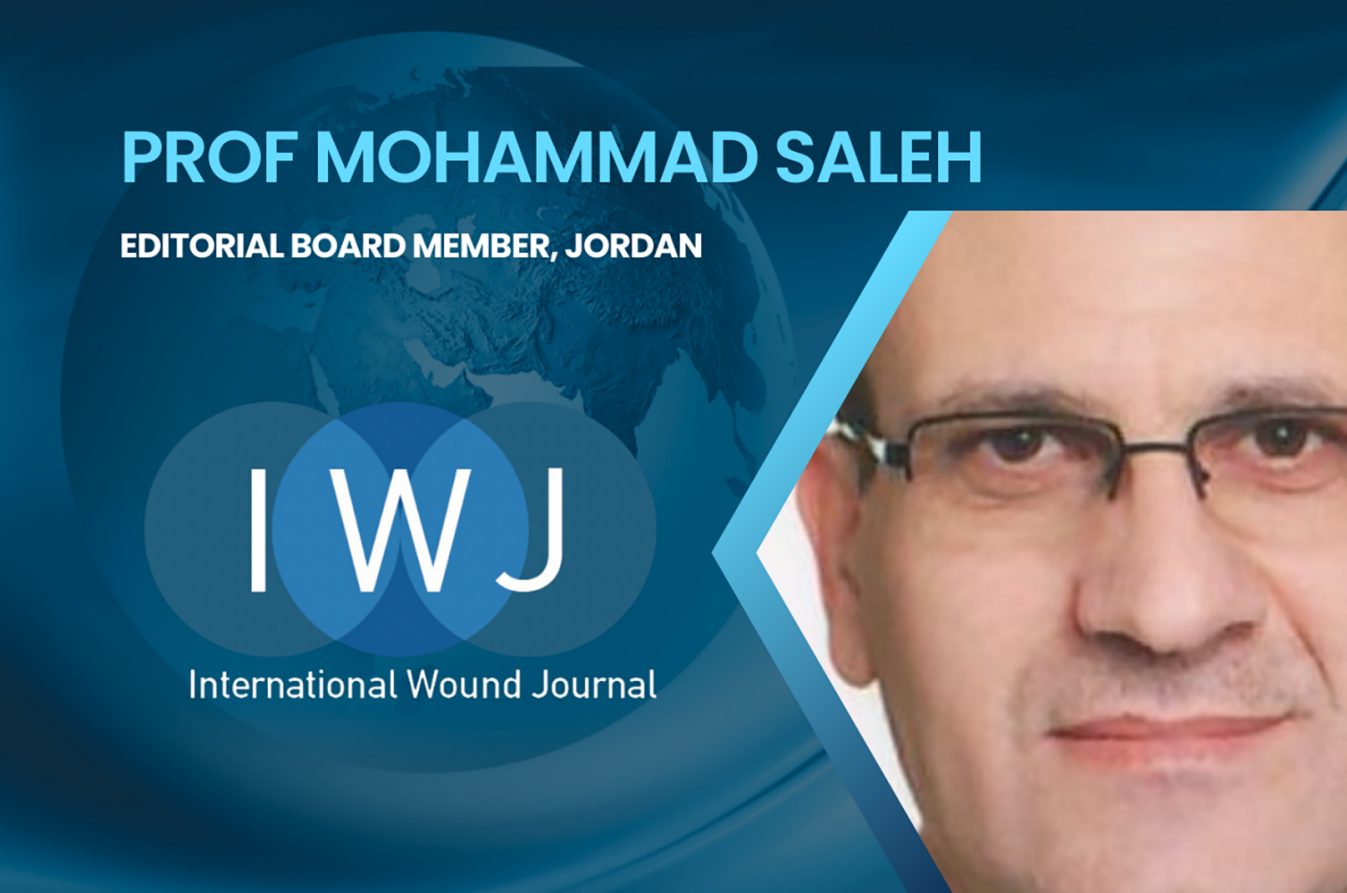
Professor Mohammad Saleh, Jordan

Mohammad is the Professor, School of Nursing, The University of Jordan and the Former Secretary General, JNC. He is also the former Vice Dean, School of Nursing, The University of Jordan and the coordinator of critical care master program, school of nursing, The University of Jordan.

Prof. Saleh would like to use his knowledge in health and life science profession to create new knowledge through research and close study of relevant population and disseminate this knowledge to the various communities he served utilizing critical thinking; therapeutic communication and appropriate teaching, management, consultative and advanced skills in the exercise of professional responsibilities. He would like to engage in the teaching process of several dimensions, such as critical care, public health, wound care, applied and epidemiologic research, community health, medical and surgical nursing, and in instructional development to contribute, not only to the general knowledge base in the field of nursing and health care but also to the ways in which knowledge is created and shared.

Prof. Saleh has authored and co‐authored several publications in international peer‐reviewed journals in the field of nursing and health care, including critical care, tissue viability and wound care management, education, management, school health, informatics, public and epidemiologic health research.

**Figure:**
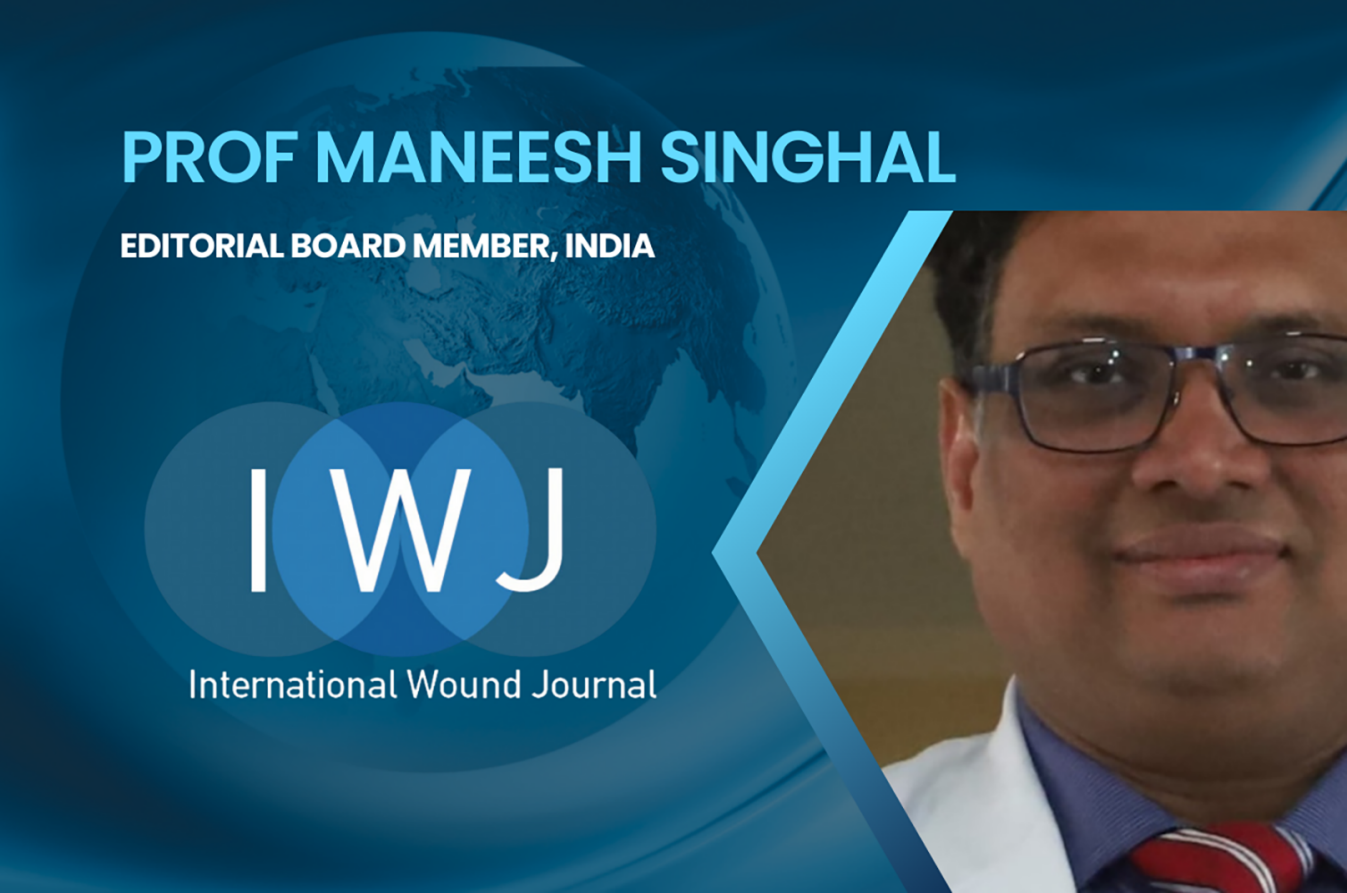
Professor Maneesh Singhal, India

Dr. Maneesh Singhal graduated from Maulana Azad Medical College; after that, he pursued his surgical training at Safdarjang Hospital. He finished his plastic surgery residency in 2005 following which he joined as a faculty member at AIIMS. He is presently an Associate Professor and Plastic and Maxillofacial Surgeon at Apex Trauma Center, AIIMS. His main interests are wound healing, trauma reconstructive surgery, maxillofacial injuries, complex wound management and negative pressure wound therapy. He has produced many national and international research publications, national and international paper presentations, chapters in books and research projects as investigator and coinvestigator funded by ICMR and AIIMS. He also has delivered many lectures in international and national meetings as guest faculty. He is one of the course directors of ATLS India program. He is also a recognized university undergraduate and postgraduate teacher and examiner.

**Figure:**
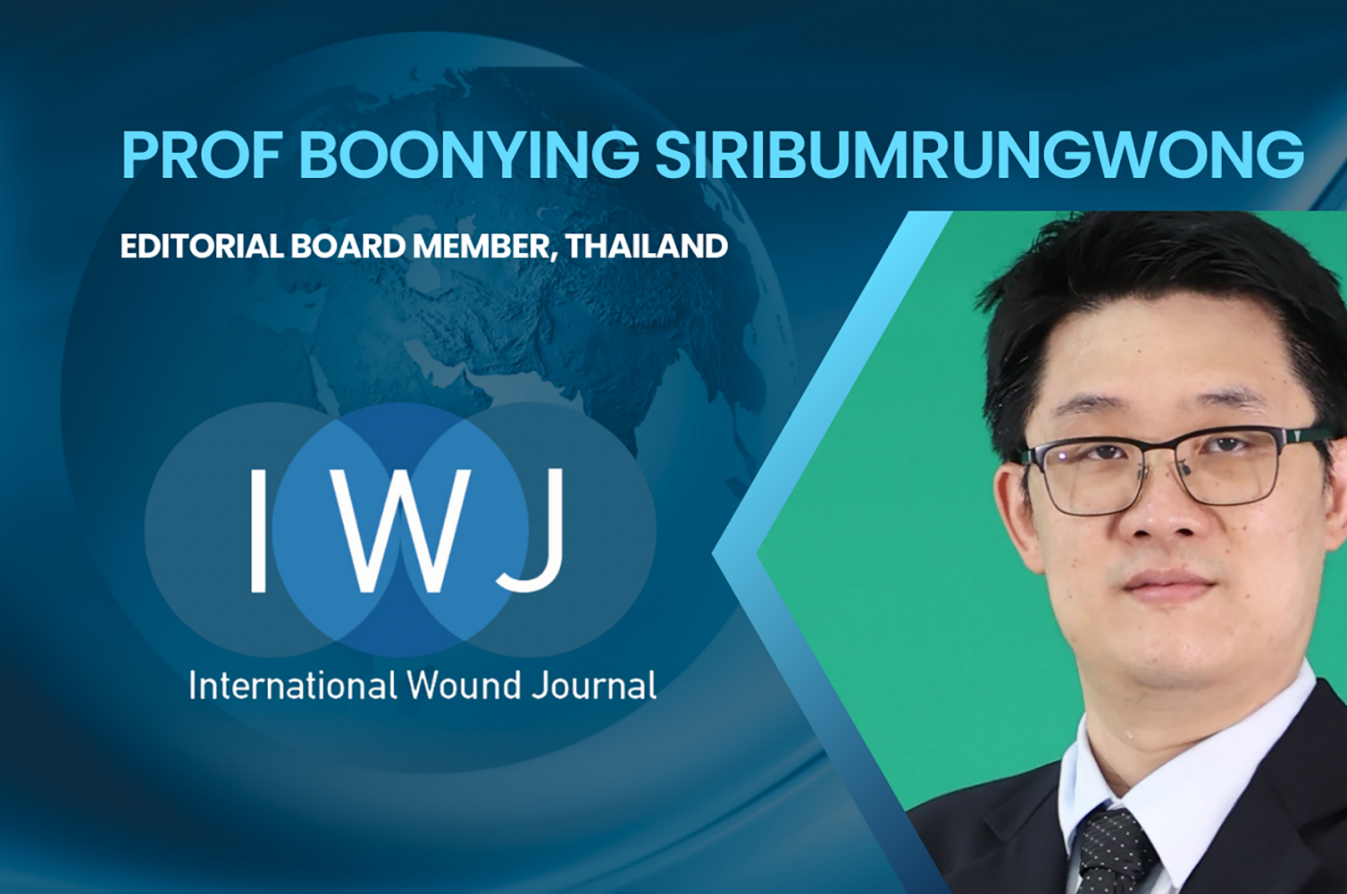
Professor Boonying Siriibumrungwong, Thailand

Dr. Boonying Siribumrungwong is an Associate Professor at Thammasat University in Thailand. He completed his Doctor of Medicine at Chulalongkorn University and Doctor of Philosophy at Mahidol University. He specialized in vascular surgery. He obtained his MD from Chulalongkorn University with first‐degree honours and then began his general surgical training at Chonburi Hospital. Upon completing the general surgical training, he worked as a lecturer and practiced clinically at Thammasat University Hospital. He has a strong interest in evidence‐based medicine and research, although his experience and knowledge in these areas were initially limited. Subsequently, he pursued further studies in clinical epidemiology and obtained a PhD in the field from Ramathibodi Hospital, Mahidol University. Following this, he received a certification in vascular surgery from the Medical Council of Thailand. Currently, his work primarily involves vascular surgery, with a focus on vascular access and peripheral artery disease (PAD) in diabetic patients. He now holds the position of Head of Division of Vascular Surgery in the Department of Surgery at Thammasat University Hospital.

**Figure:**
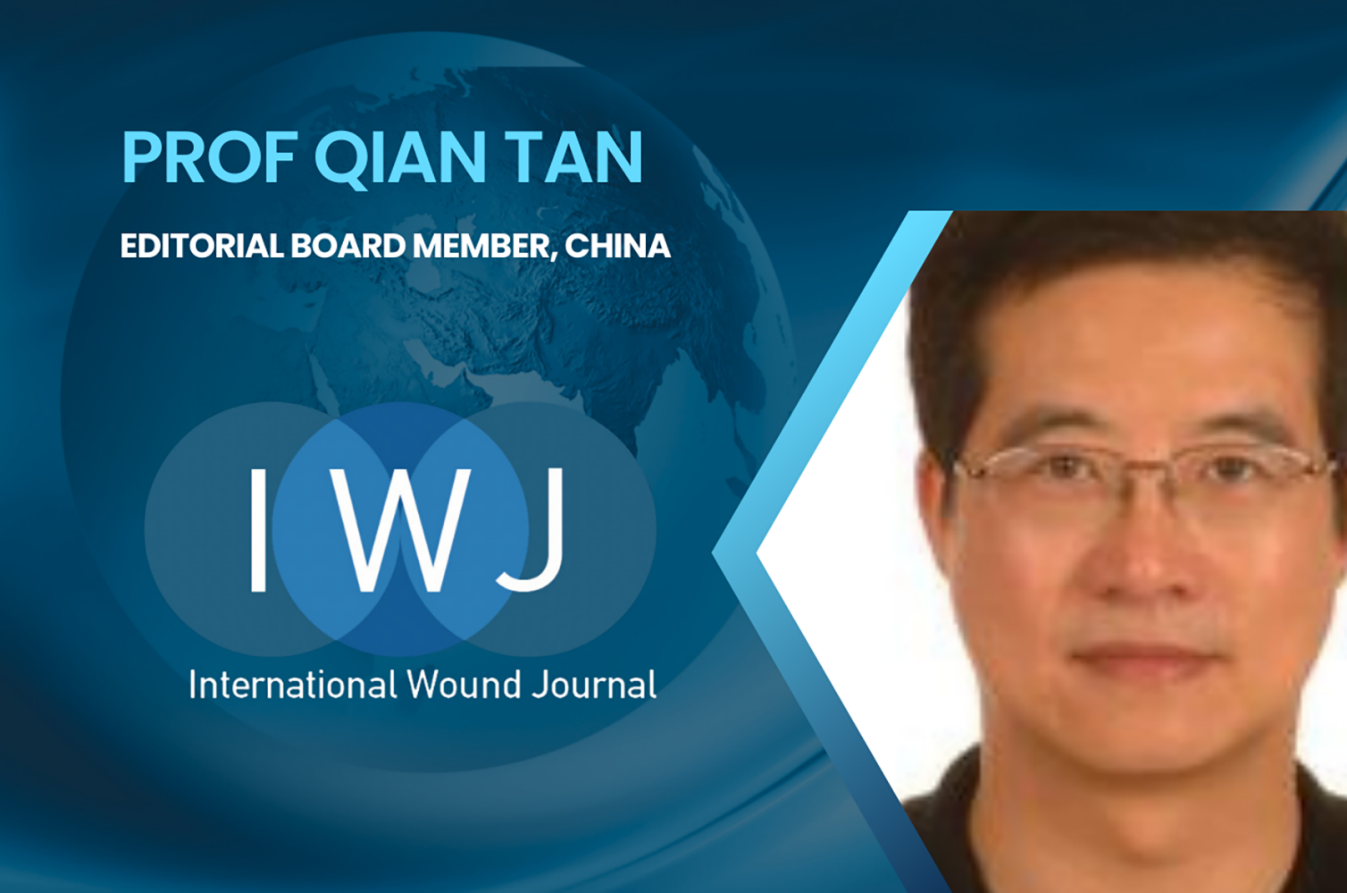
Professor Qian Tan, China

Professor Tan is the Chief Physician, Head of the Department, Plastic Surgery, Nanjing Drum Tower Hospital, The Affiliated Hospital of Nanjing University Medical School (January 2004 to present).

He specializes in plastic surgery, burn surgery, cosmetic surgery and chronic wound treatment. His specific expertise includes repair and reconstruction; wound treatment and eye, nose, breast and perineum cosmetic plastic surgeries. As a senior volunteer of ‘Operation Smile’, Prof. Tan has treated more than 2000 children with cleft lip and cleft palate for free.

Professor Tan is a Member of Plastic Surgery Branch of Chinese Medical Association; Member of Chinese Medical Doctor Association; Member of Chinese Association of Plastics and Aesthetics; Member of WSRM and a Member of ISBI.

**Figure:**
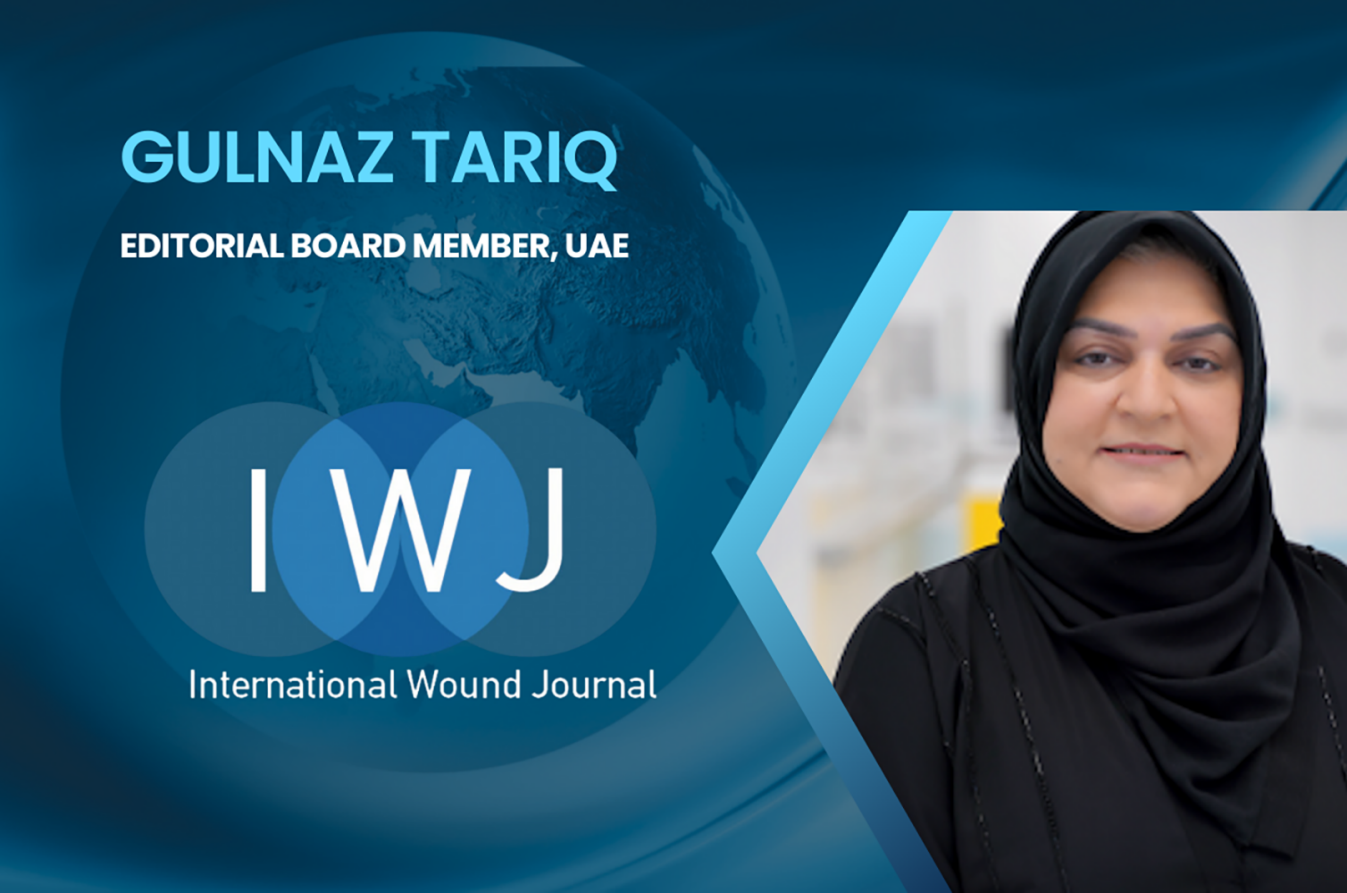
Gulnaz Tariq, UAE

Gulnaz Tariq is the Unit Manager for Wound Care in Sheikh Khalifa Medical City, Abu Dhabi. She trained as a nurse in the armed forces and graduated with First Position in Province, Post‐graduate with honours. She has pursued further international wound care training by joining the International Inter‐professional Wound Care Course (IIWCC) from the University of Toronto in 2007. She completed her MSc in Skin Integrity from the University of Hertfordshire (UK) in 2014. She is a local, regional and international wound care key opinion leader and speaker and is the founder (2015) and course director of the Ostomy Care and Management (OCM). She introduced a diabetic foot prevention program and pressure ulcer prevention program in SKMC and has organized the Abu Dhabi Wound Care Conference for the past 9 years. Gulnaz is the Founder of the International Inter‐professional Wound Care Group and was elected to be President. She has won the Bid for Abu Dhabi for 2020 to bring World Union Wound Healing Society Congress to the region and was elected as President Elect of WUWHS.

**Figure:**
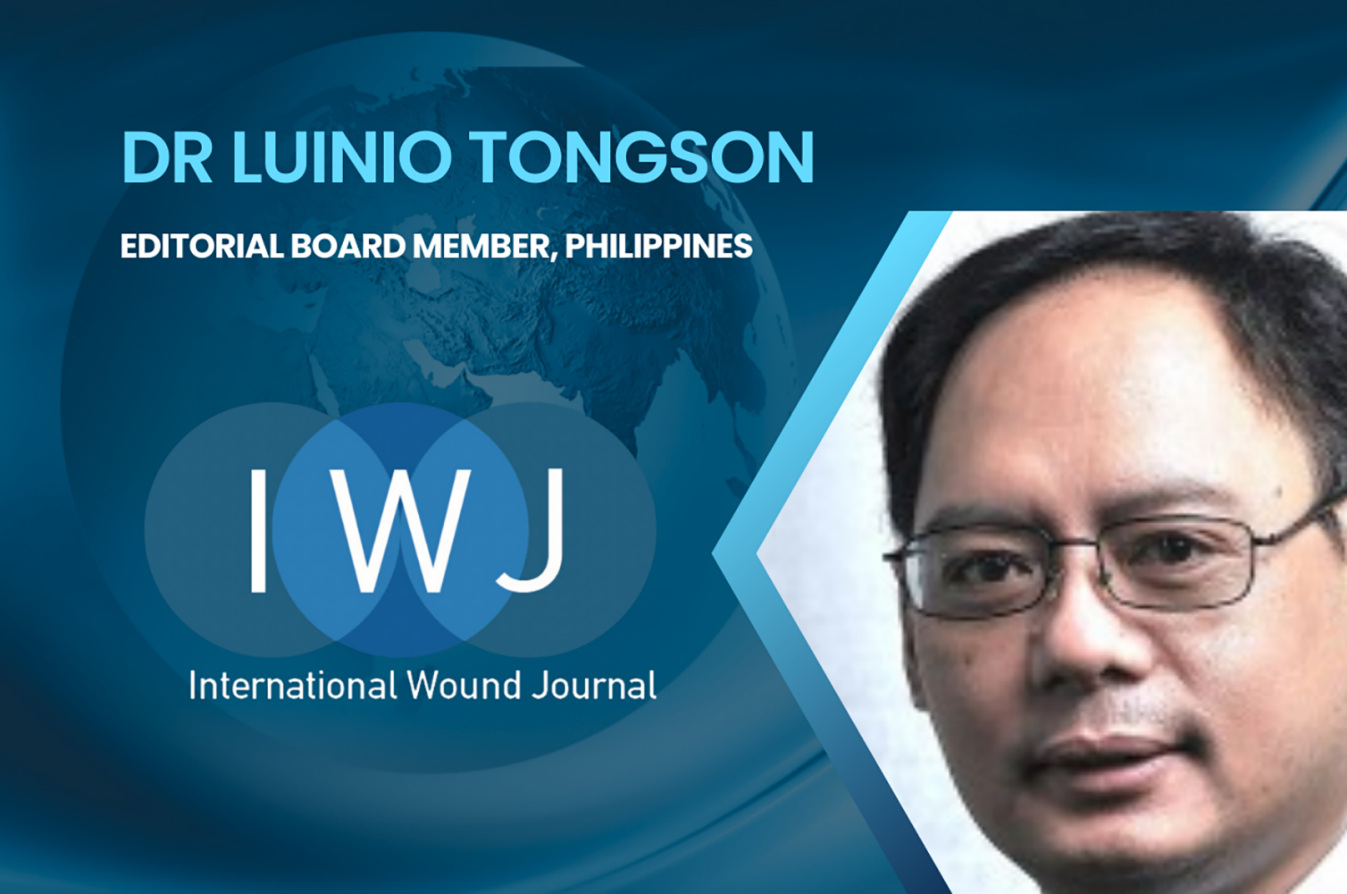
Dr. Luinio Tongson, Philippines

Dr. Tongson is the Past President, Philippine Wound Care Society; an Executive Member, World Union Wound Healing Society; and the Philippine Representative, Diabetic Foot International.

He serves as the Head, Dr. James G. Dy Wound Healing and Diabetic Foot Center, Chinese General Hospital and Medical Center, and the Head, Diabetic Foot and Wound Care Center and Capitol Medical Center. He is an active consultant in General Surgery at the Chinese General Hospital and Medical Center, the Capitol Medical Center and the St. Luke's Medical Center.

**Figure:**
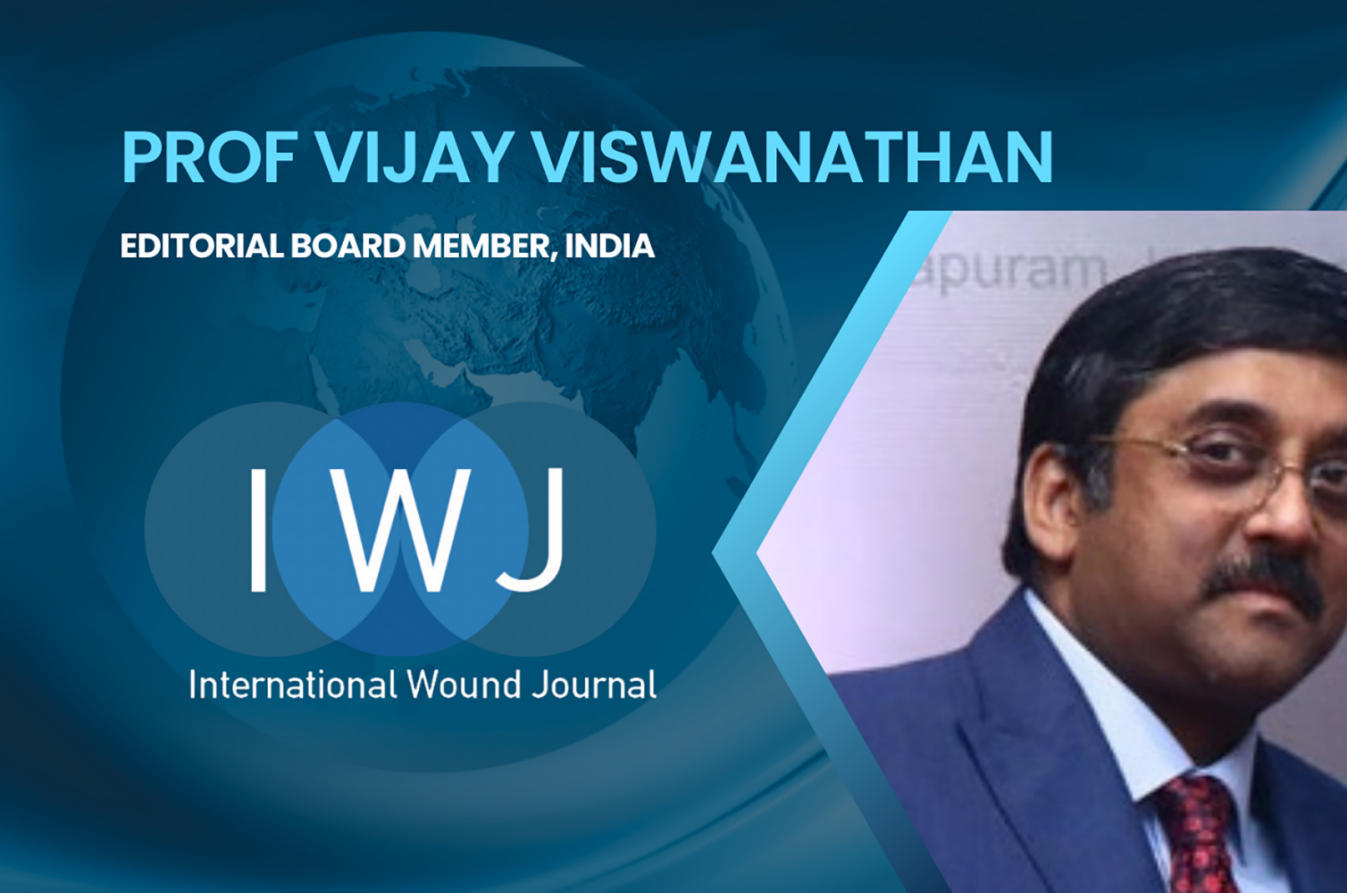
Professor Vijay Viswanathan, India

Vijay is the Head and Chief Diabetologist of M.V. Hospital for Diabetes and President of Prof. M. Viswanathan Diabetes Research Centre, Royapuram, Chennai, a WHO Collaborating Centre for Research, Education and Training in Diabetes.

Professor Viswanathan obtained a PhD in Diabetic Nephropathy from Tamil Nadu Dr. M.G.R. Medical University after an MD in Internal Medicine. He received the first Vivian Fonseca Award from the American Diabetes Association for his work in DM and TB in 2013. He has trained over 2000 physicians in prevention of amputations.

He was appointed Adjunct Professor by Tamil Nadu Dr. M.G.R. Medical University. He was also awarded Fellowship of the Royal College of Physicians London for Research Contribution in Diabetes. He is the editor of *Journal of Association of Physicians of India*, Tamil Nadu Chapter (TAPIJ) from 2009. He is the Chairman of API, Tamil Nadu Chapter, for the period 2010–2011. He was elected as the National Governing Body Member of API for the second time in succession. He was the Secretary of RSSDI Tamil Nadu Chapter from 2013 to 2017 (August). He is also a National Executive Committee Member, RSSDI, from 2016.

Professor Viswanathan is the immediate past‐President of D‐Foot International.

He is credited with around 270 research publications on various aspects of diabetes and its complications in peer‐reviewed national and international scientific journals and has also contributed about 25 book chapters.

**Figure:**
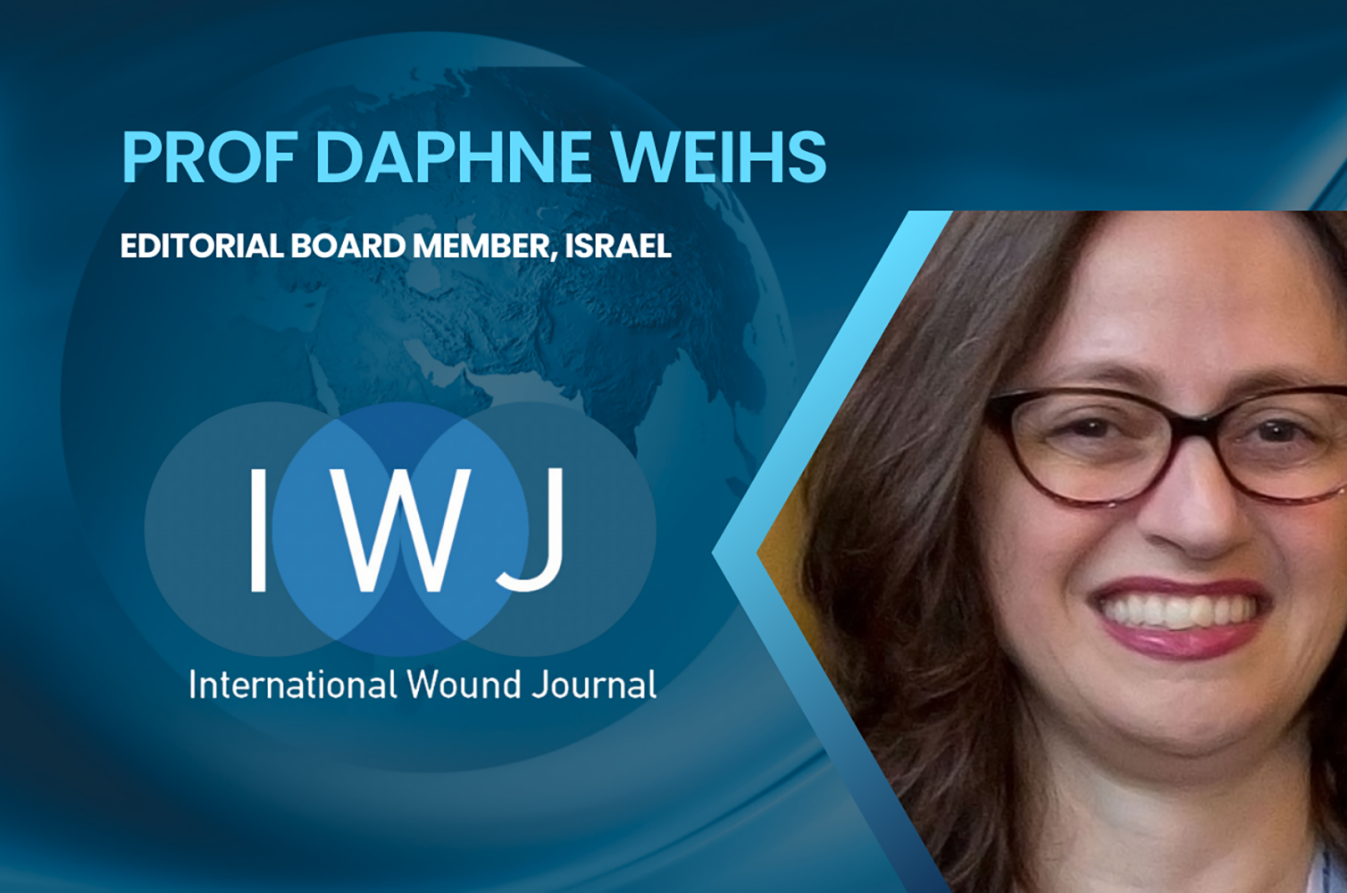
Professor Daphne Weihs, Israel

Prof. Weihs is an Associate Professor at the Faculty of Biomedical Engineering, Technion‐Israel Institute of Technology, Haifa Israel. She received her BSc (Cum Laude), MSc (Cum Laude) and PhD (2004) in Chemical Engineering from the Technion‐IIT. She then continued as a postdoctoral researcher at the Department of Pathology and Lab Medicine, School of Medicine at the University of California at Los Angeles (UCLA).

Prof. Weihs' research interests are in mechanobiology of cancer and wounds, focusing on approaches of cell mechanobiology and tissue mechanics to predict cancer metastasis and to prevent wounds and accelerate healing. She has published over 55 peer‐reviewed articles in the top biomedical engineering and biophysical journals and has presented over 175 seminars and conference talks (including invited and keynote talks). Prof. Weihs is the Past President of the Israel Society of Medical and Biological Engineering and serves on the editorial board of the journal *PLOS One* (PLOS publishing) and *Medical Engineering and Physics* (Elsevier publishing). Her research is funded by highly competitive governmental scientific funding agencies such as the Israel Ministry of Science and Technology (MOST), the Israel Ministry of Health (MOH) and the Israel Ministry of Economics. Among her honours and awards are the Henri Gutwirth Research Grant Award and listing as one of the 50 Most Influential Women in Israel, Globes (2015).

**Figure:**
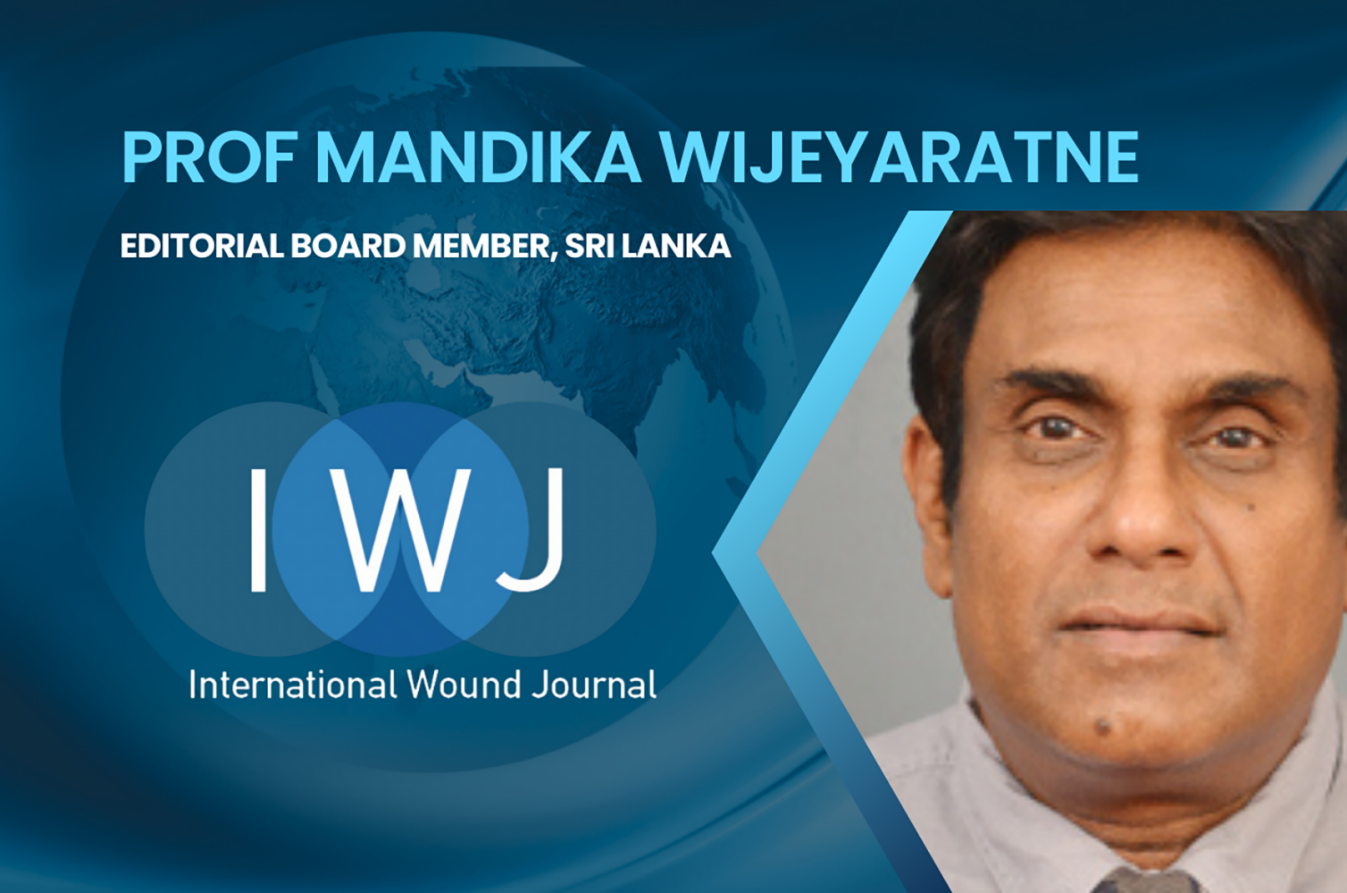
Professor Mandika Wijeyaratne, Sri Lanka

Mandika Wijeyaratne, MS, MD, FRCS, is the Chair Professor of Surgery, at The Faculty of Medicine, University of Colombo, and chief of the University Vascular and Organ Transplant program. He received his medical education at the University of Colombo and then went on to specialize in surgery and was board certified by the PGIM in Colombo in 1990. He then joined the Department of Surgery academic staff and then in 1999 proceeded to research carotid bifurcation plaques and obtain MD by thesis at the University of Leeds, England. Thereafter, he introduced carotid endarterectomy surgery for stroke prevention in Sri Lanka. Other key contributions to vascular surgery were the introduction of colour duplex vascular imaging and extreme distal bypass surgery for amputation prevention, particularly in the aging diabetic population. All these procedures are in mainstream practice today. He also contributes exhaustively to wound care education and the development of guidelines for the management of the diabetic foot locally and internationally.

In parallel with his interests in vascular disease, he is also interested in expanding the horizons of organ transplantation surgery in Sri Lanka. This resulted in visits to centres of excellence in liver transplantation at the Starzl Institute in Pittsburgh, Leeds liver unit and Medanta in New Delhi. This experience led to the first successful liver transplant operation in Sri Lanka in 2010. He has also extended the kidney transplant program to the Army Hospital and the Lady Ridgeway Hospital for children. To date, he has performed over 1200 kidney transplants in Sri Lanka.

He has lectured extensively throughout the country and in Asia on outcomes of distal bypass surgery and wound care. He was the principal investigator in a first‐in‐man study of a polyurethane vascular graft for early puncture. His other areas of research include attenuation of ischaemia reperfusion injury and development of novel wound dressings.

He has first‐author publications in the BJS, JVs and EJVES. He has also authored book chapters on vascular injuries in Sri Lanka, the diabetic foot and the diagnosis of abdominal aortic aneurysms and won many awards including several orations that include the SLMA oration.

**Figure:**
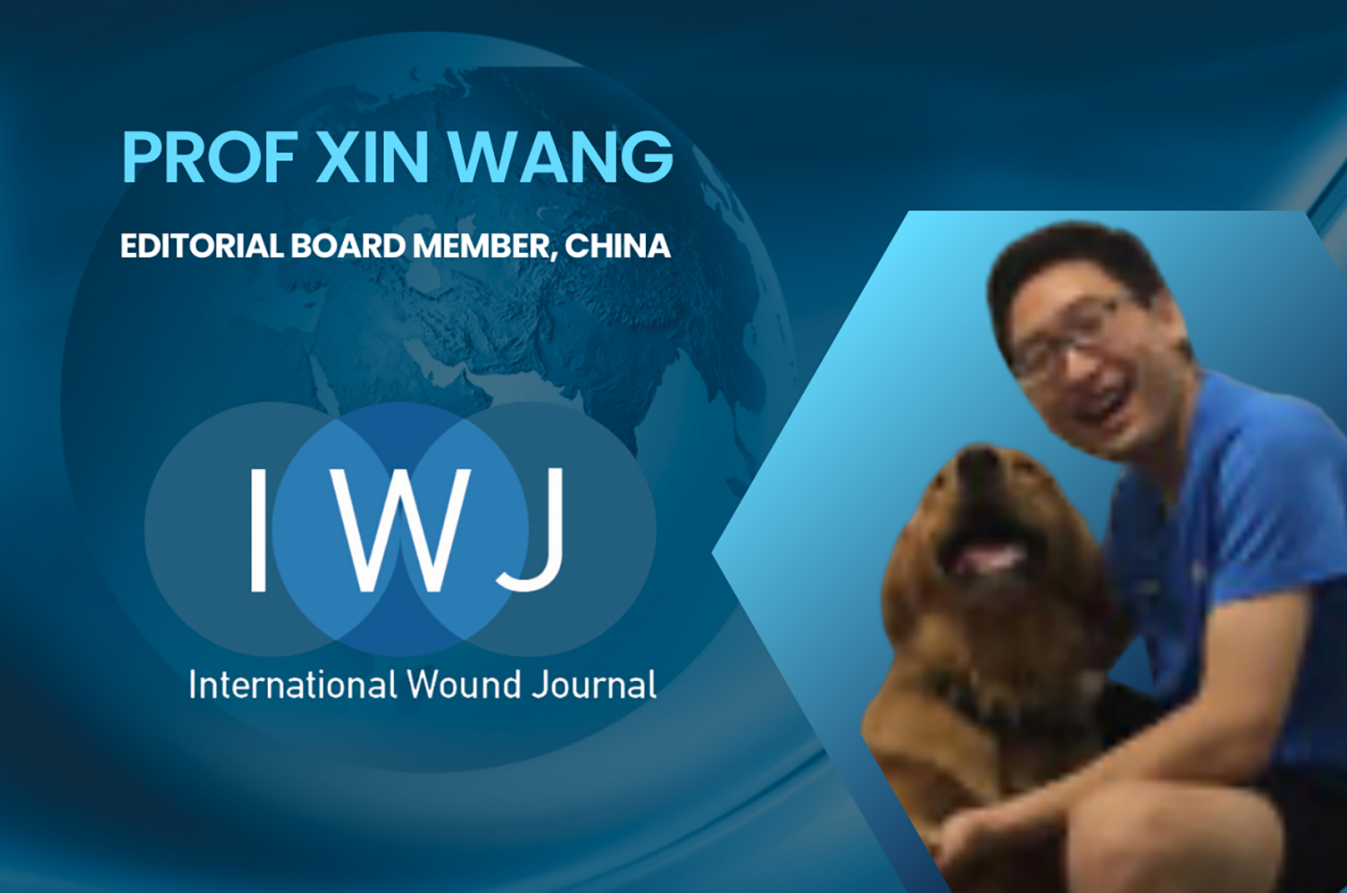
Professor Xin Wang

Dr. Xin Wang is a plastic and hand surgeon, specializing in plastic and reconstructive surgery and hand surgery. He is a Professor of Surgery at the University of NingBo China and the Director of the Department of Plastic and Reconstructive Surgery and Hand Surgery at NingBo Sixth Hospital. His special interests include perforator flaps, thumb and finger reconstruction, congenital hand, bone and joint reconstruction of the wrist and elbow, cosmetic surgery, expertise in reconstruction of post‐burn sequel and scar treatments of upper and lower extremities. He is committed to providing excellent treatment and care to his patients, both in plastic and hand surgery and reconstructive microsurgery.

**Figure:**
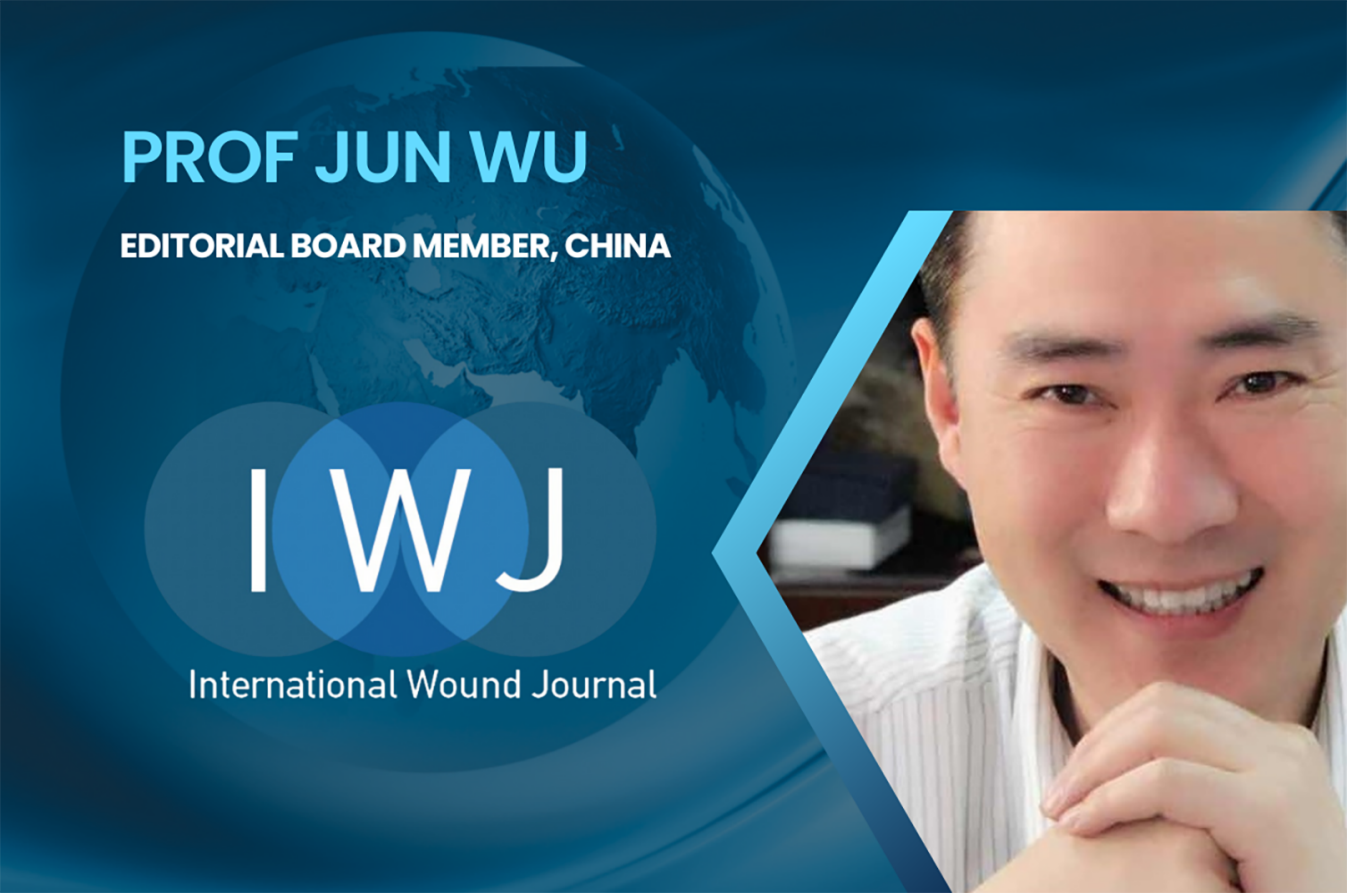
Professor Jun Wu, China

Professor Wu is the Director, Professor, Department of Burn and Plastic Surgery, the First Affiliated Hospital, Shenzhen University, China. He is the past president of the Chinese Burn Association, a past president of the Chinese Burn Rehabilitation Association, the president of Chinese Society for Wound Healing and the Editor‐in‐Chief of Burns and Trauma.

He has received numerous awards in his career: The 24th China Patent Excellence Award (2023); Lok Nayak Hospital Visiting Professorship, issued by the National Academy of Burns India (2022); First‐class Science & Technology Award for Research and application of new technology for wound diagnosis, issued by the Chinese Rehabilitation Association (2021); First‐class Science & Technology Award for New Mechanism of Skin Wound Healing and Translational Medicine Research, issued by the Chinese Medicine Association (2021); Second‐class Science & Technology Award of National Science and Technology Advancement for the novel management of severe burn injury, issued by China Central Government (2006); and the Williams Prize for the best research prolongation of the survival of alloskin grafts without any immune suppression, issued by the Israel Burn Association (1992).

Professor Wu has received over 147.6 million CYN in research grants and contracts. His research covers the area of burn management and rehabilitation, regenerative medicine, biomaterials for wound dressing, allo/xeno‐skin transplantation immunology and multi‐spectral imaging devices for burn wound precision evaluation. He has over 101 publications.

**Figure:**
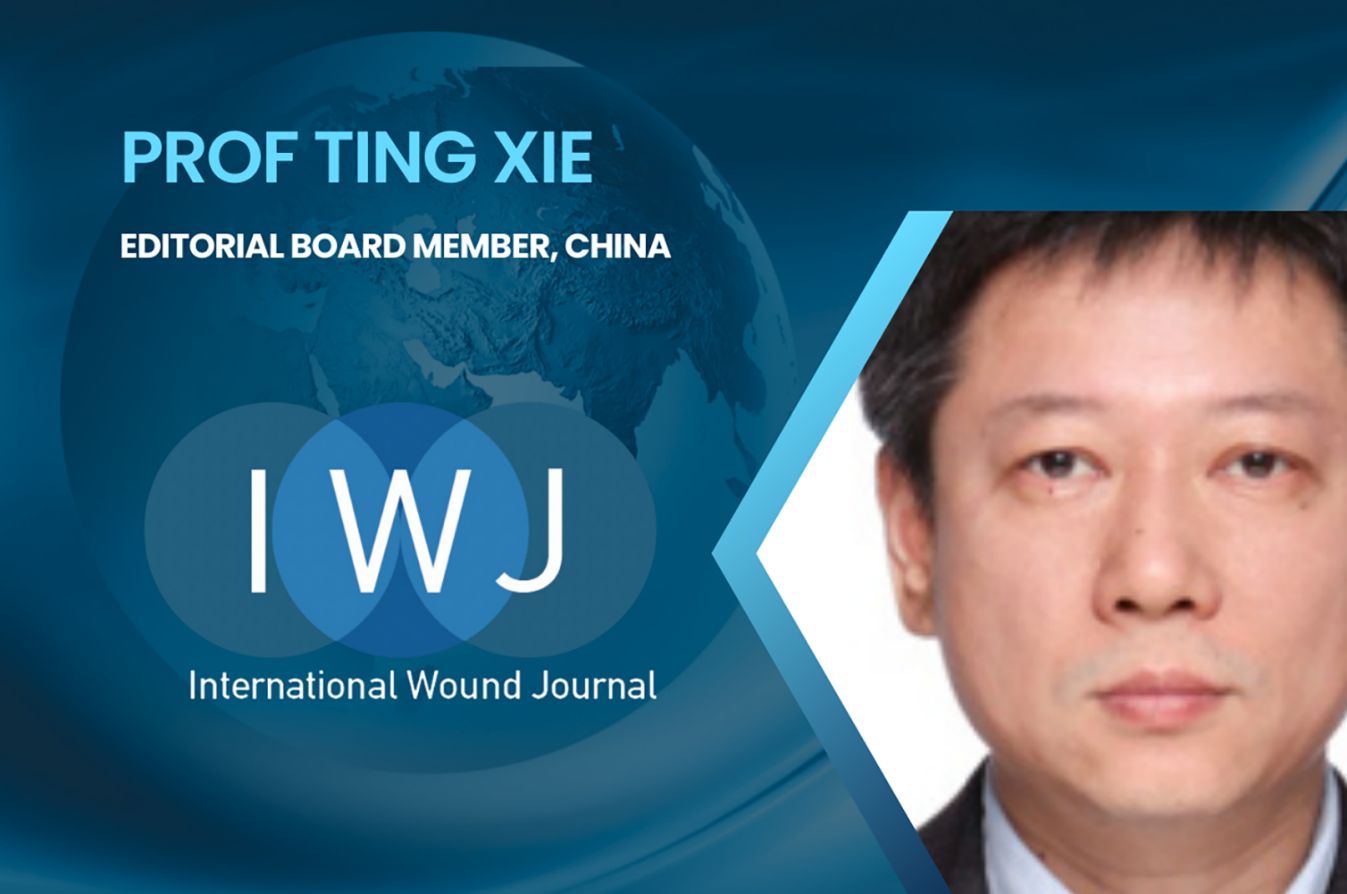
Professor Ting Xie

Wound healing has been his interest for more than 30 years. For research on basic science, the main work involved tissue repair and regeneration with chronic wounds. He led and joined a series of national and international research programs. As the pioneer, he initiated the first wound healing department in China and established the critical principle of diagnosis and treatment of chronic wound diseases, which has been gaining nationwide recognition. As the vice president of the Chinese Tissue Repair Society and secretary of the Asian Wound Care Association, he is well known as a clinical expert of a national and international reputation.

**Figure:**
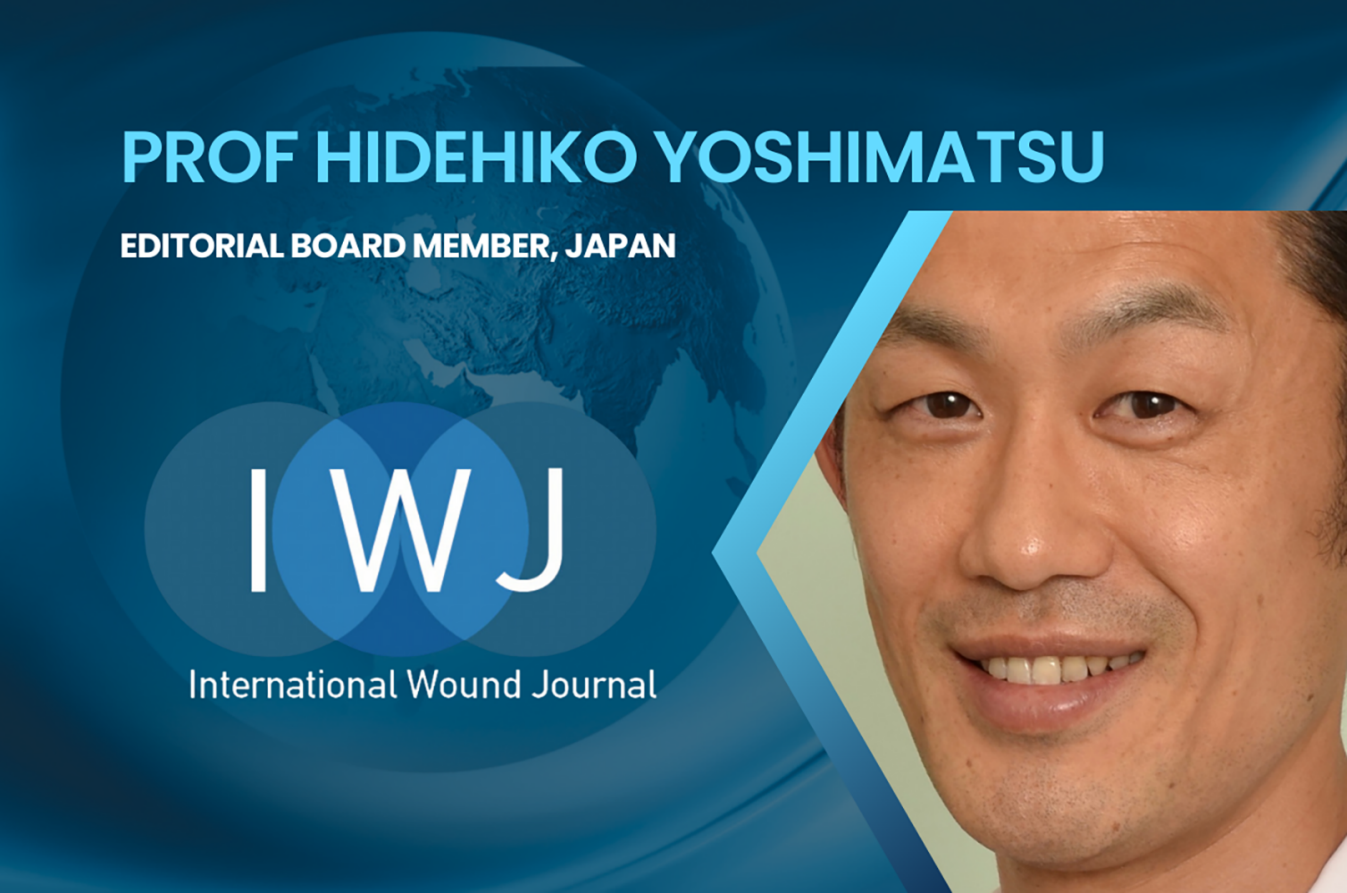
Professor Hidehiko Yoshimatsu, Japan

Professor Yoshimatsu is a plastic surgeon with expertise in microsurgery and super microsurgery of lymphatics and other subspecialties of reconstructive surgery. After graduating from the University of Tokyo Faculty of Medicine, he completed his plastic surgery training at the University of Tokyo Hospital. Prof. Yoshimatsu's work extends across several specialties, including microsurgery of the breast, the lymphatic system and the extremities.

**Figure:**
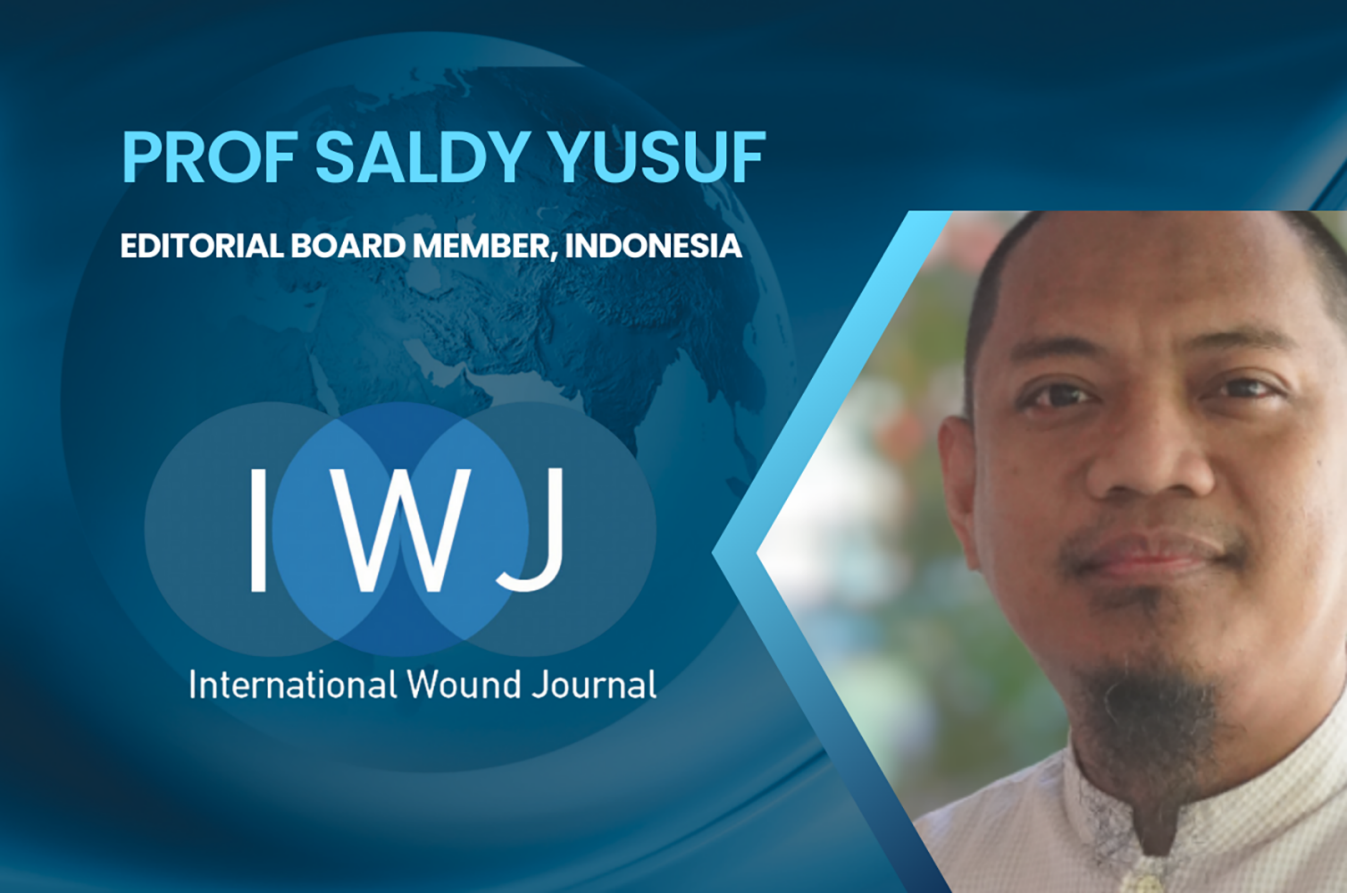
Professor Saldy Yusuf, Indonesia

Saldy Yusuf is an accomplished individual with a diverse academic background in Nursing Science. He holds a Bachelor's degree from the Faculty of Medicine, Hasanuddin University, which he completed in 2007. He furthered his education by pursuing a Master's and Doctorate in Chronic Wound Management from Kanazawa University, Japan, graduating in 2012 and 2016, respectively. As a dedicated researcher, Saldy Yusuf has made significant contributions to the field of nursing, particularly in the management of chronic wounds and diabetes‐related foot ulcers. His scholarly work has been recognized globally, with publications in esteemed journals and databases such as Scopus, PubMed, Web of Science, Sinta and Google Scholar.

Beyond academia, Saldy Yusuf is an active member of several prestigious organizations. He serves as an International Delegate for the World Council of Enterostomal Therapy Nurse (WCET) and holds a position in the Education Committee of WCET. Additionally, he is the Chairperson of the Research Division at DPP In WOCNA. These roles demonstrate his commitment to advancing nursing practices and knowledge dissemination on an international level.

As an esteemed researcher and academic, Saldy Yusuf has been invited to review articles for numerous reputable journals. His expertise as a reviewer spans a wide range of topics, including wound care, diabetes management, nursing and healthcare organization. Saldy Yusuf's scholarly endeavours and dedication to advancing healthcare practices have had a significant impact on the nursing community and continue to contribute to the improvement of patient care and management worldwide.

**Figure:**
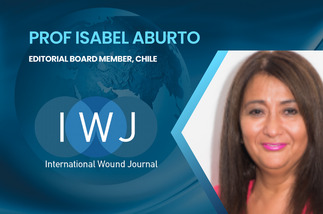
Professor Isabel Aburto, Chile

Isabel Aburto Torres is a Nursing graduate from the University of Concepción. She has served as the creator and director of the National Institute of Wounds Foundation since 2005. This institution is dedicated to training, research, publishing magazines and clinical guides and providing patient care for those with complex wounds and ulcers.

Ms. Aburto Torres holds multiple diplomas in management and is a Wound Management Specialist. Since 1998, she has been an advisor to the Ministry of Health of Chile, contributing to the development of the GES Diabetic Foot Baskets and Valued Benefits for Venous Ulcers. She also evaluates FONDEF research work and has authored 15 clinical guides and 14 journals on wound and ulcer management.

In her academic role, Ms. Aburto Torres is a professor of various postgraduate courses in wound management and has directed 11 national and international congresses on this subject. In 2023, she signed an international agreement with Spain to enhance training programs.

As a researcher, Ms. Aburto Torres has participated as an author in over 18 research projects on the management of wounds and ulcers. She has initiated several social campaigns, including National Wounds Day, which has been held for 13 years in Chile to educate the public on wound prevention. In 2010, she started the Wound Solidarity Route through Chile, aimed at training healthcare professionals from various regions through patient interaction. This initiative expanded to Haiti in 2019, incorporating international volunteering.

Ms. Aburto Torres established National Ostomy Day in Chile in 2018. In 2017, she founded the Society of Latin American Wound Nurses (SELH) with support from the Asian Wound Society. In 2023, she oversaw the creation of the Specialty in Wound and Ostomy Management.

**Figure:**
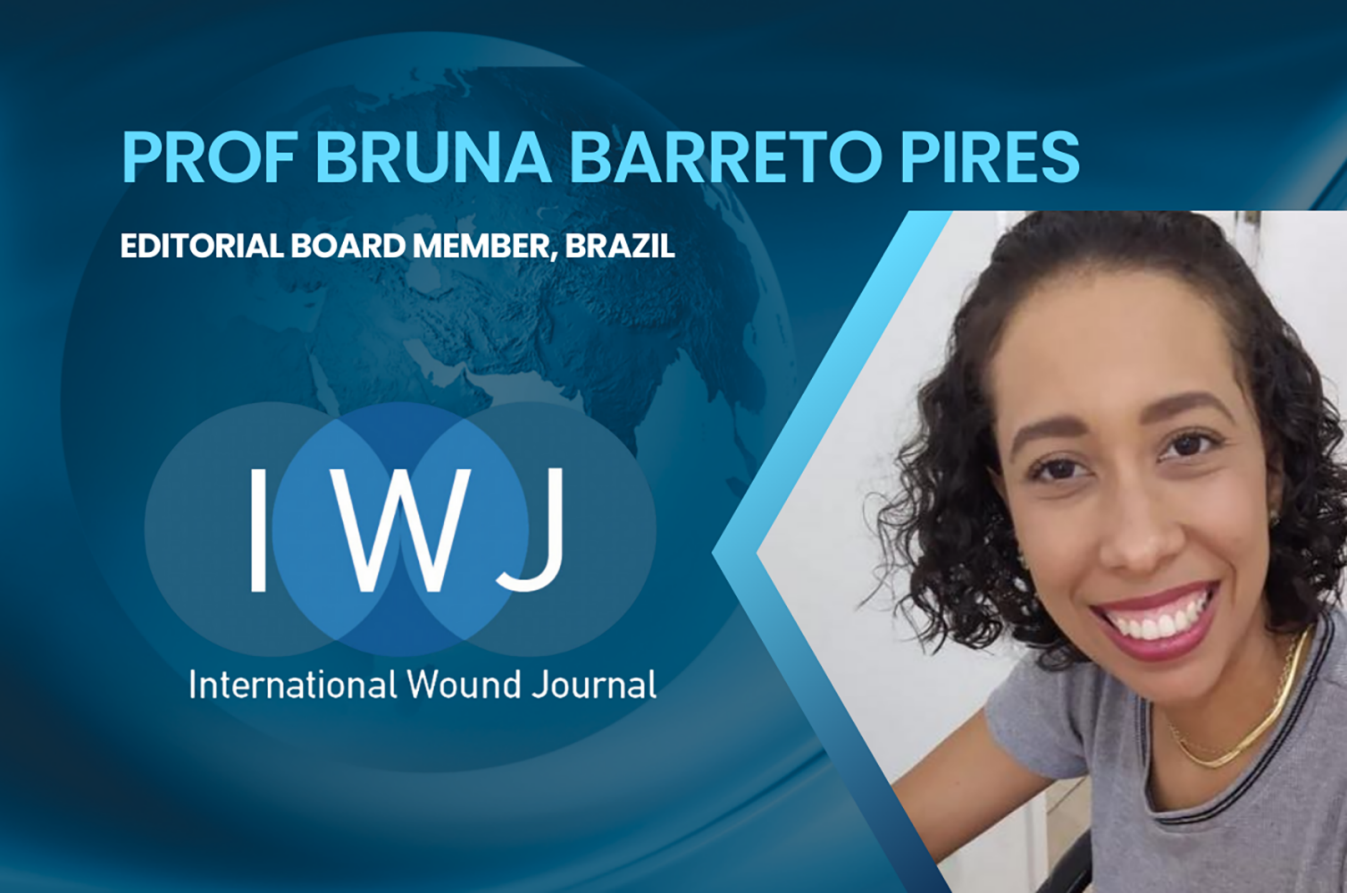
Professor Bruna Barreto Pires, Brazil

She received his Bachelor's and Licentiate's Degree in Nursing from the Fluminense Federal University (2013). She is a specialist in infection control in healthcare—UFF (2015) and in intensive care (2016)—UVA. She has a PhD and Master's in Health Care Sciences with a focus on clinical research, wounds and microbiology from UFF. In her master's and doctorate degrees, she developed research in the area of molecular and clinical diagnosis, colonization/infection in wounds by MRSA and *Pseudomonas aeruginosa*, as well as microbial profile and microbial clonal diversity. She was an adjunct professor at the Fluminense Federal University in the Department of Nursing and Administration Fundamentals and is currently vice‐coordinator of the undergraduate nursing course at UFF (2021–2025). She was a professor at the Rio de Janeiro State University (UERJ) in the Department of Medical‐Surgical Nursing in the area of clinical nursing from 2019 to 2020. She is a reviewer for the journals *The Nurse Practioner*, *Revista de Medicina de Ribeirão Preto*, *Journal of Pure and Applied Microbiology* (JPAM), *Advances in Skin and Wound Care*, *Revista de Enfermagem Anna Nery*, *ABCS Health Sciences*, *Revista Norte Mineira de Enfermagem* and *REBEN*. She is a member of the editorial board of Advances in Skin and Wound Care (Peer Review Panel) and Frontiers in Clinical Diabetes and Healthcare. She is a vice‐leader of the CNPq research group titled Clinical Research for Systematization and Innovation in Health and Nursing Care. She is a researcher in the research group CICATRIZAR—Clinical Research, Wounds and Biomaterials.

**Figure:**
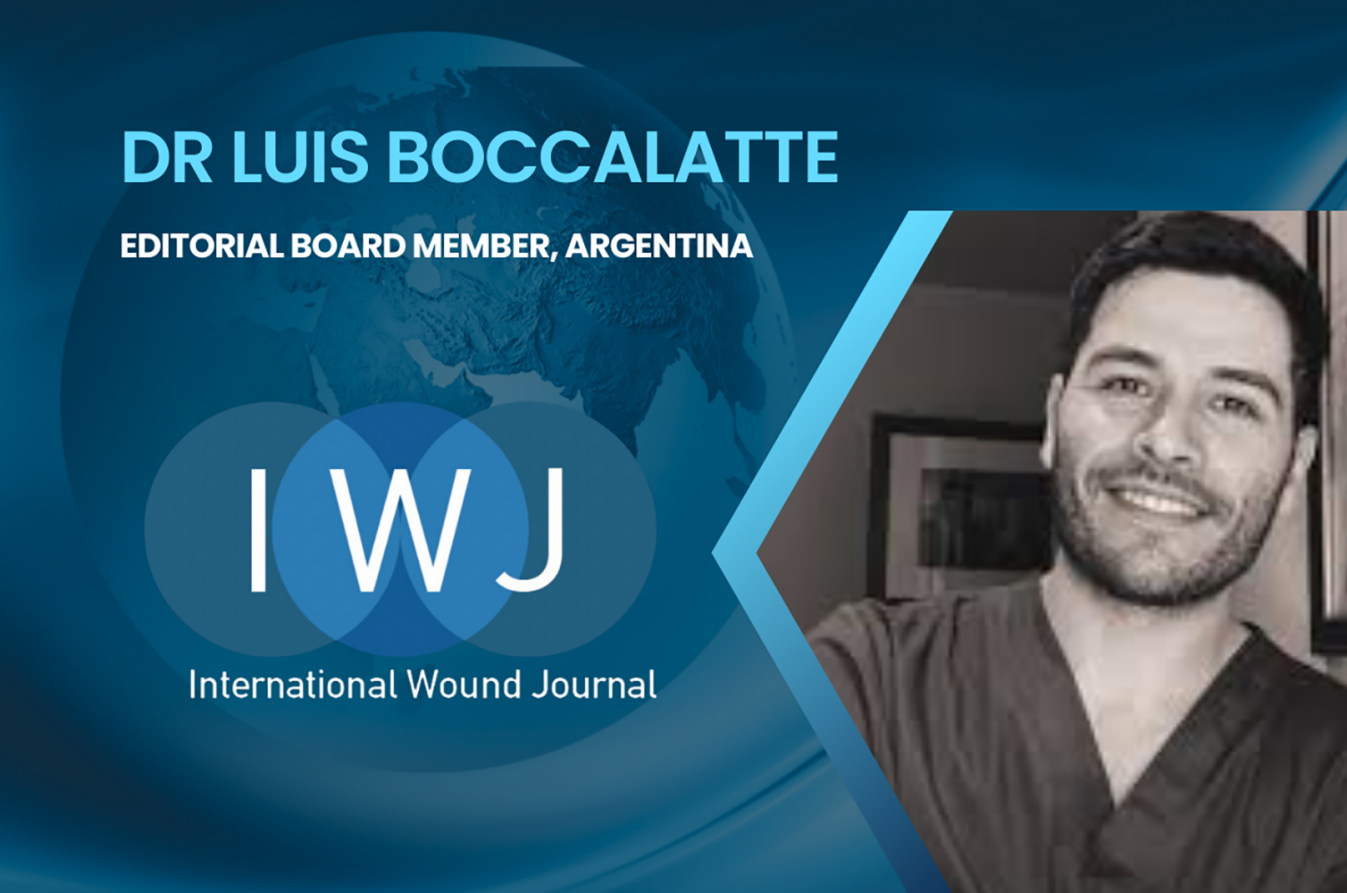
Dr. Luis Boccalatte, Argentina

Dr. Luis Boccalatte received his medical degree with honours from the University of Buenos Aires in Argentina. He then completed his residency in general surgery at the Hospital Italiano de Buenos Aires. Following his surgical training, Dr. Boccalatte completed a fellowship in head and neck, maxillofacial and facial reconstruction at the same institution. He currently serves as an Associate Surgeon of Surgery at Hospital Universitari Sagrat with an active clinical and research interest in HN.

**Figure:**
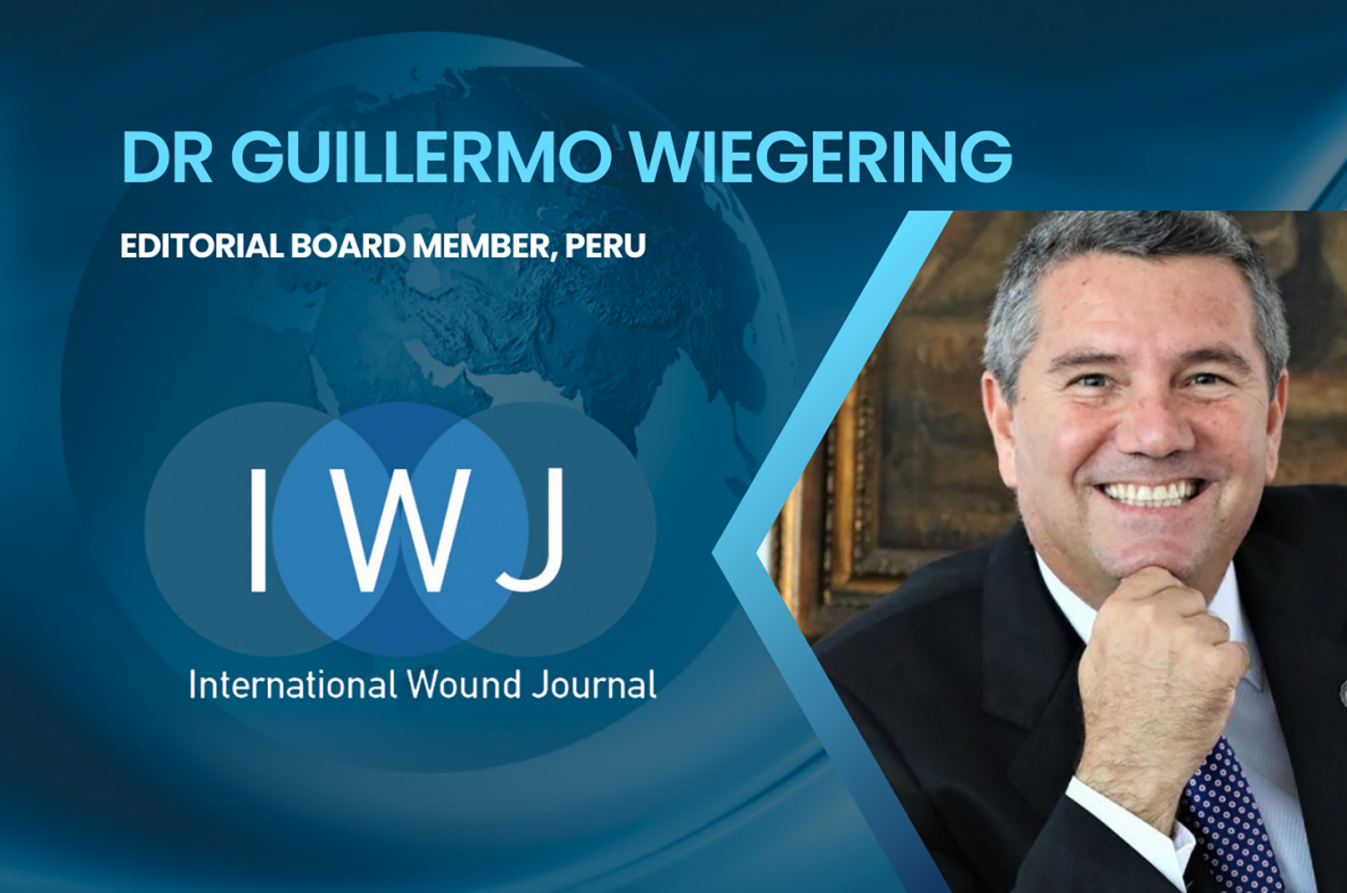
Dr. Guillermo Wiegering, Peru

Dr. Wiegering is a Plastic Reconstructive and Aesthetic Surgeon.

He is a past president of the FELAQ (Ibero‐Latin American Burns Federation). He is also a past president of the Society of Plastic, Reconstructive and Aesthetic Surgery of Peru and a past president of the Burn Society of Peru. He is currently a Full Member of American Society of Plastic Surgeons (ASPS) and a Full Member of International Society of Aesthetic Plastic Surgeons (ISAPS).

He has received a master's degree and a PhD in medicine. He has been a Professor and Tutor of the Chair of Plastic Surgery at the Universities: San Martín de Porres and the San Juan Bautista Past Private University for the past 24 years. He works at the Javier Prado Clinic in Lima in Peru.

**Figure:**
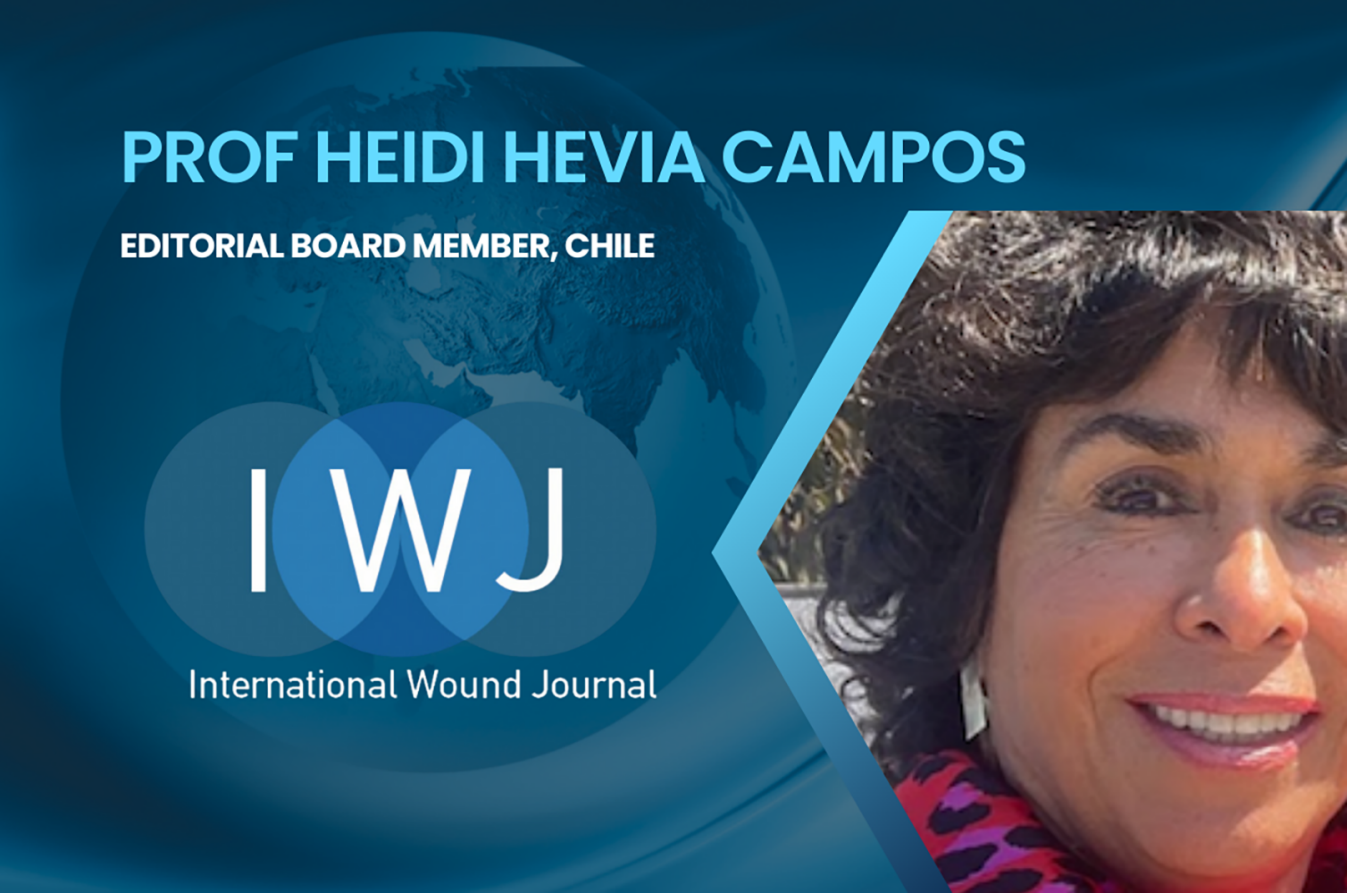
Professor Heidi Hevia Campos, Chile

Professor Campos was born in Heidelberg Germany but finished her nursing studies at the Catholic University of Chile. She started working in a private clinic. Prof. Campos saw the need to provide specialized care for ostomy patients and then did her training as a specialized stomatherapy nurse at Saint Marks Hospital, UK. There she was able to do internships in different hospitals and cities, attending the department of stomatherapy, intensive care and burn units among others. Then, she took an expert program at the Complutense University of Madrid. She returned to Chile full of ideas, but the subject was still unknown in the region. Prof. Campos worked for 5 years in San Borja Arriarán Hospital where she introduced the concept of a nurse specialized in the care of the stoma and wound management. She struggled to develop the concept of comprehensive care in stomas, wounds and continence in Chile. But she persevered and succeeded. She is an active member of the WCET and American Society and participates in the congresses internationally.

Her passion is her patients who motivate her to keep fighting for their wellness. Her current job is at Andres Bello University and she enjoys teaching.

Finally, she believes that a clinician's mission is to work to improve the well‐being of others and alleviate suffering by seeking the best and latest evidence.

**Figure:**
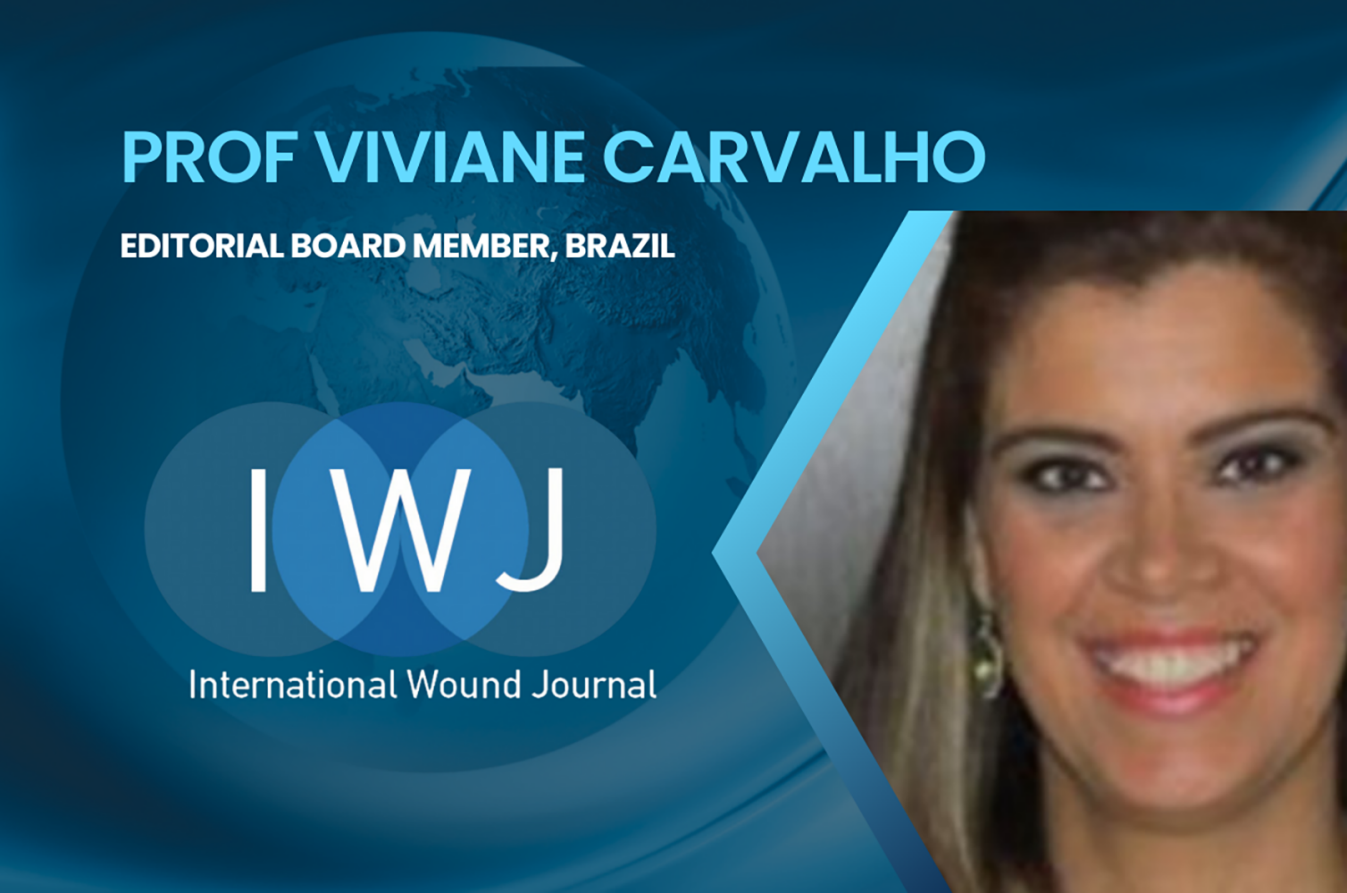
Professor Viviane Carvalho, Brazil

Professor Carvalho is specialized in wound, ostomy and continence care and has a PhD in Medical Science from USP, with 20 years of experience in academic clinical research in wound healing and skincare. She has oriented and graduated 22 master's and one PhD and currently supervises one master's and two PhD students as an Associate Professor of the Graduate Program in Sciences of Guarulhos University.

Since 2007, she has been working with the medical device industry as a consultant in the field of professional training (J&J, Systagenix and Acelity). Prof. Carvalho writes books and scientific papers and serves on editorial boards for major national and international journals in this field.

**Figure:**
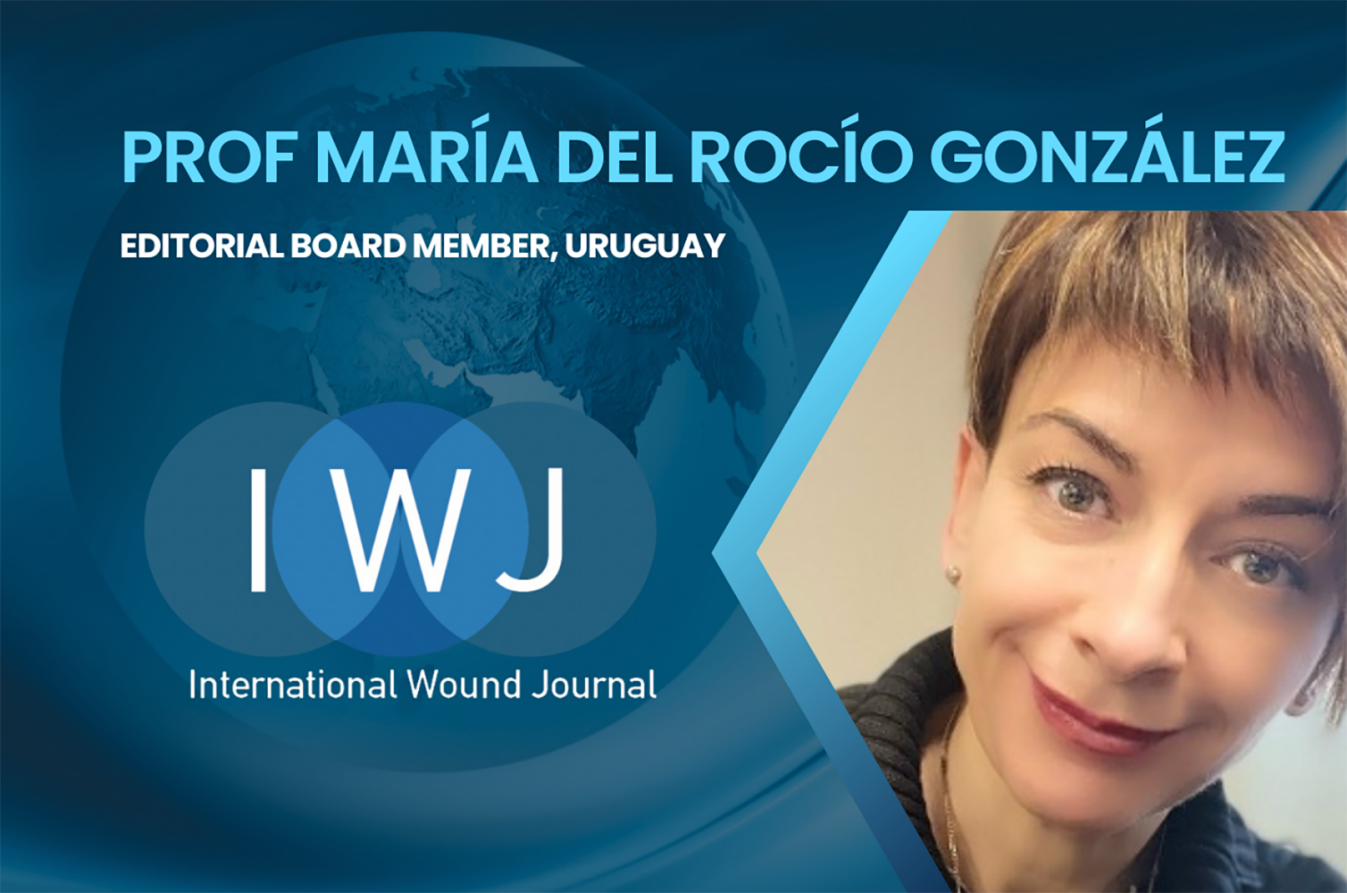
Professor María del Rocío González, Uruguay

Professor González holds a bachelor's degree in nursing, University of the Oriental Republic of Uruguay and a master's degree in comprehensive management and research of chronic wounds, University of Cantabria, Spain. She holds postgraduate qualifications in health administration, patient safety, intensive care nursing and wound management from both local and international universities.

Professor González has numerous professional appointments and has led the specialization of wound care within the region. These include a number of professional bodies: President of the Board of Directors of the Ibero‐Latin American Society of Ulcers and Wounds (2018–2026); President of the Steering Committee of the Uruguayan Scientific Society of Wounds (2018 to present) and Member of the EWMA Teacher Network.

She also holds a number of professional and academic posts: Member of the Department of Quality and Patient Safety; General Directorate of Health Ministry of Public Health, Uruguay (2015 to date); Deputy Director of the Department of Nursing and Coordinator of the Patient Safety Commission of the Catholic Circle of Workers of Uruguay Mutualista (2015 to date); Technical Director of the ‘Santa María’ School of Nursing of the CCOU (2018 to date); Associate Professor at the Faculty of Nursing and Health Sciences of the Catholic University of Uruguay (2015 to present); Assistant Professor Grade II of Health Services Administration

University of the Oriental Republic of Uruguay, Faculty of Nursing (2011 to 2015); Deputy Coordinator of the Patient Safety Nursing Network of Uruguay (RESPU),

Integrated into the International Patient Safety Network PAHO, 2011 to 2013; and Co‐founder of the International Network of Ulcers and Wounds (2012 to date).

**Figure:**
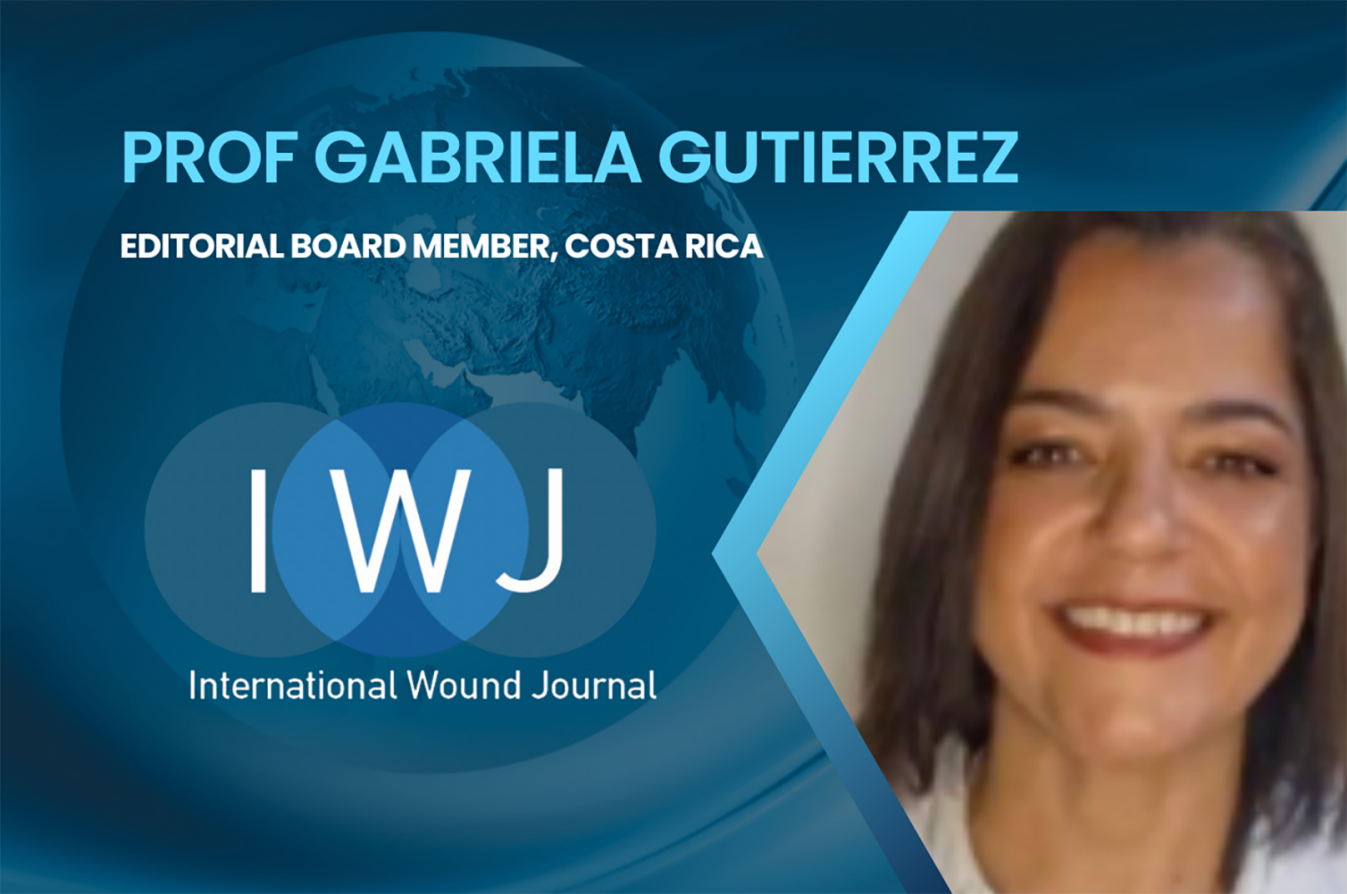
Professor Gabriela Gutierrez, Costa Rica

Gabriela Madrigal Gutiérrez has a Medical Degree in Medicine and Surgery Specializing in Family and Community Medicine. She also has a master's in clinical dermatology and a master's in health services administration. Currently, she is the Assistant Centro Nacional de Rehabilitación, CCSS—San José, Costa Rica; Assistant Clínica Bíblica—San José, Costa Rica and Assistant Centro Médico Momentum Escazú—San José, Costa Rica.

Dr. Gutierrez is also a Professor of Medical Examination Tests, Autonomous University of Central America—San José, Costa Rica. She has certifications in Platelet‐Rich Fibrin Course; Clinical Nutrition Basics Course; Wound Management Course; Stem Cells Course and Stem Cell Extraction and Cultivation Course. Professor Gutierrez is a member of a number of professional associations:

Costa Rican Association of Wounds and Ostomies (ACOHO) (President 2023–2024 Vice President 2017–2023); Latin American Confederation of Wounds, Stomas, and Incontinence (COMLHEI), Secretary 2019–2023 and Co‐Secretary 2023–2024.

**Figure:**
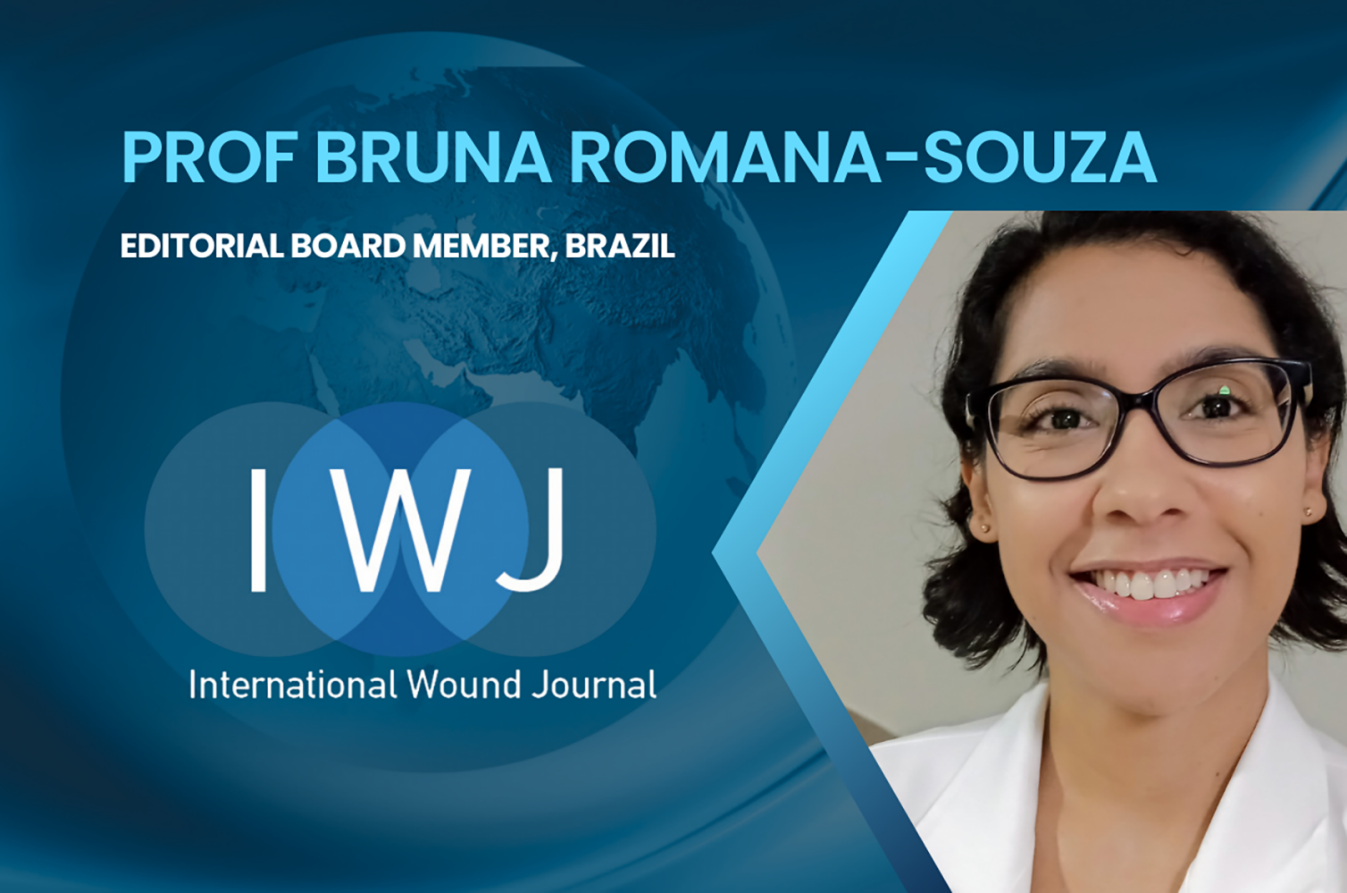
Professor Bruna Romana‐Souza, Brazil

Bruna Romana‐Souza is an Associate Professor of Department of Histology and Embryology at the Rio de Janeiro State University (UERJ) in Brazil, where she received her master's degree and her PhD degree in Experimental Biology. She was a professor and researcher at UERJ from 2012 to present. She teaches ‘Human Histology and Embryology’ for the Bachelor's Degree in Nursing and is a member of the Doctorate Committee of the PhD Program in ‘Experimental and Clinic Physiopathology’. She is a supervisor of bachelor's, master's and doctoral theses. The results of her research have been published in 52 papers in international journals and four chapters in international books (*h*‐index: 18; 875 citations). She has been a referee for ISI journals belonging to top‐decile/quartile in her research area. She is a member of the Editorial Board of *Wound, Repair, and Regeneration, and International Wound Journal*. She was an evaluator of the research activity of the National Council for Scientific and Technological Development and an international referee of the Medical Research Council. She has collaborated with national (Federal University of Rio de Janeiro) and international (University of Illinois Chicago) universities and research centres.

**Figure:**
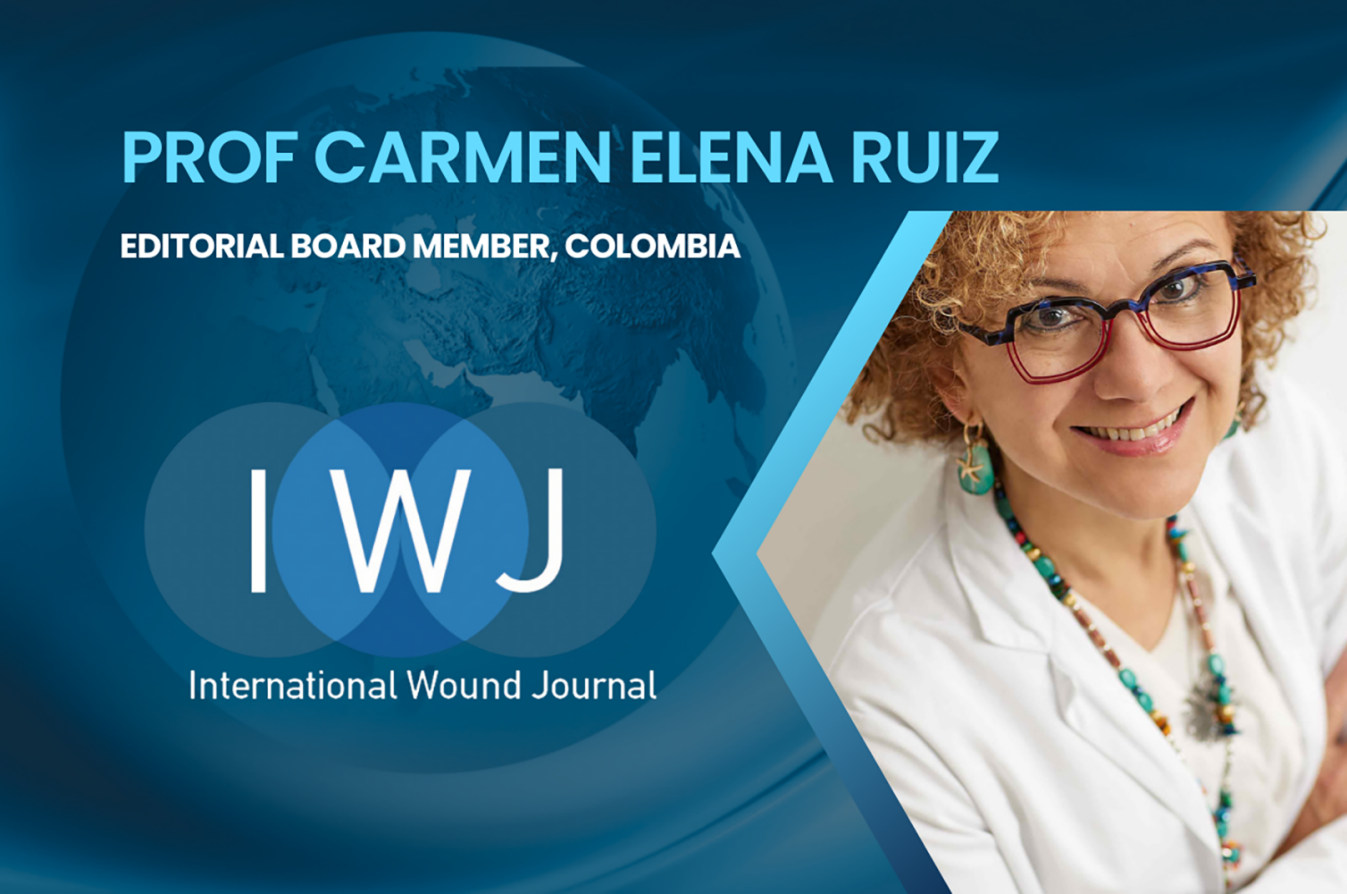
Professor Carmen Elena Ruiz, Colombia

Professor Ruiz trained as a physician and plastic surgeon at the University of Antioquia in Medellin (Colombia), a public university. For personal reasons, she moved to Spain, and after going through several health issues, she decided to continue her profession outside the operating theatre. In the year 2000, during a burns symposium in the city of Bogota (Colombia), she was introduced to the concept of wound healing in a moist environment and began to study and apply the concept.

Later, with the Wound Bed Preparation consensus document of 2003, she thought it was the answer to the management of complex wounds, as it managed how wounds should be treated in a coherent way, and from this time, she began to apply and disseminate advanced wound care. Heridology, as Dr. Harding defined it, in an editorial in the IWJ, is a new specialty. She has worked with acute wounds (traumatic: from anti‐personnel landmine injuries to wounds of labour origin) and chronic wounds or difficult‐to‐heal wounds together with her husband. She has always thought that paradigm shifts are made through education; so in 2017, she developed the Pentagon, as an integrative tool for wound management by uniting the concepts of WBP, TIME, difficult‐to‐heal wounds and wound assessment triangle, which she published with Santiago Roviralta in 2022 and which they have gradually been disseminating in Spanish‐speaking countries.

**Figure:**
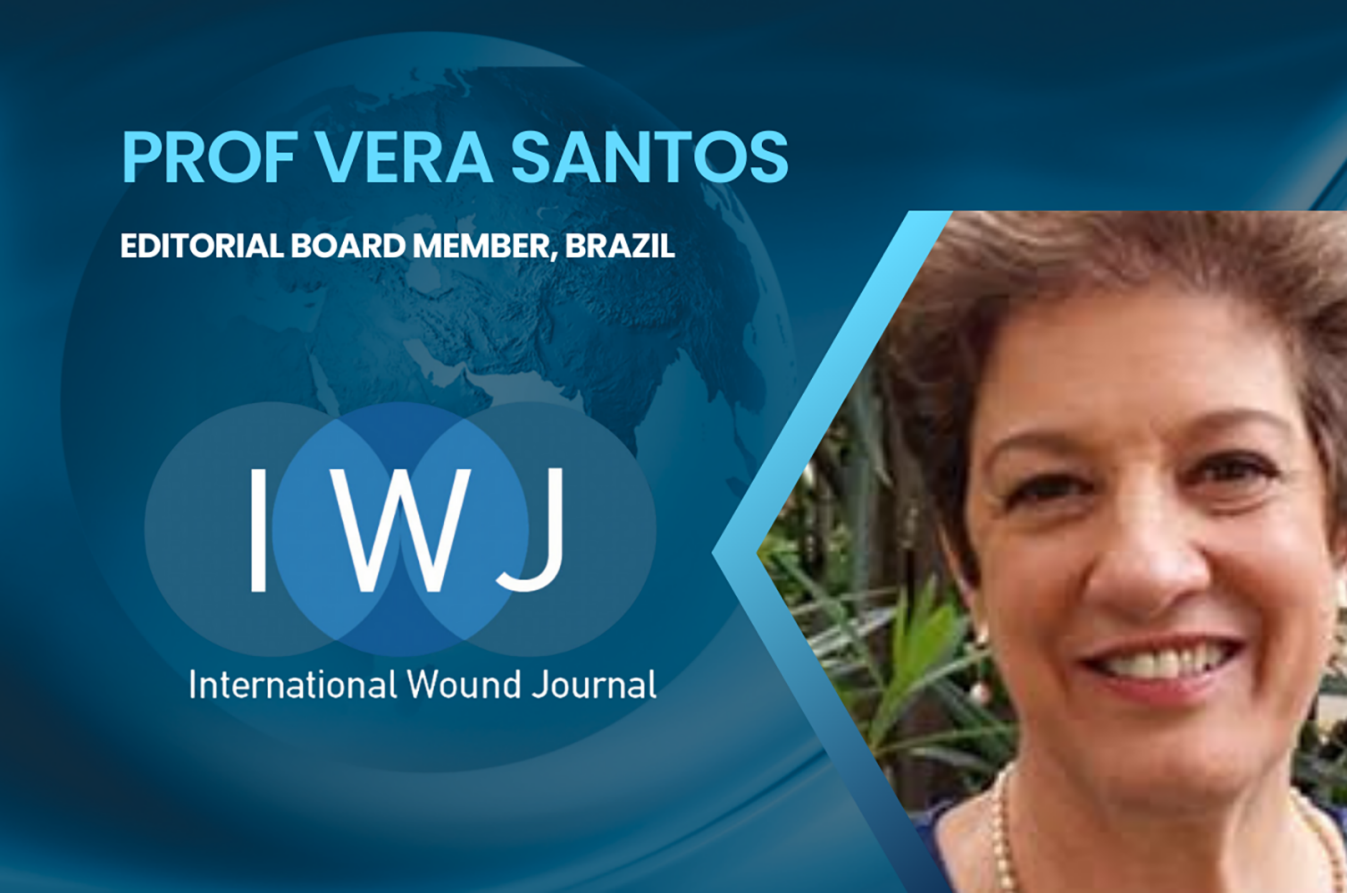
Professor Vera Santos, Brazil

She holds a PhD (1996), a master's degree (1989) and an undergraduate degree in nursing (1976) from the USP School of Nursing—EEUSP. She is a Full Professor at EEUSP after obtaining her Full Professorship in 2006. She is the Head of the Medical‐Surgical Nursing Department (2019–2021). She is a member of the Brazilian Association of Stomatherapy: ostomy, wounds, and Incontinence (SOBEST), of which she is a founding member, having been its president from 1992 to 1997 and from 1999 to 2001, remaining a member of its Scientific Council from 2003 to 2015. Since 2015, she has been elected as Director of SOBEST's International Relations Department. In October 2014, she received the Honorary Title of Specialist Emeritus in Stomatherapy (TiSOBEST Emeritus). She has been a World Council of Enterostomal Therapists (WCET) member since 1992, holding the position of Coordinator of its Education Committee in the 2012–2014 and 2014–2016 administrations. She is also a member of other international associations, such as the International Society For Quality of Life Research (ISOQOL), the Wound Ostomy and Continence Nursing Society (WOCNS) and the International Continence Society (ICS). She holds positions of coordination or international representation in Confederación Multidisciplinar Latinoamericana de Heridas, Estomías e Incontinencias—COMLHEI (Board of Directors of the Scientific Committee, since its foundation in 2017); World Union of Wound Healing Societies—WUWHS (Representative of South America, since 2016) and International Skin Tears Advisory Panel—ISTAP (Regional Director of Latin America 2017–2023). She is a member of the Editorial Board of the *Journal of Wound Ostomy and Continence Nursing and Chronic Wound Care Management and Research* and an ad hoc reviewer for numerous national and international journals. She is the leader of the Stomatherapy Research Group: stomas, acute and chronic wounds and urinary and anal incontinence—GPET, registered with CNPq since 2004. He has participated in developing and revising consensus and best practices in stomas and wounds with SOBEST, ISTAP and the International Wound Infection Institute/Curtin University.

**Figure:**
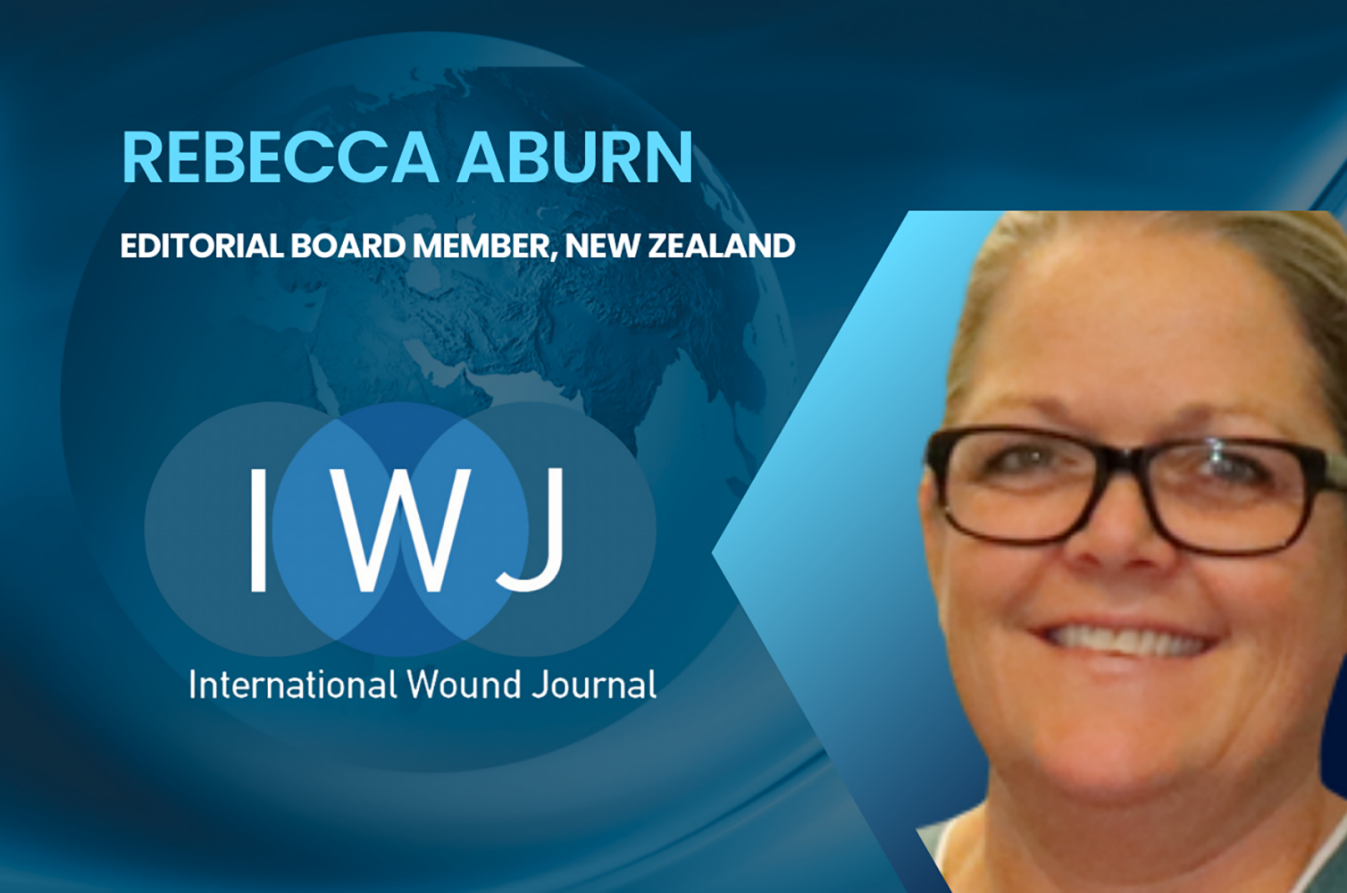
Rebecca Auburn, New Zealand

Rebecca has a 25‐year history of clinical experience in neurosciences, intensive care, vascular, diabetic foot, wound care, clinical education and district nursing. She completed her Master of Nursing in 2006 and has a great passion for nursing practice development. Rebecca is involved at a national and international level in practice and guideline development.

In 2010, Rebecca completed an advanced postgraduate certificate in clinical nursing. In 2017, she completed her Nurse Practitioner training and works as the Nurse Practitioner within the vascular area. Rebecca has presented at both national and international conferences. Her areas of professional interest are innovation to improve a patient's journey, lymphoedema, pyoderma gangrenosum, surgical site infection prevention and care of complex vascular patients.

**Figure:**
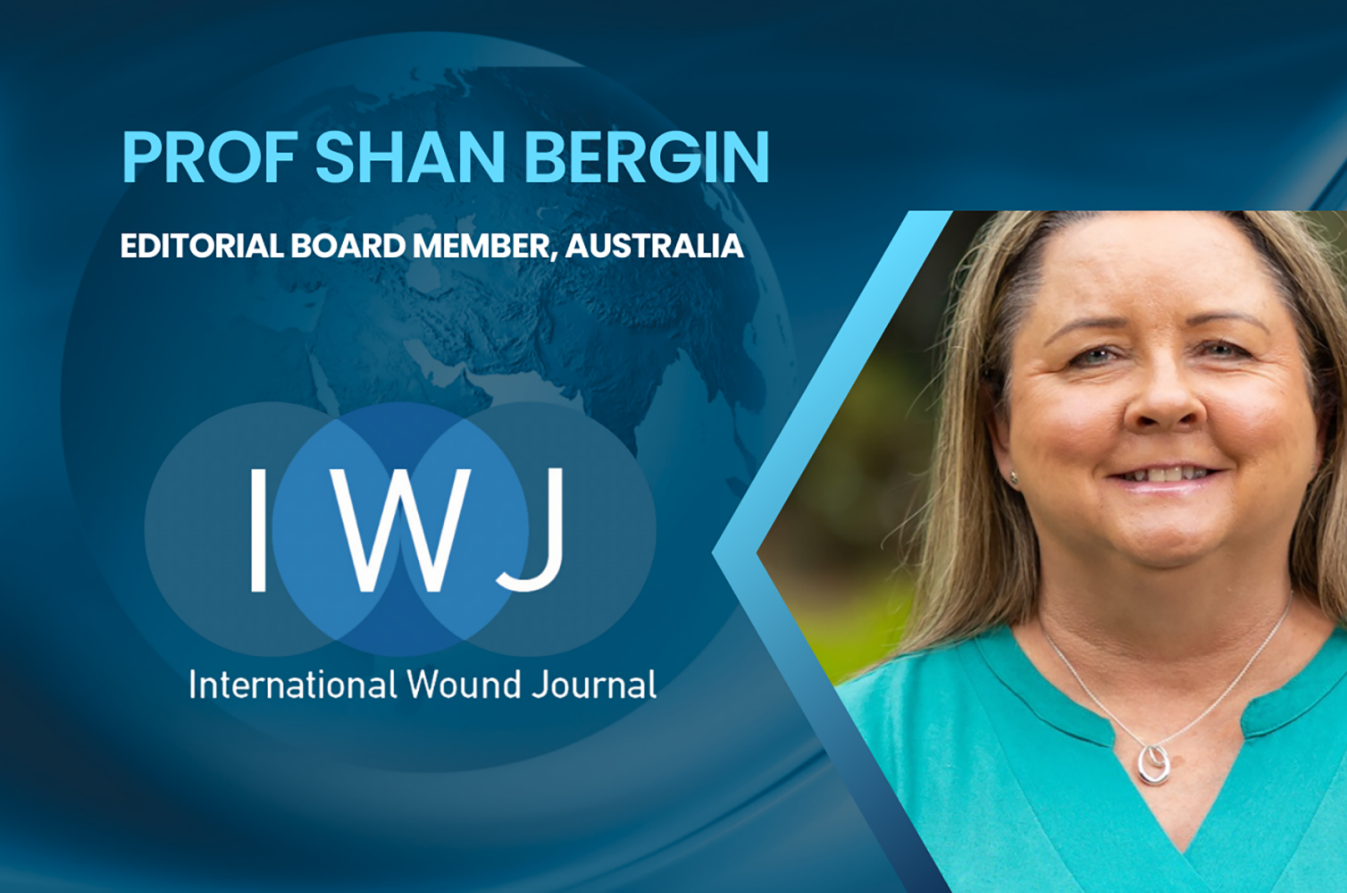
Professor Shan Bergin, Australia

Shan is a Senior Lecturer in the Discipline of Podiatry, School of Allied Health, Human Services and Sport at La Trobe University in Melbourne, Australia. Shan has worked as both a podiatric clinician and researcher and has a particular interest in the high‐risk foot, wound management and the impact of psychosocial factors on clinical outcomes in populations with chronic disease. Shan was awarded a PhD in 2009 where her thesis included a state‐wide prevalence study for diabetes‐related foot complications and an evaluation of community‐based services that were available to this patient cohort. To facilitate her prevalence study, Shan developed and validated a survey tool to collect clinical data. This survey tool (Q‐DFD) has since been used in several international studies. Shan has published over 20 peer‐reviewed journal articles and is currently involved in research related to microbiology of diabetic foot wounds, recurrent and contralateral Charcot's neuroarthropathy and footwear, falls and balance in older adults.

Shan supervises honours, master's and PhD students and is currently working with two master's and one PhD student. Shan also has a keen interest in clinical education and currently works as the Year Level Coordinator for the final year podiatry student cohort and is a subject coordinator for both fourth‐year clinical subjects. Shan has developed an extensive clinical curriculum, including clinical capability assessment tools across years 3 and 4 of the podiatry program and also currently holds the roles of Clinical Education Coordinator, Chair of the Podiatry Clinical Education Committee and Podiatry Placement Coordinator. Shan has taught into a variety of other subjects across all year levels, including research‐focused subjects (1st and 2nd year), clinical assessment and high‐risk foot subjects (3rd year), Foundations for Professional Communication (1st year) and has coordinated the current 4th‐year industry honours program for podiatry. Shan also holds the following professional roles: Chair, Australian Podiatry Education and Research Foundation, President, FootScape Podiatry Charity, member Australian Podiatry Association (APodA), member APodA National Advocacy Advisory Committe, member Wounds Australia and member Editorial Board of *Journal of Foot and Ankle Research*.

**Figure:**
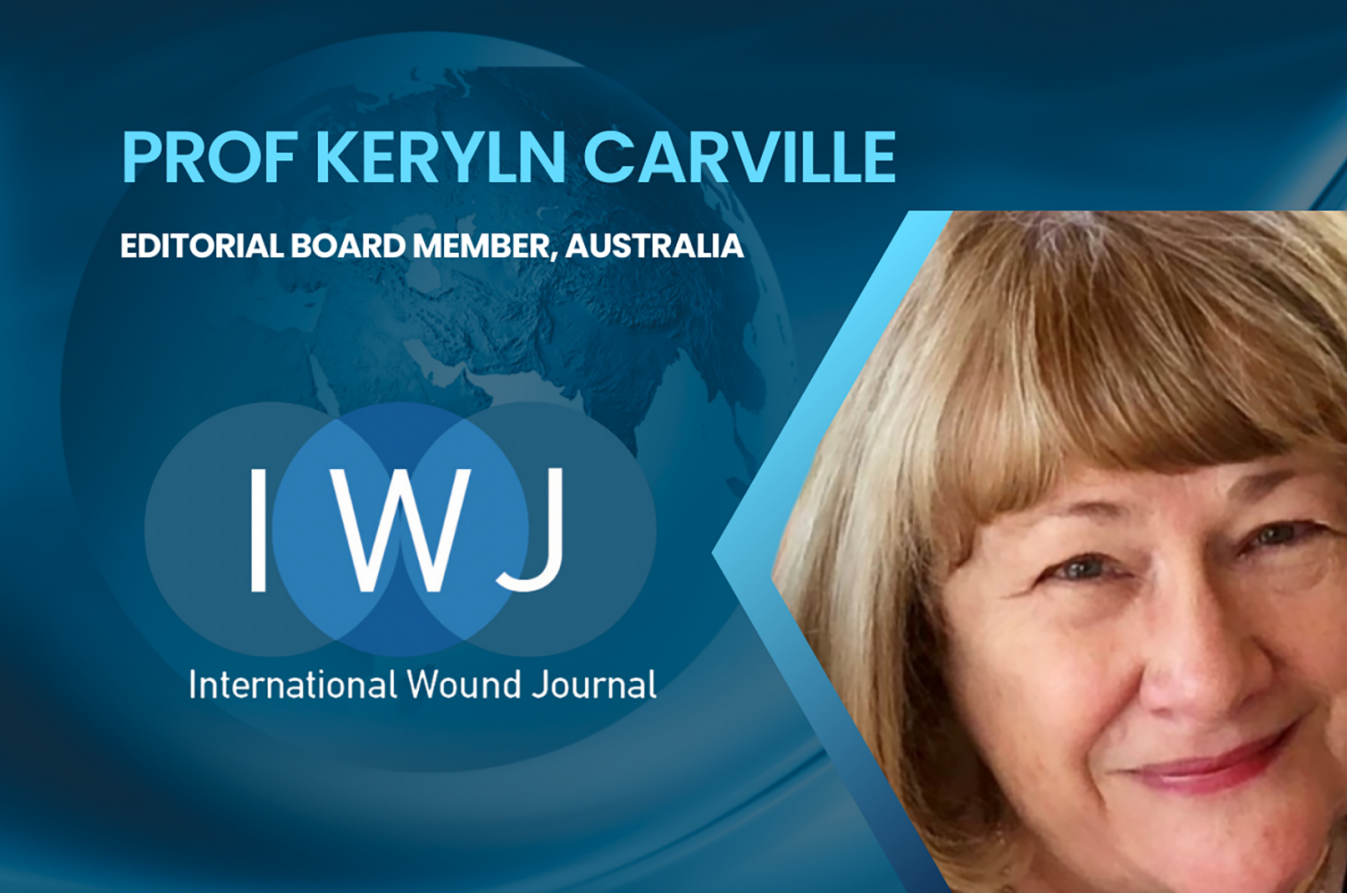
Professor Keryln Carville, Australia

Keryln is the Professor of Primary Health Care and Community Nursing at Silver Chain Group and Curtin University, Western Australia. She is the Deputy Lead of the Curtin Health Innovation Research Institute (CHIRI) Inflammation, Infection and Wounds Domain and Lead of the Wounds Group. She is a lead investigator on WHAM research projects, delivers undergraduate and postgraduate wound curriculum and supervises postgraduate students.

Keryln has extensive clinical experience in wound and ostomy care and is committed to research and education within the domains. Keryln chairs the Pan Pacific Pressure Injury Alliance and was a Guideline Governance Group member for the development of the International Pressure Injury Guideline 2014 and 2019 (and the forthcoming 2025 edition). She co‐ordinates the Curtin University Postgraduate Program of Wound, Ostomy and Continence Practice in Australia and Singapore. She was appointed a Fellow of Wounds Australia in 2006 and a Life Member of the Australian Association of Stomal Therapy Nurses in 2015. She was awarded a World Union of Wound Healing Societies Life Achievement Award in 2022 and the Western Australian Life‐Time Achievement in Nursing Award in 2010. Keryln is a Churchill Fellow 1995. Keryln has over 130 peer‐reviewed publications and texts.

Keryln's research interests include the prevention and management of acute and chronic wounds, the promotion of recovery and rehabilitation of persons with an ostomy and the development of standards, guidelines and evidence summaries. She has a particular interest in advancing best practices in wound and ostomy care in low‐resource countries.

**Figure:**
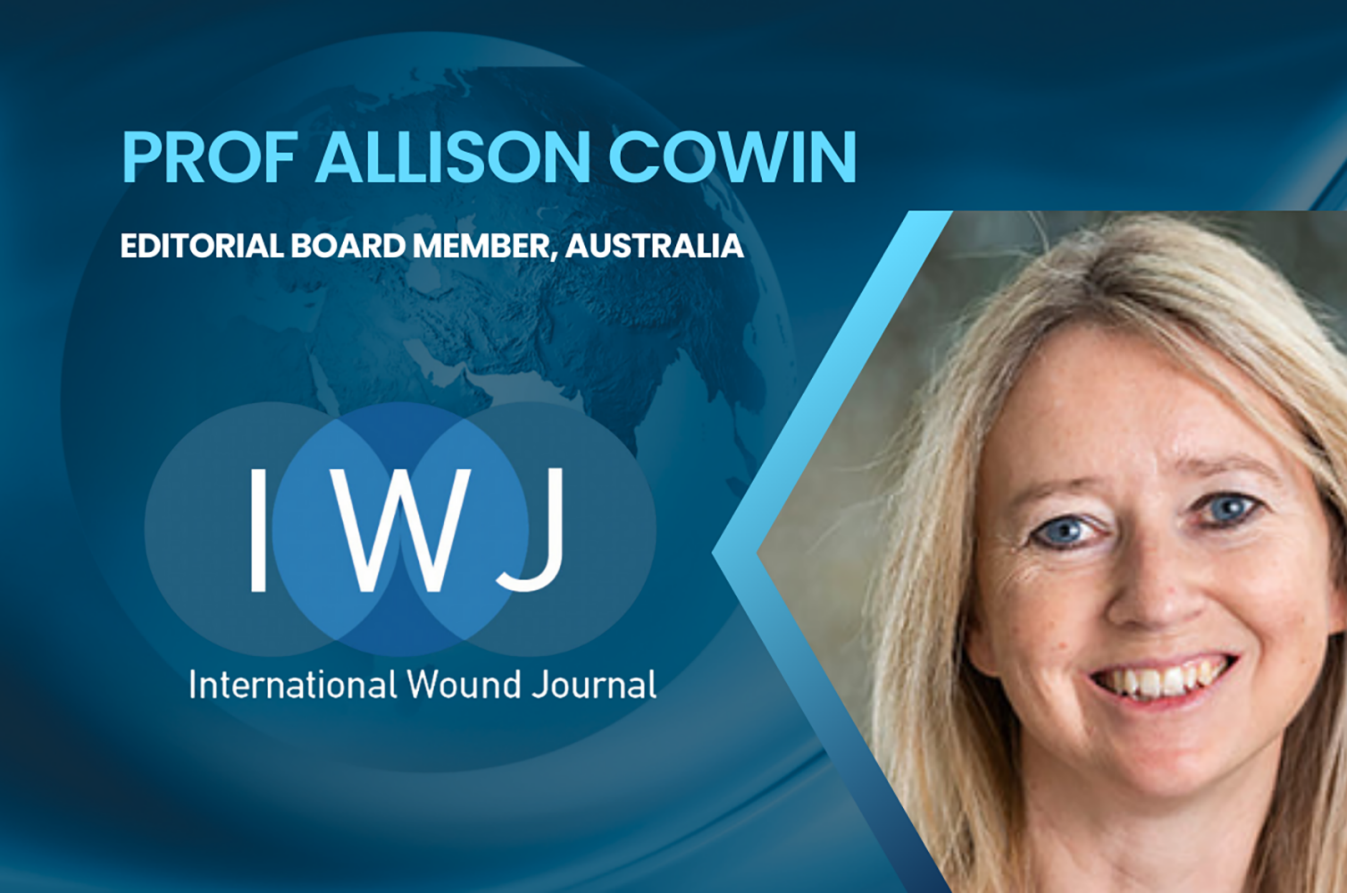
Professor Allison Cowin, Australia

Professor Allison Cowin is Deputy Director of the Future Industries Institute and Professor of Regenerative Medicine at UniSA. She is a leader in wound healing, internationally renowned for her work on the cytoskeletal protein flightless I and how it negatively impacts tissue repair. She leads a group of 12 research scientists and students investigating all aspects of wound healing and regenerative biology. She has been awarded over $20M in grants, including from the US Department of Defence and continuous NHMRC funding since 2004 for her work developing antibody technologies for the treatment of wounds. Her research has received special recognition from the NHMRC by inclusion in its ‘10 of the best projects’ publication. She has been awarded five independent fellowships (three from the NHMRC); she won the Women in Innovation: Science award (2016) and was an SA finalist in the Telstra Women's Business awards in 2015, and her research ‘Novel drug delivery of therapeutic antibodies to wounds’ made the finals of the ‘Australian Innovation Challenge’ (2015). Prof. Cowin is the Co‐Chair of the National AHTA Wound Research Initiative and is the Editor of the Australian journal Wound Practice and Research. Prof. Cowin was the founder and inaugural president (2007–2012) of the Australasian Wound and Tissue Repair Society and is now treasurer of this organization. She is currently President Elect and Secretary of the Australasian Society for Dermatology Research.

**Figure:**
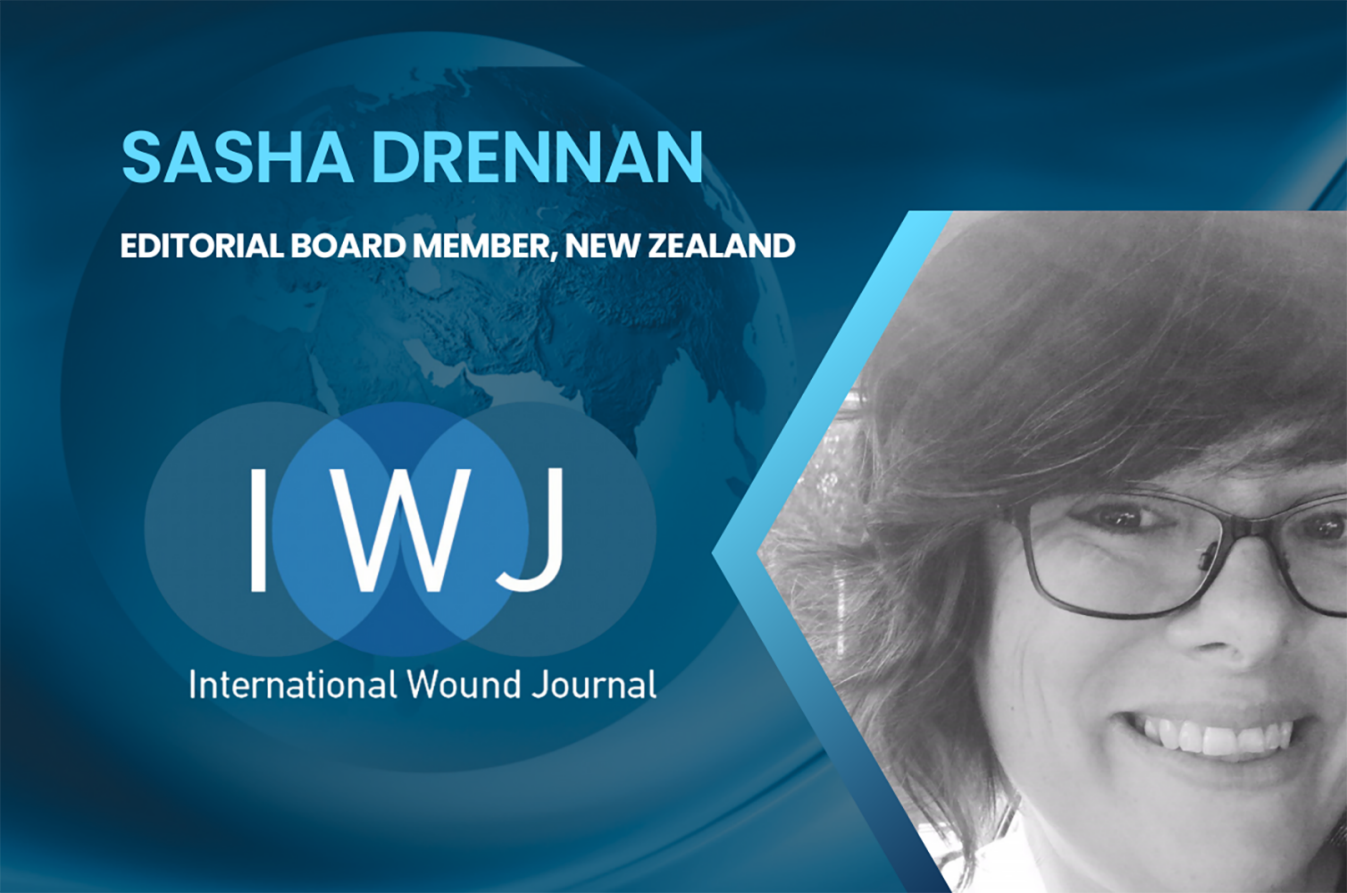
Sasha Drennan, New Zealand

Sasha has 20+ years of clinical experience in both the UK and NZ in several areas of nursing, including perioperative care, plastics, general, breast and ENT surgery, district nursing and clinical education.

She is presently completing her Master's in Digital Health and has a passion for nursing development that encourages the best patient care and is vocal in her belief of how digital education and innovation can create care equity.

Having gained postgraduate qualifications in nurse education, perioperative care and palliation in the UK and emigrating to NZ in 2010, she has continued her postgraduate journey to ensure evidence‐based care.

**Figure:**
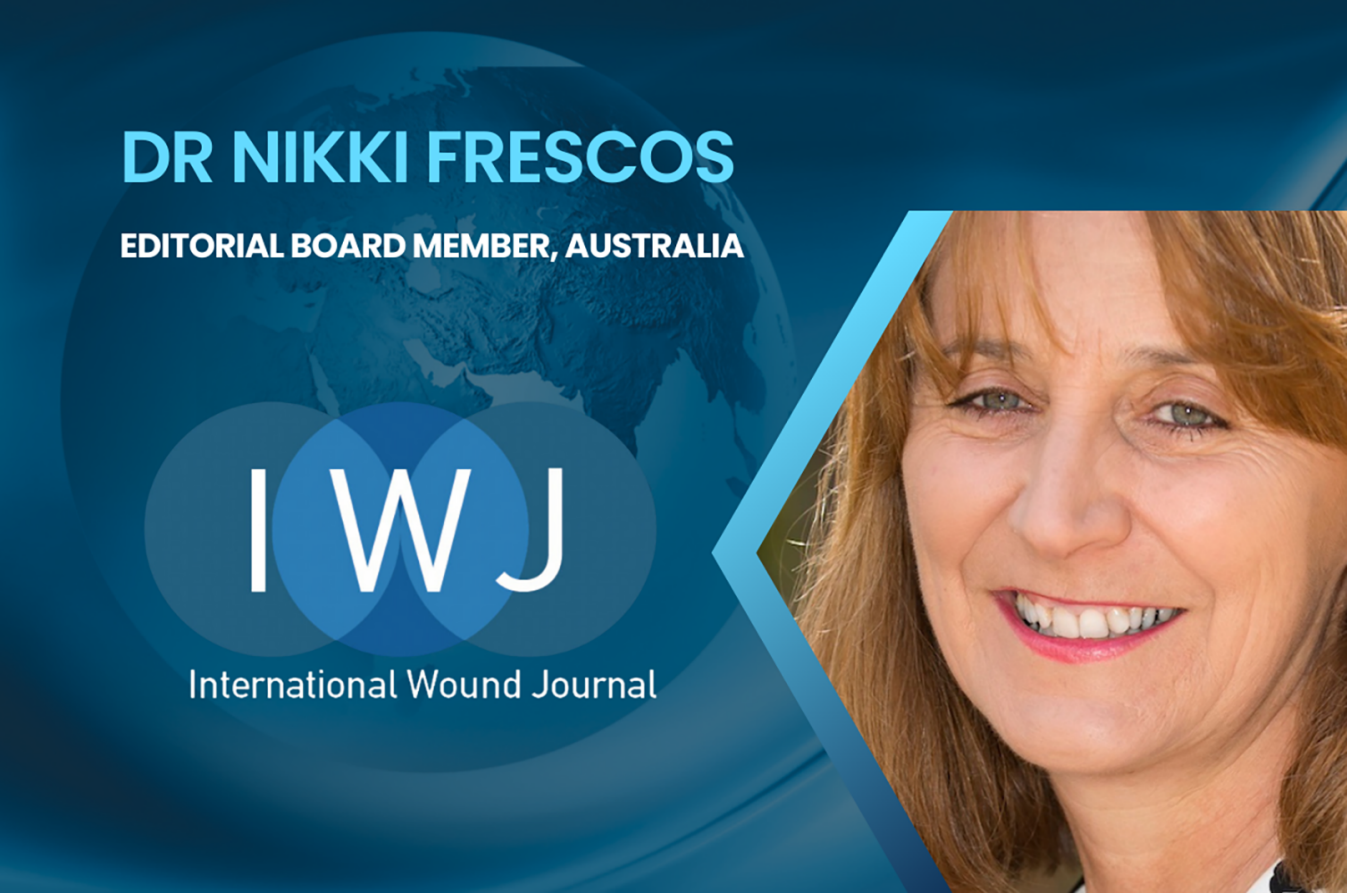
Dr. Nikki Frescos, Australia

Dr. Nicoletta Frescos is a lecturer (teaching and research) in the discipline of podiatry and has more than 20 years of clinical, teaching and research experience. In addition, she is also the clinical trials coordinator for wound care at Austin Health. Nikki is an Australian representative for the Western Pacific Region branch of D‐Foot International and vice president of the Asia Pacific Diabetes Limb Prevention Association.

**Figure:**
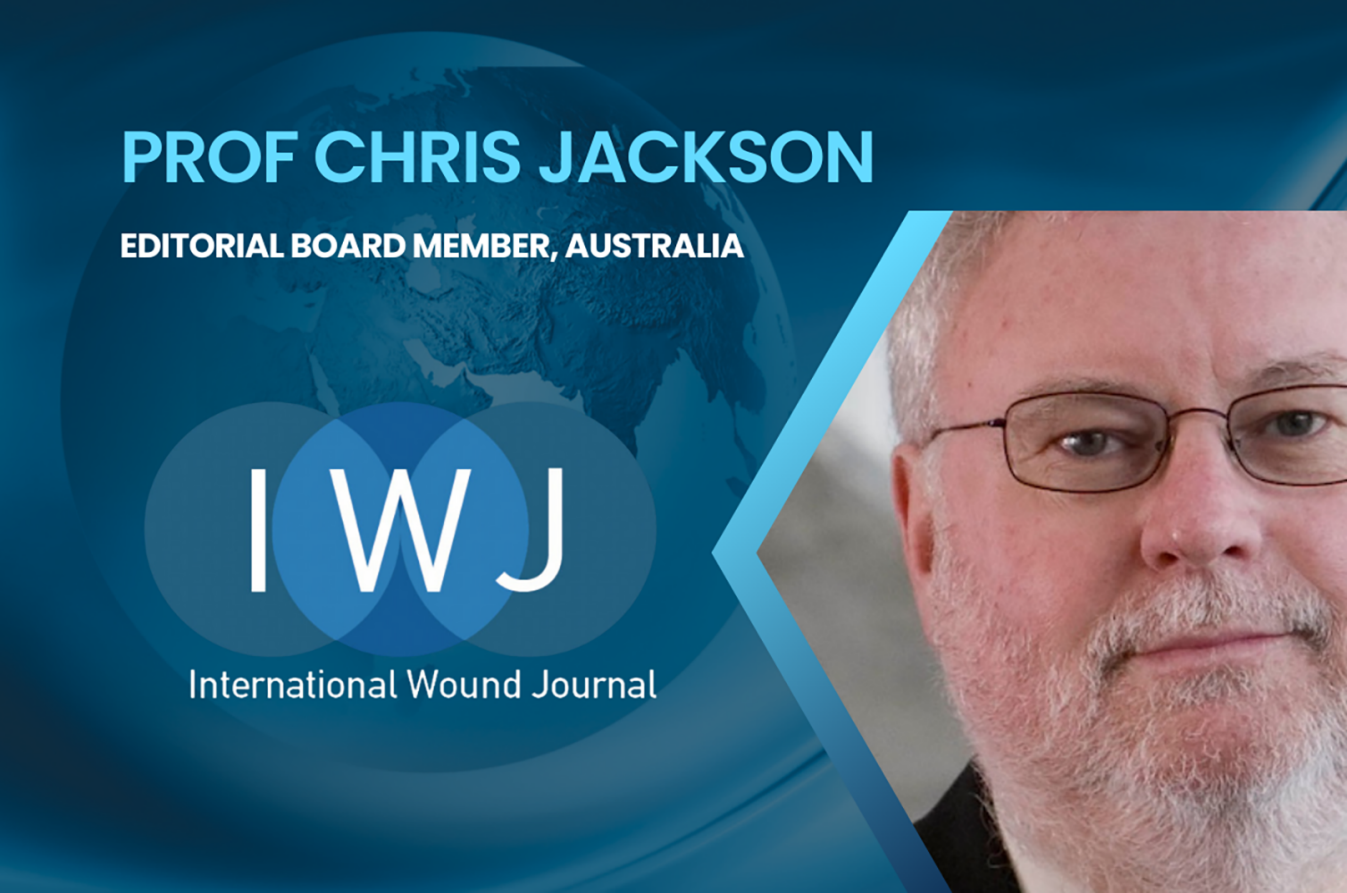
Professor Chris Jackson, Australia

Professor Jackson is the Hon. Professor of Regenerative Science and Medicine, Kolling Institute, Northern Clinical School, Faculty of Medicine and Health, University of Sydney (2021 to present).

Previously, he was the Professor of Regenerative Science and Medicine, NCS, University of Sydney (2013–2020) and the Associate Professor, NCS, University of Sydney (2006–2013); he has a long relationship with the University of Sydney. Professor Jackson completed his PhD from the University of Sydney.

He was the Director, Sutton Arthritis Research Laboratory, Royal North Shore Hospital (1999–2020).

Professor Jackson has received grants awards as Chief Investigator >$16M, including NHMRC; Arthritis Australia; Juvenile Diabetes Research Foundation International; Health Research Council NZ; Korea National Research Foundation; Lincoln Centre; Ramsay Health.

His research and that of his team focus on autoimmune/inflammatory conditions, particularly inflammatory skin conditions and rheumatoid arthritis. His lab's research has evolved to become the true bench to bedside, arising from his discovery of the regenerative capacity of a physiological protein, activated protein C (APC). Positive results from basic experimental models using novel biochemical and molecular techniques in the Sutton lab allowed him to progress into proof of principle experiments to reveal that APC not only has potent anti‐inflammatory actions but also heals wounds in preclinical models. He then linked with his clinical colleagues at RNSH to translate the positive laboratory findings into human clinical trials for wound healing. To date, >50 patients with various types of recalcitrant chronic wounds who have been treated with APC have all responded positively, most with complete healing. A large trial in diabetic ulcers is now being established by our commercial partner ZZ‐Biotech (www.zzbiotech.com). In collaboration with Prof. Griffin (SCRIPPS, San Diego) and Jay Hennock (CEO), CJ recently co‐founded a new Biotech company, Novapep (www.novapep.com), currently elucidating the efficacy and safety of novel peptides that mimic the action of APC.

**Figure:**
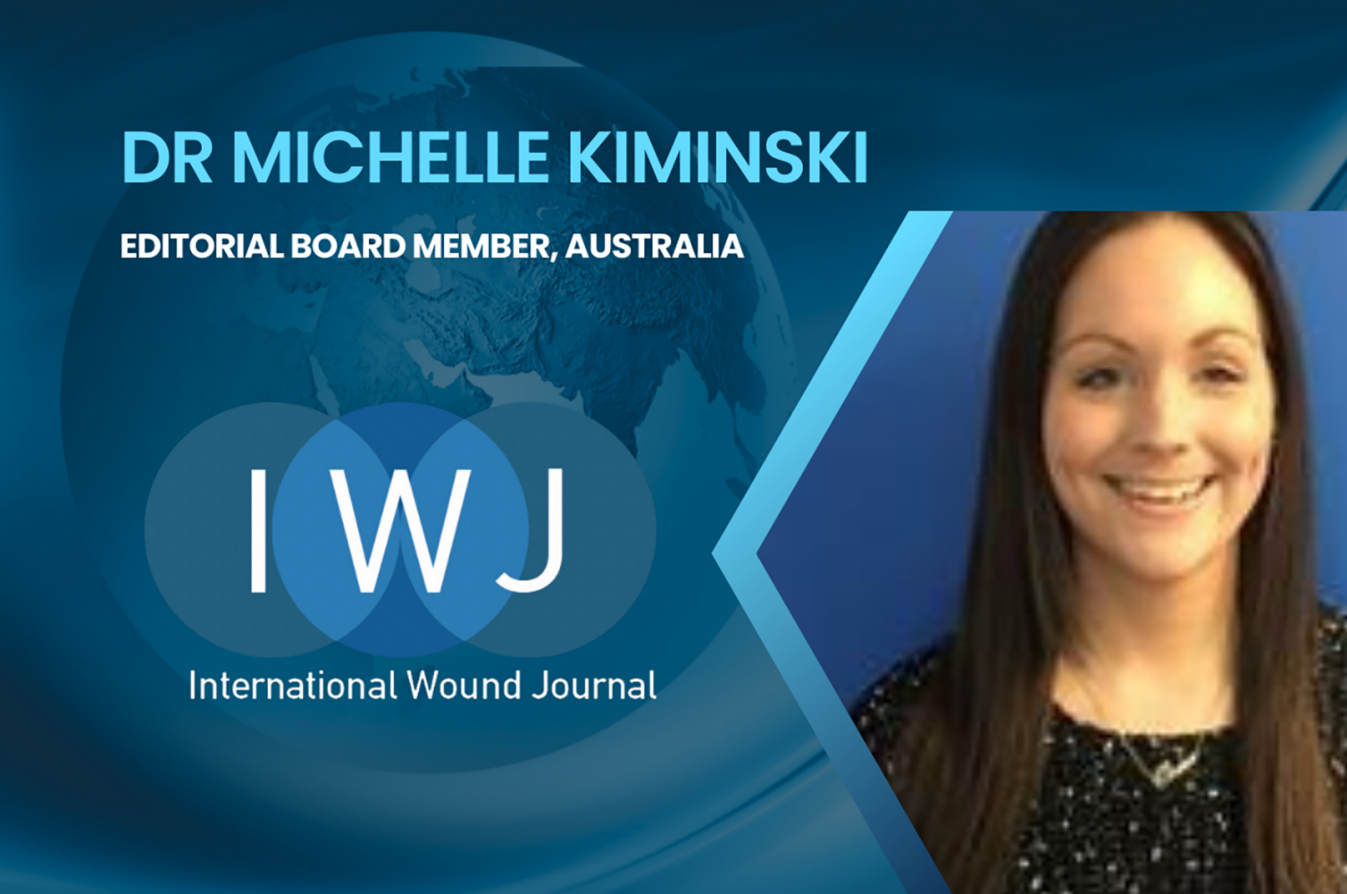
Dr. Michelle Kiminski, Australia

Dr. Michelle Kaminski is an Adjunct Lecturer in the Discipline of Podiatry at La Trobe University, with a particular interest in the prevention and management of foot ulceration in high‐risk populations. In addition, Michelle is the Podiatry Research Lead at Monash Health and holds an Adjunct Senior Research Fellow position at Monash University. Michelle has experience in leading several large research projects within the public health and university sectors and is passionate about embedding research into clinical practice and providing mentoring to emerging clinician‐researchers. Michelle is currently supervising one PhD student. Michelle's expertise is recognized by her current appointment as an editorial board member for the *Journal of Wound Management*, the official journal of the European Wound Management Association. Michelle is also a member of the Asia Pacific Diabetic Foot Ulcer Academics Club, where she is involved in conducting international research studies with leading experts in the field. A career highlight was an invitation by Diabetes Feet Australia to be a chapter group member for developing the new Australian diabetes‐related foot disease guidelines, where she was secretary and first author of the Prevention Guideline. Michelle has published 18 peer‐reviewed publications (11 as first/senior author, one book chapter and one national guideline). Her papers have been cited 426 times in the scientific literature, which equates to a Google Scholar h‐index of 11 and an i10‐index of 12. Michelle is currently involved in several research projects, including investigations related to the prevention and management of foot ulceration in high‐risk populations, the burden and impact of diabetes‐related foot disease, musculoskeletal disorders of the foot and ankle and student motivators and barriers for studying podiatry. Michelle proudly acknowledges the Wurundjeri people of the Kulin nations as the Traditional Custodians of the land and its waterways on which she lives and works.

**Figure:**
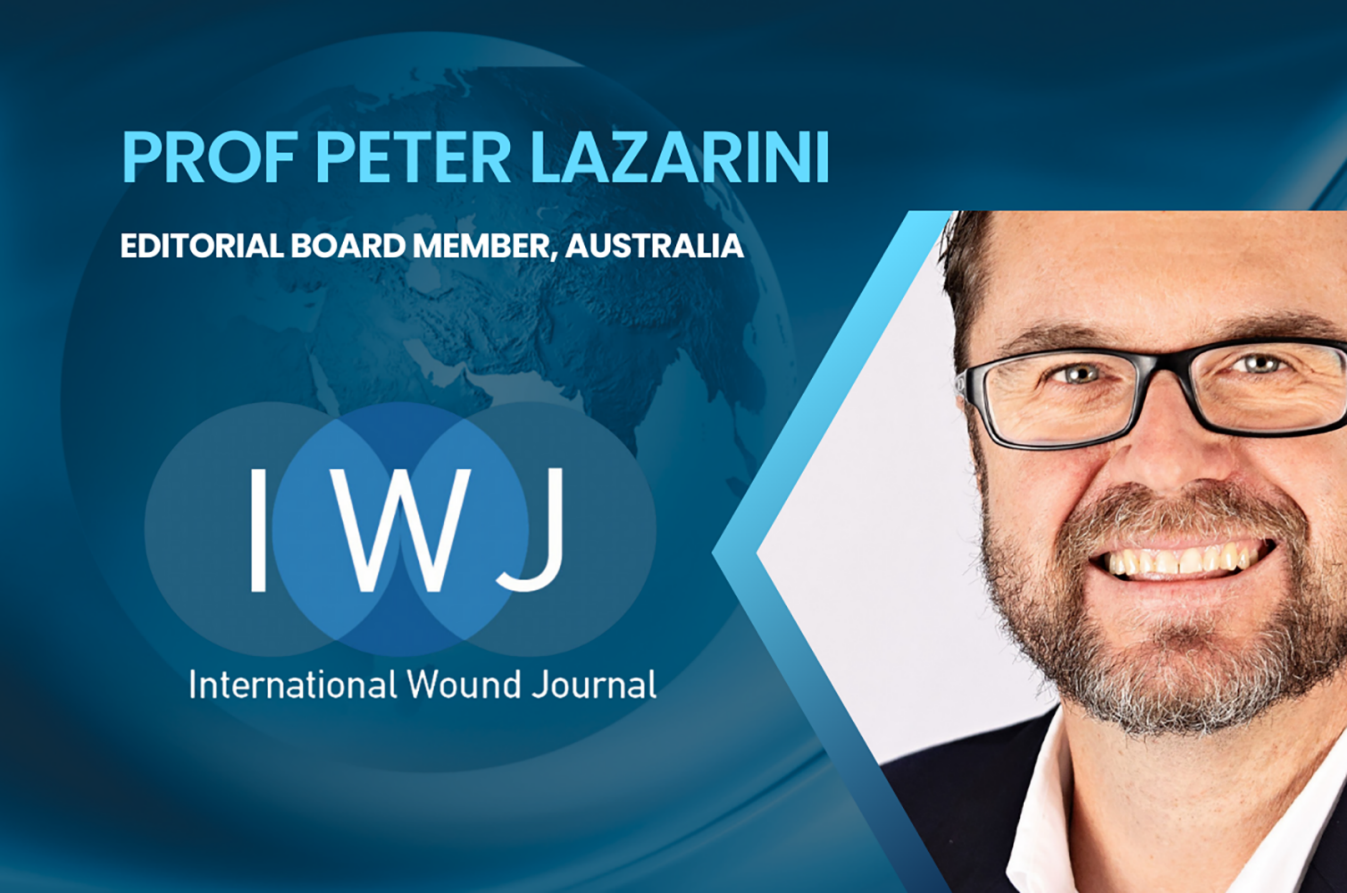
Professor Peter Lazzarini, Australia

Peter's career has been motivated by the goal of ending avoidable amputations within a generation for people with diabetes foot disease. Prior to his research career, he was a nationally regarded clinical podiatrist with >10 years of foot disease experience. In 2011, he commenced his research career by establishing Australia's first diabetes foot disease research program at QUT/MNNHS; he also began his PhD to investigate the burden of foot disease in inpatient populations. He completed his PhD in 2016 and was awarded an NHMRC Early Career Fellowship in 2018, an Associate Professor in Foot Disease at QUT in 2020 and an International Rising Star Award for important international research contributions to the field at the International Symposium on the Diabetic Foot in 2023. Today, he is the Principal Research Fellow leading the #7 leading diabetes foot disease research program in the world at QUT/ MNHHS, a program that is producing paradigm‐changing new global knowledge and health impacts in three main diabetes foot disease areas: quantifying disease burdens, identifying key risk factors and developing novel treatments.

Since completing his PhD in 2016, he has published >80 manuscripts, including 69% in Q1 journals (notably there are no Q1 foot journals), 58% as first/senior author, 55% cited in guidelines/policies, 36% in the top 10% most cited in the field, and his FWCI since is 3.6. Of note, he was the first/senior author on the most highly cited 2018 and 2020 original papers in the field, co‐senior author on a paper awarded a top published study in the past 4 years in the field, and otherwise, he has been the first/senior author on nine of 16 papers published in Decile 1 journals in the diabetes and endocrinology field.

Furthermore, Peter has been the first/senior author on multiple prestigious international guideline and policy papers, including the 2023 and 2019 International Working Group on the Diabetic Foot Offloading Treatment Guidelines, 2021 Australian Evidence‐based guidelines for Diabetes‐Related Foot Disease and 2018 Australian Diabetes‐Related Foot Disease Strategy, and he was one of only 14 leading international diabetes foot disease expert authors (one of two ECRs) invited to co‐author the prestigious 2023 and 2019 International (IWGDF) Diabetic Foot Definition Standards Guideline.

**Figure:**
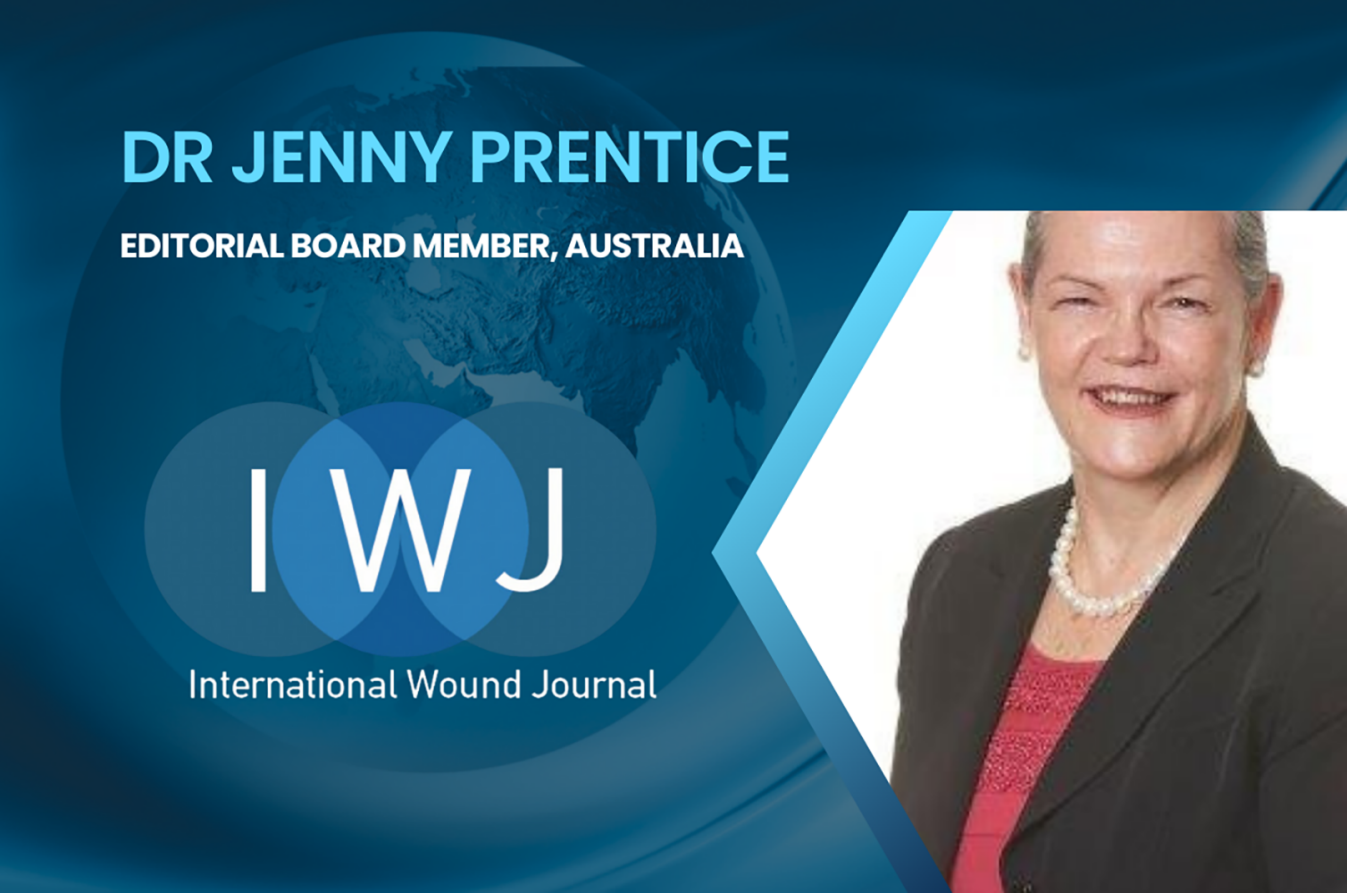
Dr. Jenny Prentice, Australia

Dr. Jenny Prentice has over 40 years' experience in nursing, stomal therapy and wound management in acute, community, private and aged care sectors in Australia and New Zealand. Jenny is an inaugural fellow and past president of Wounds Australia (formerly the Australian Wound Management Association). In addition, Jenny was the Founding Editor in 1993 of Wounds Australia's *Journal Wound Practice & Research* (formerly *Primary Intention*). She remains on the Editorial Board and is also an Editorial Board member of *Advances in Skin & Wound Care*. As a member of the National Medical Benefit Scheme Taskforce on Wound Management, Jenny contributed to a report to the Federal Government on the status of wound management in Australia and the need for dressing reimbursement in 2020. In addition, Jenny was a member of the International Ostomy Guideline Development Group 2020 for the World Council of Enterostomal Therapists and a Committee Member of Diabetic Feet Australia who adapted the International Working Group on the Diabetic Foot (IWGDF) Guidelines' practical guidelines on the prevention and management of diabetic foot disease to the Australian context in 2021.

Currently, Jenny works for an aged care provider in addition to providing independent consultancy in wound skin and ostomy care nationally and internationally.

**Figure:**
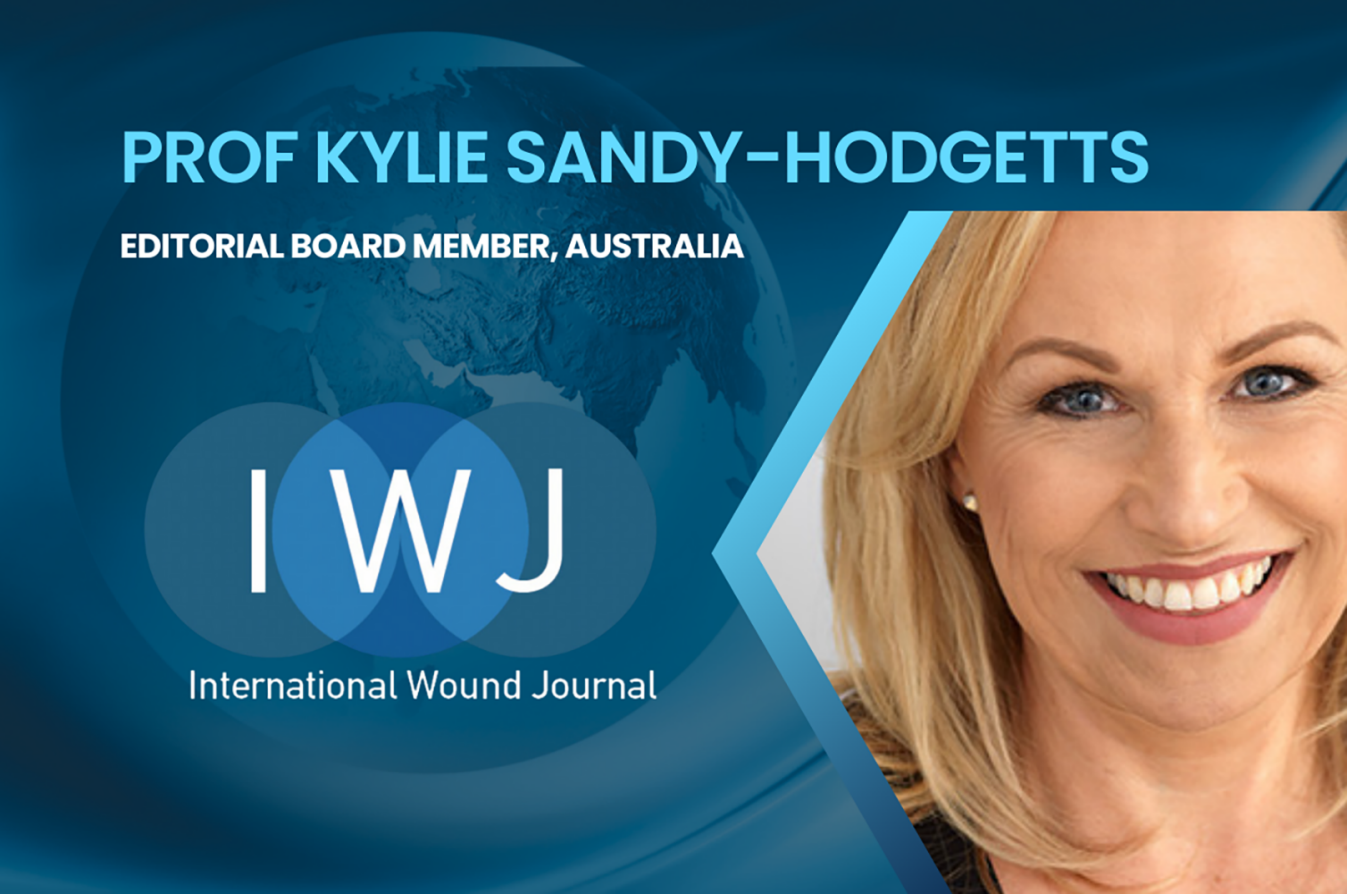
Professor Kylie Sandy‐Hodgetts, Australia

Kylie is an Associate Professor, Lead Skin Integrity Research Group at the Centre for Molecular Medicine & Innovative Therapeutics, Murdoch University. She is also an Honorary Senior Lecturer, the School of Medicine, Cardiff University and Adjunct Senior Research Fellow, School of Biomedical Sciences, University of Western Australia. She is the Founder and President of a not‐for‐profit association, the International Surgical Wound Complications Advisory Panel (ISWCAP). ISWCAP's vision is to generate awareness of surgical wound complications and improve outcomes for patients through early detection and prevention via education and research with an international panel of experts. Kylie is a Research Scientist and Chief Investigator of several clinical trials ranging from Phase 1 first‐in‐human studies to phase 3–4 comparative effectiveness trials. Kylie's research focuses upon early identification, prevention and management of surgical wound complications, including surgical site infection (SSI) and surgical wound dehiscence (SWD). Kylie has chaired and co‐authored over five international clinical consensus documents, first authored over 20 original research papers and chaired the ISWCAP Best Practice Statement on the early identification and prevention of surgical wound complications. She is a regular reviewer for many peer‐reviewed journals and sits on a number of journal editorial boards.

She is a Past Chair of the Board of Wounds Australia and currently serves on a number of national and international boards, including the World Union of Wound Healing Societies Executive Board as Recorder and the Scientific and Ethics Committee.

Kylie is a past Chair of the Australian National Steering Committee Surgical Wounds Panel and Wound Research Directory for the Australian Health Research Alliance National Wound Care Initiative (2020–2023). She was recently acknowledged internationally for her contribution to the field of surgical site infection prevention as the 2021 Winner of the Journal of Wound Care World Union Innovation in Surgical Site Infection Award.

**Figure:**
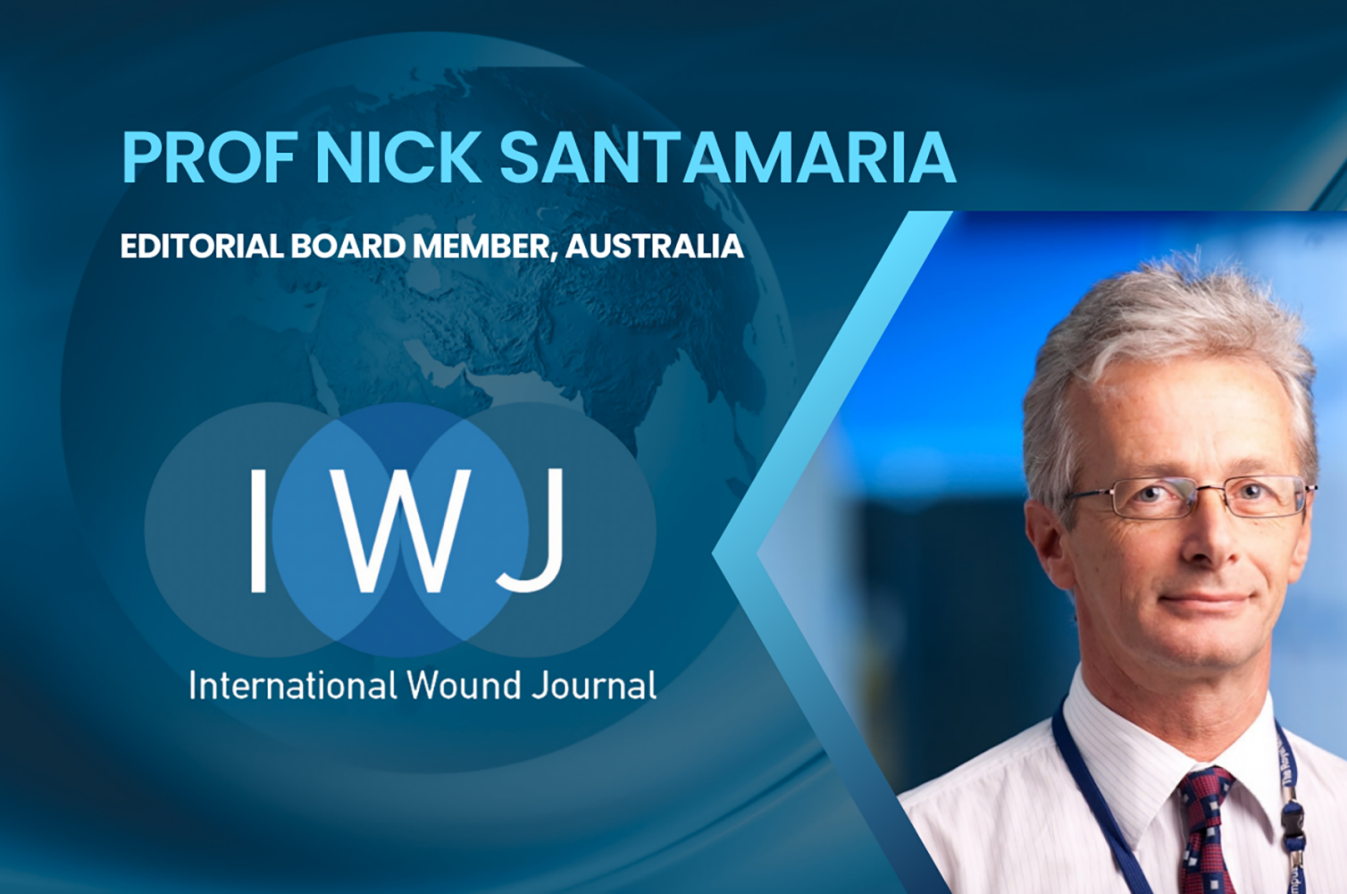
Professor Nick Santamaria, Australia

Professor Santamaria is Professor of Nursing Research, Translational Research, Faculty of Medicine, Dentistry & Health Sciences, University of Melbourne (Honorary) and a Visiting Professor, Faculty of Medicine Cardiff University, Wales, United Kingdom (2019–2023).

His qualifications include RN, RPN, BAppSc (AdvNsg) (LaTrobe), MEdSt (Monash), GradDip Health Ed (Deakin) and a PhD (Melbourne).

Professor Santamaria was the inaugural Professor of Translational Nursing Research and Director of Research at the Royal Melbourne Hospital and The University of Melbourne between 2009 and 2017, and prior to that, he was appointed as Professor of Ambulatory Care at Royal Perth Hospital and Curtin University in WA. He has a long history in wound research and has published more than 160 peer‐reviewed articles and a number of book chapters. He has secured $13 million in research funding and has conducted many clinical trials in both Australia and internationally. He is a member of the Global Advisory Board on Pressure Injuries for Molnlycke AB, Sweden, where he consults on the development of novel wound dressing products and pressure injury prevention techniques. He has supervised the completion of a number of master's and PhD students. His current wound research projects involve collaborations with the USA, UK, Sweden, Italy and Portugal.

**Figure:**
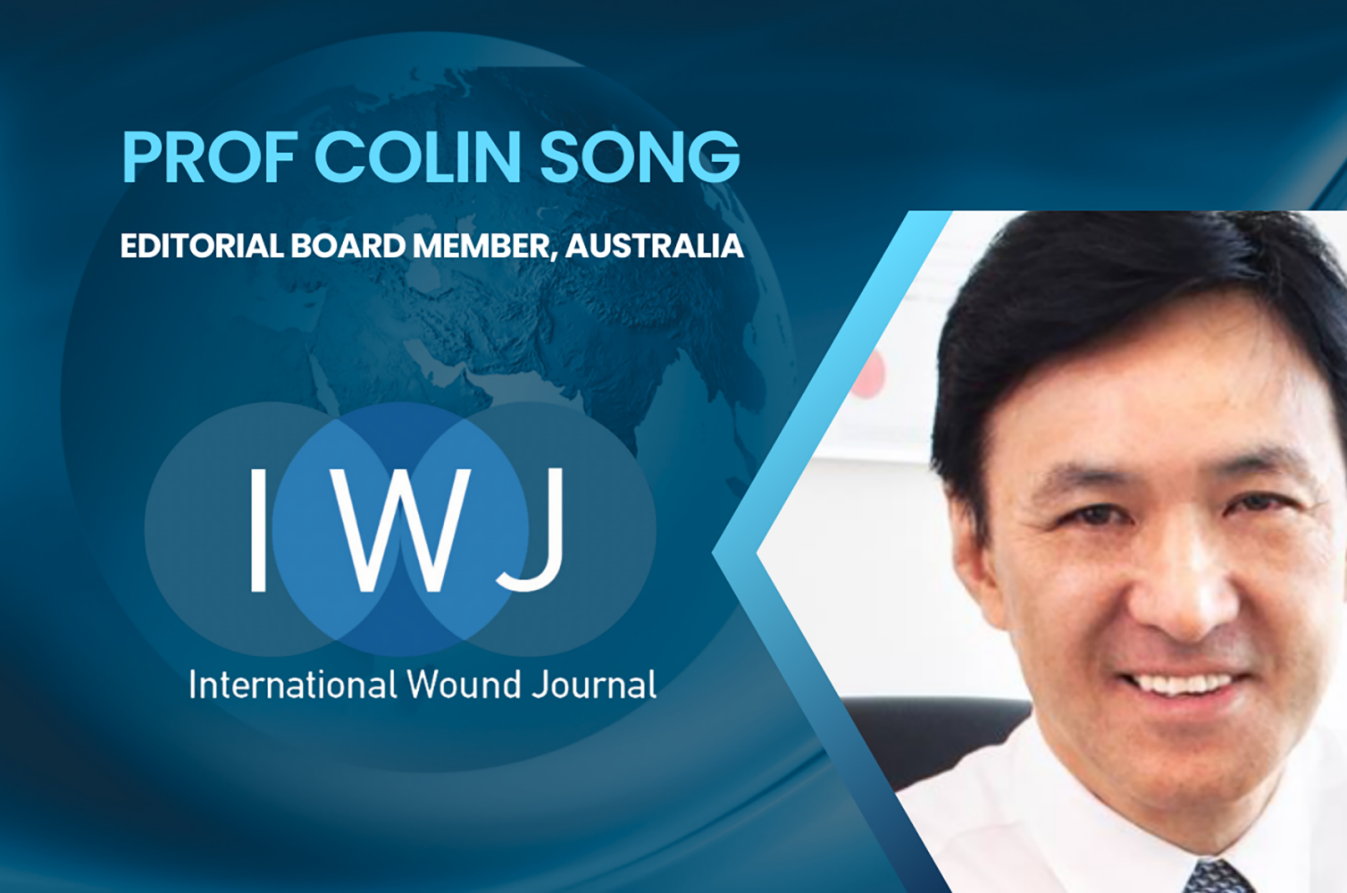
Professor Colin Song, Australia

Colin Song is a Professor in the Department of Plastic & Reconstructive Surgery at Fiona Stanley Hospital, Perth, Australia. His qualifications include MBBC, FRCS, FAMS, MScMEL and FRACS.

He is currently the Consultant Plastic, Reconstructive & Aesthetic Surgeon and former Head of Service at Fiona Stanley Hospital, Perth, Western Australia. Previously, he was the Medical Director: Cape Plastic Surgery, Senior Consultant & Head of Dept. Singapore General Hospital Plastic, Reconstructive & Aesthetic Surgery and the Director SGH Burn Centre, Singapore (1997–2012). His academic appointments include Adjunct Clinical Professor, Curtin University Medical School (present) and previously included Advisor, JCST Residency Accreditation Committee, Singapore; Professor, Duke‐National University of Singapore Graduate Medical School and National University Singapore Medical School; Inaugural President of the College of Surgeons, Academy of Medicine, Singapore; Chairman of Educational Subcommittee of Association of Plastic, Reconstructive & Aesthetic Surgeons of Southern Africa; and Head of Department of Plastic and Reconstructive Surgery, University of the Witwatersrand, Group of Teaching Hospitals, Johannesburg.

Colin is a member of the Editorial Board—*Journal of Plastic Reconstructive & Aesthetic Surgery* and the *International Wound Journal*. He is a past and founding‐president of the Asian Wound Healing Association & Wound Healing Society Singapore. Past President Association of Burn Injuries Singapore & Singapore Association of Plastic Surgeons. He has published three book chapters and 68 papers.

**Figure:**
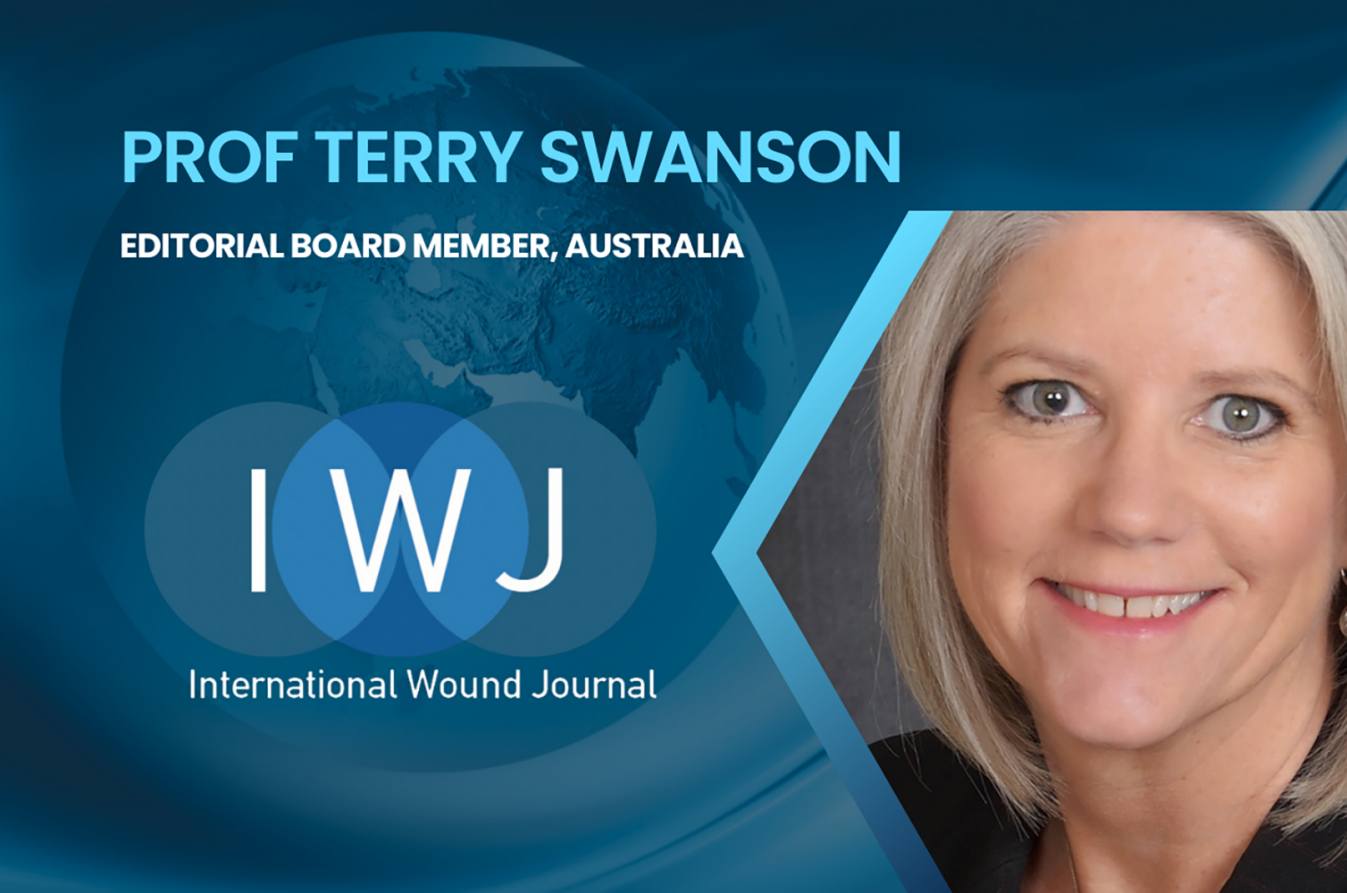
Professor Terry Swanson, Australia

Terry was admitted as a Fellow of the Australian Wound Management Association in 2010 for her significant contribution to wound management at a state, national and international level. She is the current Vice Chair of the International Wound Infection Institute (IWII) and chaired the development of the 2016 and the 2022 IWII Consensus Document Wound Infection in Clinical Practice. Terry has published and presented on chronic wounds and wound infection.

She states: ‘we have the same quest of improving care of the individual with a wound and a journal will help with the translation of science into practice’.

**Figure:**
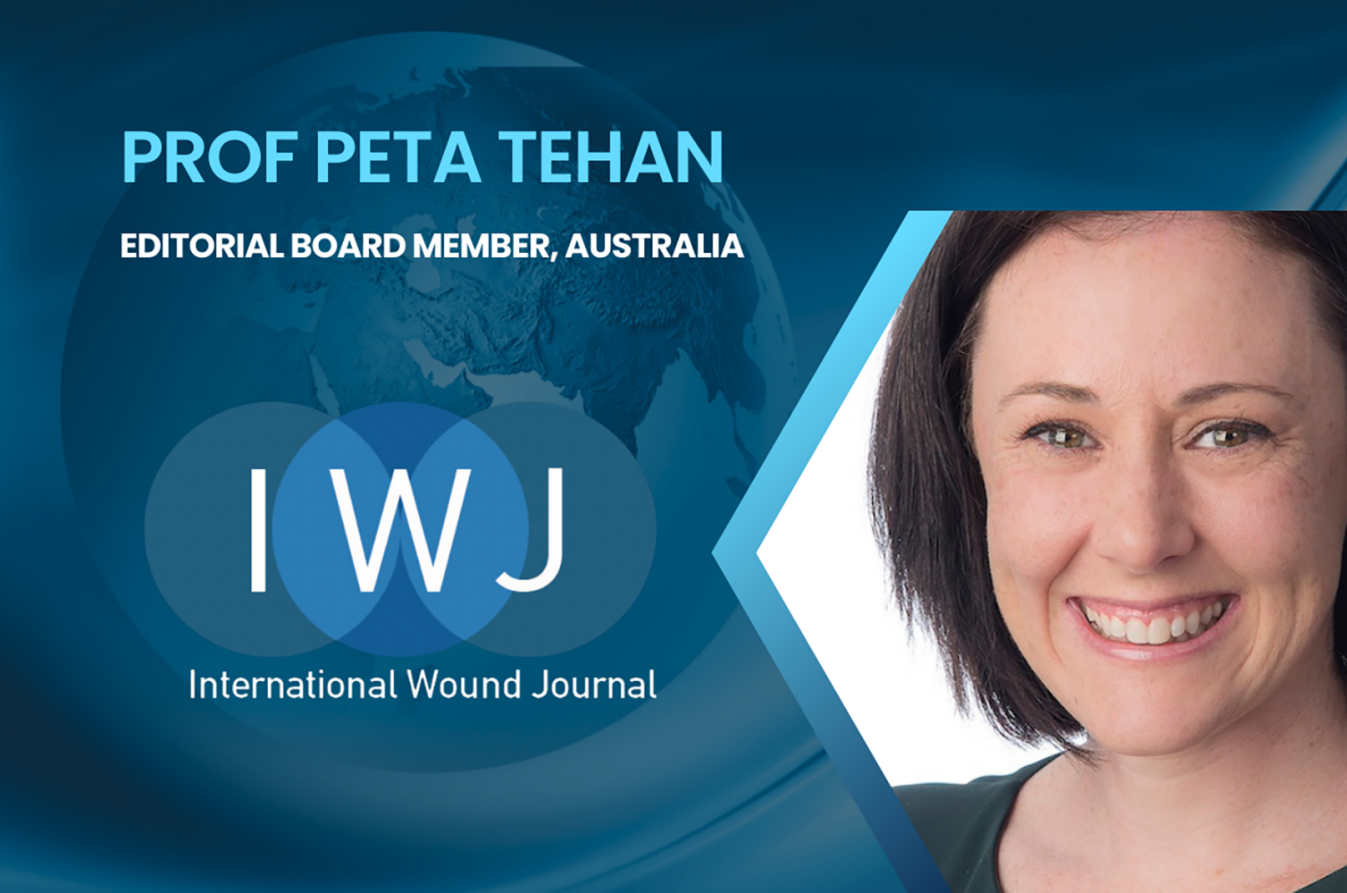
Professor Peta Tehan, Australia

Peta is a registered podiatrist who has worked in a variety of areas within podiatric practice for over 10 years, including private practice in both rural and regional areas and public practice in a high‐risk foot clinic. This gives her the real‐world experience that students appreciate in their clinical education and furthermore drives her passion for clinically translatable research outcomes.

Peta has post‐graduate qualifications in wound care from Monash University and frequently lectures to Wounds Australia and the local Hunter Wound Interest Group. Peta has been invited to edit the latest edition of the Therapeutic Guidelines on ‘Wound Care’—the high‐risk foot chapter. Peta's passion for wound care education was recognized by a Faculty of Health and Medicine community engagement award in 2016.

Her PhD (awarded August 2016) focused on podiatrists' role in vascular assessment of the lower limb and assessing the validity of non‐invasive vascular assessment techniques in Diabetes. Peta also developed a novel vascular screening pathway for community‐based patients. Peta's post‐doctoral research is building upon her thesis, continuing to develop the evidence base for non‐invasive vascular assessment in the lower limb in different, vulnerable populations. Furthermore, she recently completed a 6‐month international post‐doctoral fellowship with Professor Keith Rome at Auckland University of Technology which was focused on retail footwear use in women with rheumatoid arthritis.

*Research Expertise*: Peta is currently undertaking research investigating early and accurate identification of the presence of arterial disease in people with diabetes, assessing factors contributing to wound chronicity in people with diabetic foot ulceration, including dietary intake, and is also completing research examining clinically detectable vascular disease in people with rheumatoid arthritis. These projects have attracted both internal and external competitive funding.

Peta was awarded ‘Best New Investigator’ at the New Zealand Podiatry Conference in 2012, and best oral abstract at the Australian Diabetes Association Annual Meeting (2015—co‐author).

**Figure:**
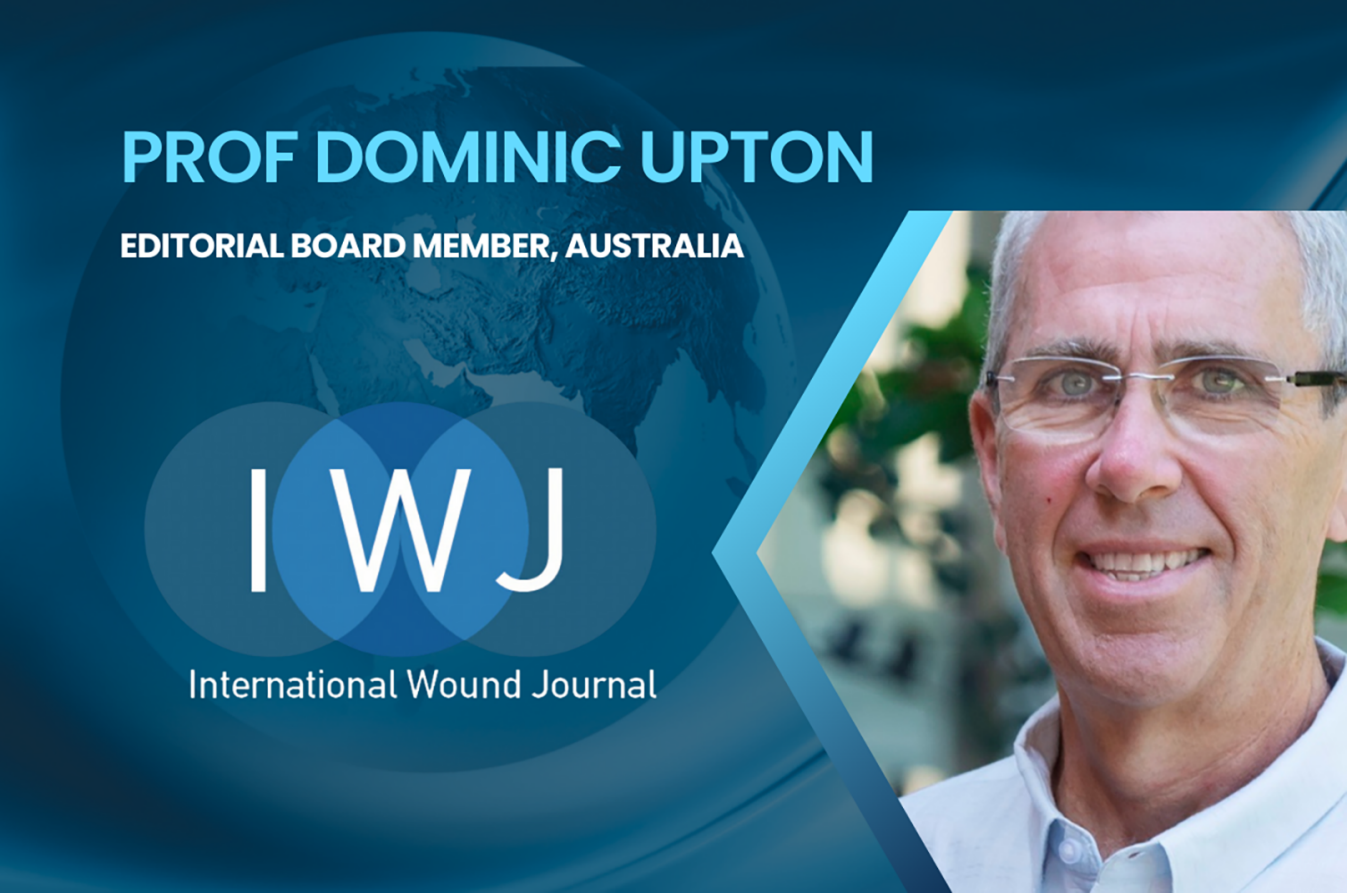
Professor Dominic Upton, Australia

Professor Dominic Upton is the Pro Vice‐Chancellor of the Faculty of Health at Charles Darwin University. In this role, he oversees the faculty's educational and research programs. He is leading the introduction of new programs to complement the existing portfolio along with building a coalition to develop the CDU School of Medicine. Professor Upton has led a number of significant research projects funded by national and international bodies. In 2005, he was elected Fellow of the British Psychological Society, awarded a National Teaching Fellowship in 2007 and was elected as Principal Fellow of the Higher Education Academy in 2018. He has authored 19 books and over 200 academic articles in academic journals and book chapters, being rated in the list of world's scientists who are in top 2% of their main subfield discipline (Nursing and Public Health). His research spans many areas of psychology related to wound healing and wound care.

**Figure:**
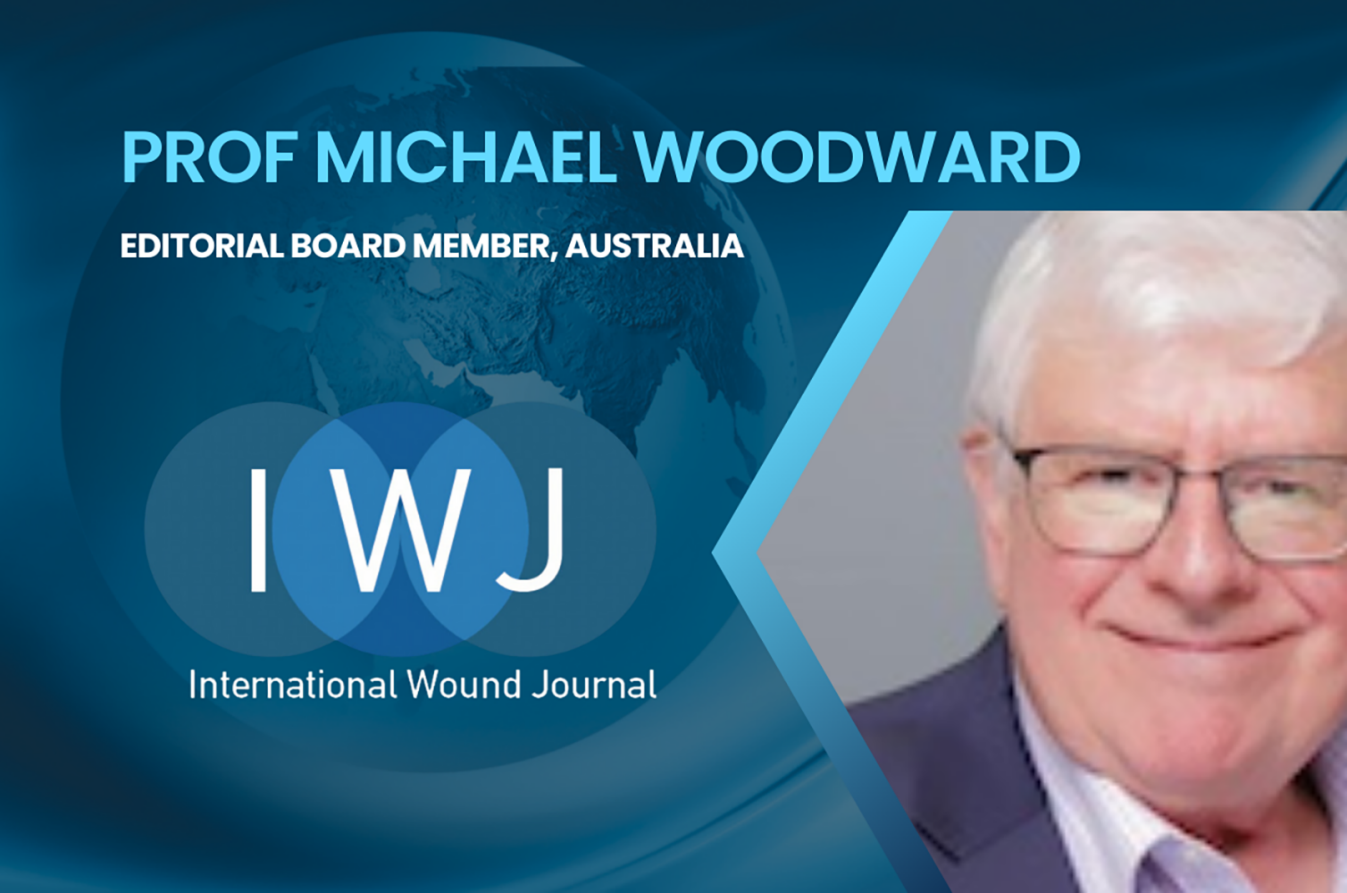
Professor Michael Woodward, Australia

Associate Professor Michael Woodward is Head of Aged Care Research and the Wound Management Clinic at Austin Health in Melbourne, Victoria, Australia. He is a past president and is honoured to be a Fellow of the Australian Wound Management Association, now Wounds Australia, on which he served as an inaugural Board Member in 2018. He leads several research trials investigating new therapies and other aspects of the management of chronic wounds.

Associate Professor Woodward's publication record includes 140 peer‐reviewed research papers, numerous book chapters and 390 scholarly addresses. He is past chair of the ‘Geriatric Therapeutics’ section of the editorial board of the *Journal of Pharmacy Practice and Research* and for 12 years, to the end of 2016, has been joint editor of *Wound Practice and Research*, the journal of Wounds Australia. He is also a member of the Repatriation Pharmaceutical Reference Committee, which approves drugs and dressings for Australian Veterans, and chairs their Advisory Wound Care Committee that recently released the revised DVA Wound Care manual and on‐line wound dressings chart.

He is also a Fellow of the Australian and New Zealand Society for Geriatric Medicine and is a previous chair of their Education and Training Subcommittee. He is also a Fellow of the Australian Association of Gerontology, having previously been President of their Victorian Division, and is a lifetime honorary member of Wounds Australia Victoria. He Chairs the Accreditation Subcommittee of the Royal Australasian College of Physician, responsible for accrediting training sites and programs across Australia.

On Australia Day 2016, Michael was honoured to receive Membership of the Order of Australia for his work in geriatric medicine and as an author.

**Figure:**
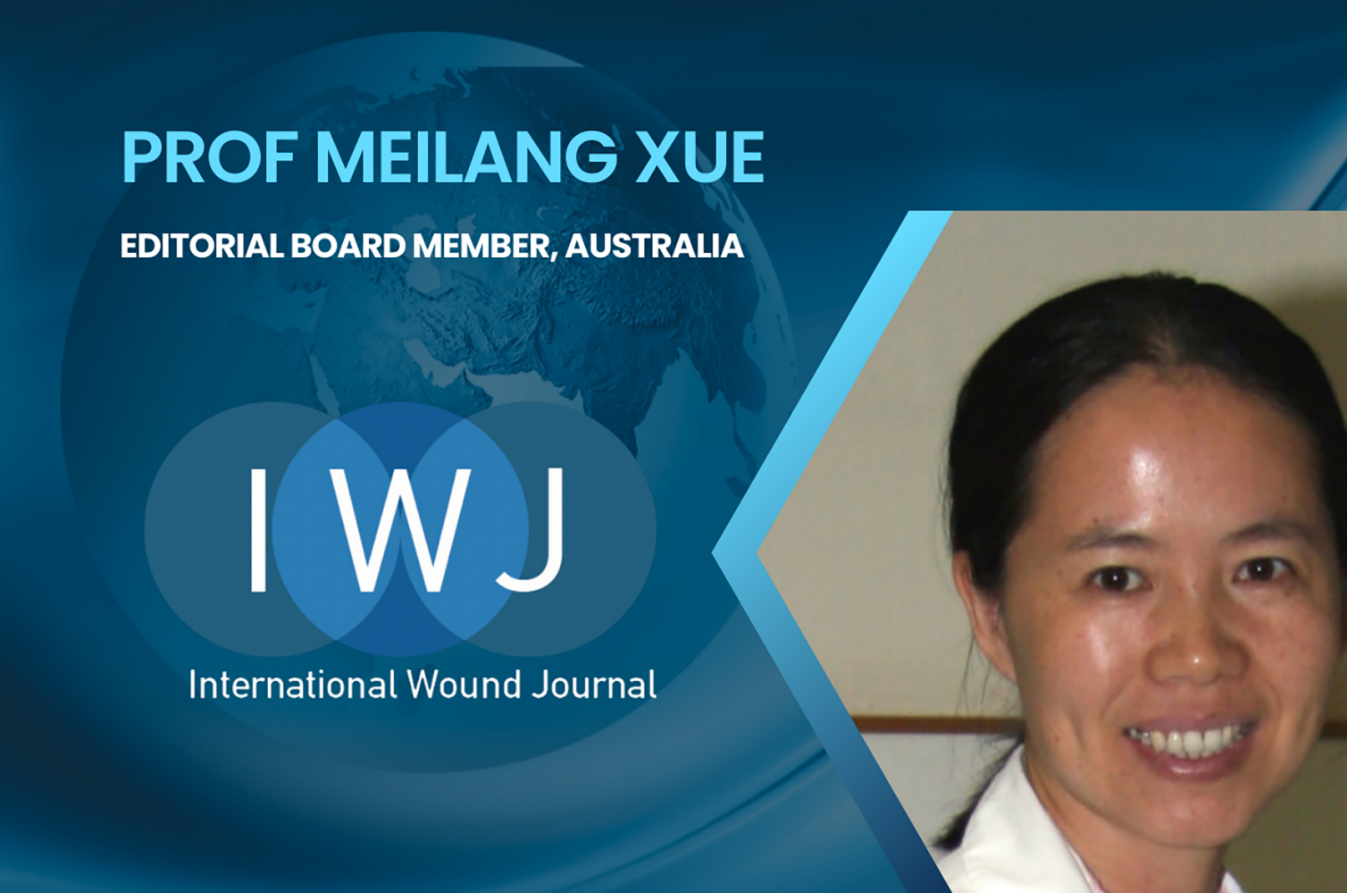
Professor Meilang Xue, Australia

Dr. Meilang Xue is an Associate Professor at the Sutton Arthritis Research Laboratory, Sydney Musculoskeletal Health, Kolling Institute, and Faculty of Medicine and Health, The University of Sydney, NSW, Australia. Dr. Xue's research is primarily focused on inflammatory and autoimmune diseases, with a special emphasis on inflammatory arthritis and wound healing, which are her areas of expertise.

**Figure:**
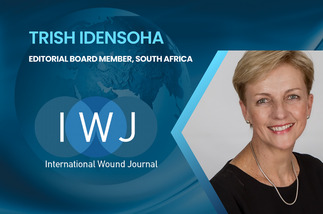
Trish Idensoha, South Africa and UK

Trish is an independent wound consultant and wound specialist in private practice. She is also the Director of Comp Consulting Limited, United Kingdom. She is the owner of CliniCare Medical Centre, Ballito, South Africa, providing interdisciplinary wound management in the clinic, hospital, sub‐acute and home environments. She is also a research collaborator with Stellenbosch University, Stellenbosch, Western Cape, South Africa. Currently, she is an elected committee member of the International Wound Infection Institute Committee (IWII) and was on the development team for the 2022 International Consensus Update: Wound Infection in clinical practice.

She is a panel group member of the development team for the 2025 International Pressure Injury guidelines: part of the International peer review panel for Wound Healing Advances and Management collaborative (WHAM), Curtin University, Australia, and Editorial Board member of Wound Masterclass and the International Wound Journal.

Trish is a past regional and national president and an honorary member of the Society of Private Nurse Practitioners (SPNP), South Africa; past honorary tutor at Cardiff University, Wales, United Kingdom; past African Director for the International Skin Tear Advisory Panel (ISTAP); past Principal Lecturer and Coordinator of the Wound Certification Course for the University of the Free State, Bloemfontein, South Africa.

Trish combines her clinical practice experience, academia and research to enhance patient care by empowering healthcare practitioners to ‘be the change’ to improve patient outcomes. Her areas of interest include ‘all things wounds’ encompassing the array of wound types, including the prevention and management of skin integrity; wound infection; the prevention and management of hard‐to‐heal wounds and overcoming the holistic challenges wound management and wound care present.

**Figure:**
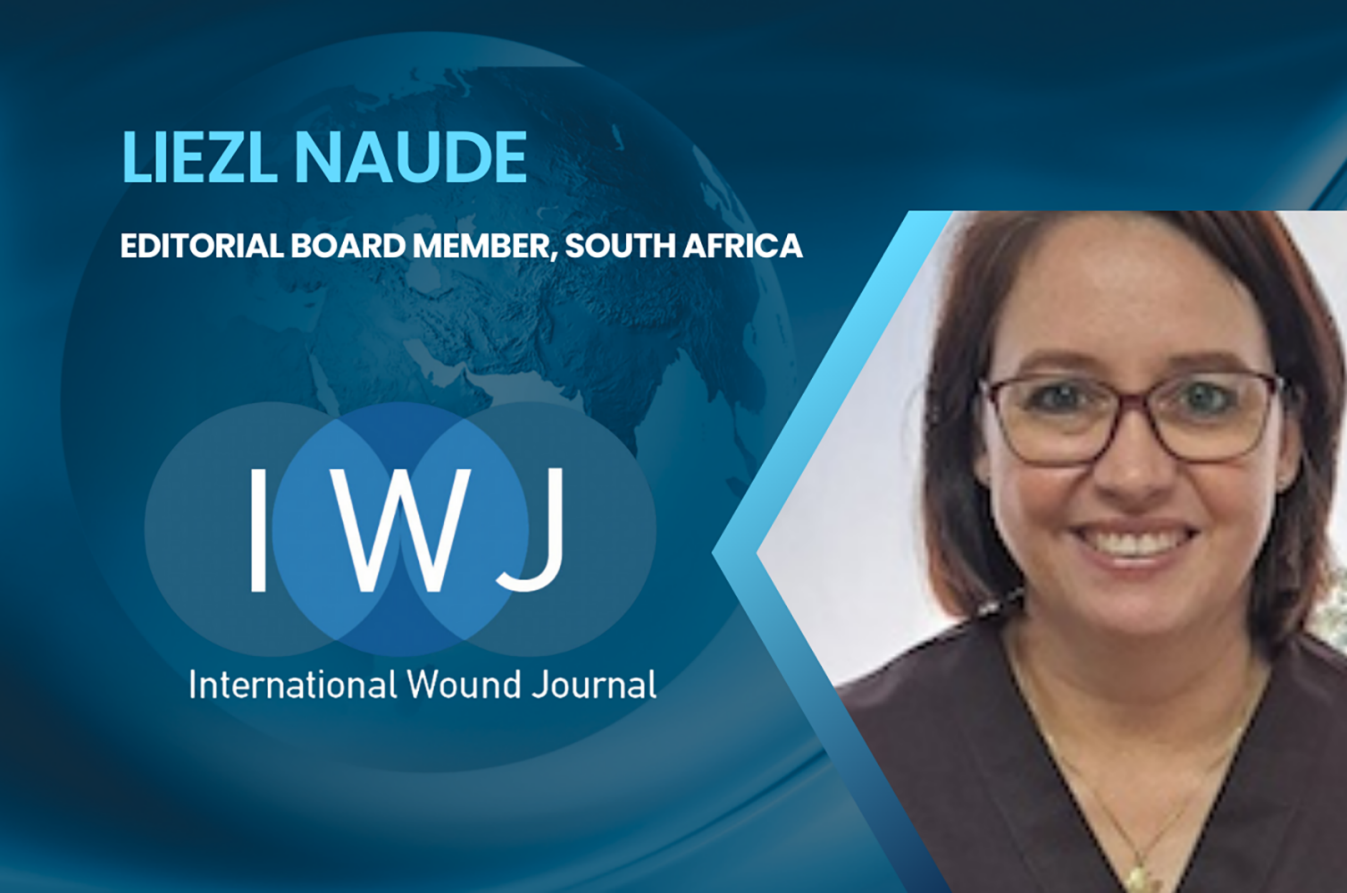
Liezl Naude, South Africa

Liezl is a Clinical Nurse Specialist and Independent Consultant in Advanced Wound Care, founder of Eloquent Learning Health & Eloquent Health & Wellness in Pretoria, South Africa. With extensive experience in wound management, Liezl serves as Clinical Director for Eloquent Health & Wellness and Chief Lecturer at Eloquent Learning Health training academy. She is a founding member of the Wound Healing Association of Southern Africa (WHASA) and the World Alliance of Wound and Lymphoedema Care (WAWLC). Recognized for her contributions, Liezl has received honorary life membership from WHASA and international nurse leader recognition from the International Nurses Association (INA). She is a prolific presenter and published author in the field, specializing in diabetic foot, lower limb management, complicated wounds and sports injuries. Passionate about making a difference, Liezl is actively involved in global wound.

**Figure:**
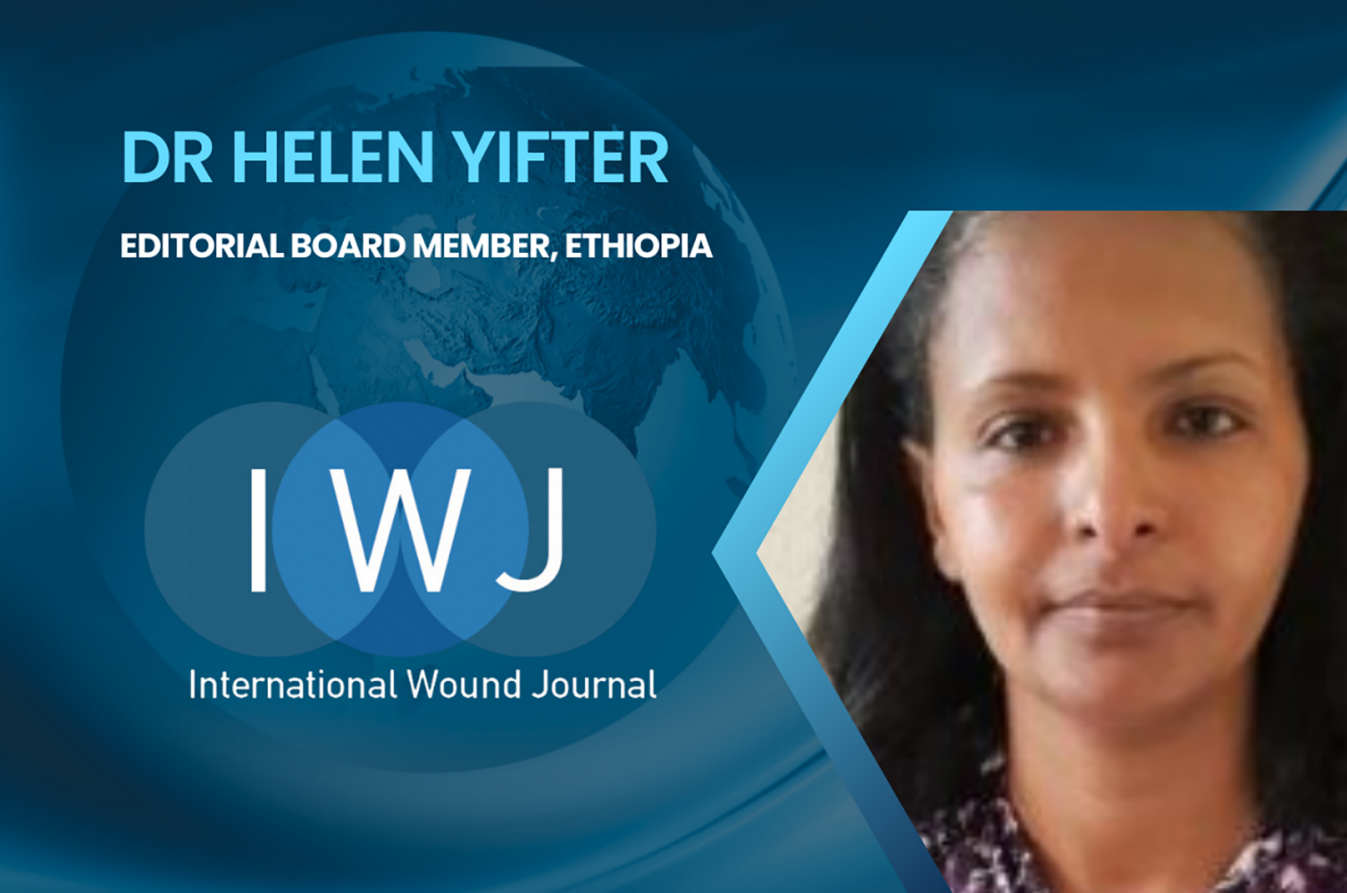
Dr. Helen Yifter, Ethiopia

Helen has more than 20 years of experience in health care service provision, education, academic leadership, research and specialized service development. She has been engaged in the teaching of undergraduate medical students, internal medicine residents and endocrinology fellows providing lectures, seminars and clinical bedside teachings. Helen is also an international faculty for the International Interprofessional Wound Care course in the United Arab Emirates (IIWCC‐UAE).

Her academic leadership experience includes chief resident of internal medicine, coordination of undergraduate teaching at the department of internal medicine, Associate Dean of the School of Medicine and Academic Director of the College of Health Sciences at Addis Ababa University. As an Academic Director, she was responsible for overseeing the academic functions of four schools under the college, namely, the schools of medicine, pharmacy, public health and nursing and midwifery, which combined close to 130 academic programs.

Since April 2021, she took a sabbatical leave from Addis Ababa University and is working at the University of Rwanda as the endocrinology fellowship program director. There she has been leading the curriculum development for the fellowship program and coordinating the training.

Helen was a co‐Principal Investigator in the African Health Observatory Platform Ethiopia (AHOP‐Ethiopia) which is a regional partnership to promote evidence‐informed policymaking by supporting cross‐countries learning.

Her recent research is focused on the integration of non‐communicable diseases (mainly diabetes and hypertension) services at the primary level. The output is to be published. She has also advised and examined several master's and PhD students.

As an endocrinologist and wound care specialist, she established the first foot care service in Ethiopia at Tikur Anbessa Hospital Diabetes Center. She facilitated the training of six nurses and nine doctors in foot care in collaboration with colleagues from the University of Toronto. The foot clinic provides treatment for more than 200 patients with diabetic foot ulcers per year, screening and follow‐up of patients with high‐risk foot and in‐person foot care education for patients coming for regular follow‐up at the diabetes centre.

She has been involved in the development of guidelines for the care of non‐communicable diseases (NCDs) and training modules (diabetes) at the Federal Ministry of Health of Ethiopia. She has also contributed a book chapter for the seventh edition of *Endocrine Secrets* and the 1st edition of *Diabetes Secrets*.

As part of her role as an advocate for patients and professions, she served as a board member of the Ethiopian Diabetes Association and an executive member of the Ethiopian Medical Association and Ethiopian Society of Internal Medicine. She is also a member of the international affairs committee of the World Union of Wound Healing Societies and Editorial Board member of the *Journal of Clinical Endocrinology and Metabolism* case reports.

In her Academic Director position, her flagship initiative was the establishment of the ‘Gender platform’, through which she was able to give training to female faculty on ‘Women's talent development’ and organize college‐wide gender dialogue in collaboration with Maastricht School of Management.

As the editorial team, we are excited to expand our board to help maintain the high quality standards of the *International Wound Journal* moving forward. We are proud of the diversity of our board, making it appropriate for the international reach of the journal.

## References

[1] Harding K , Queen D . Expansion of our editorial board. Int Wound J. 2024;21:e70010. doi:10.1111/iwj.70010

[2] Queen D , Harding K . Meet our North American editorial board members. Int Wound J. 2024;21:e70061. doi:10.1111/iwj.70061

[3] Harding K , Queen D . Meet our European editorial board members. Int Wound J. 2024;21:e70085. doi:10.1111/iwj.70085

